# Assessing the daily natural history of asymptomatic *Plasmodium* infections in adults and older children in Katakwi, Uganda: a longitudinal cohort study

**DOI:** 10.1016/S2666-5247(23)00262-8

**Published:** 2024-01

**Authors:** Dianna E B Hergott, Tonny J Owalla, Weston J Staubus, Annette M Seilie, Chris Chavtur, Jennifer E Balkus, Bernadette Apio, Jimmy Lema, Barbara Cemeri, Andrew Akileng, Ming Chang, Thomas G Egwang, Sean C Murphy

**Affiliations:** a Department of Laboratory Medicine and Pathology, University of Washington, Seattle, WA, USA; b Department of Epidemiology, School of Public Health, University of Washington, Seattle, WA, USA; c Center for Emerging and Re-emerging Infectious Diseases, University of Washington, Seattle, WA, USA; d Department of Microbiology, University of Washington, Seattle, WA, USA; e Department of Parasitology and Immunology, Med Biotech Laboratories, Kampala, Uganda

## Abstract

**Background:**

Low-density asymptomatic *Plasmodium* infections are prevalent in endemic areas, but little is known about their natural history. The trajectories of these infections and their propensity to fluctuate to undetectable densities can affect detection in clinical trials and field studies. We aimed to classify the natural history of these infections in a high transmission area over 29 days.

**Methods:**

In this longitudinal cohort study, we enrolled healthy, malaria-asymptomatic, afebrile, adults (age 18–59 years) and older children (age 8–17 years) in Katakwi District, Uganda, who were negative for *Plasmodium* infection on rapid diagnostic tests. Participants were instructed to self-collect one dried blood spot (DBS) per day for a maximum of 29 days. We excluded people if they were pregnant or taking antimalarials. During weekly clinic visits, staff collected a DBS and a 4 mL sample of venous blood. We analysed DBSs by *Plasmodium* 18S rRNA quantitative RT-PCR (qRT-PCR). We classified DBS by infection type as negative, *P falciparum*, non-*P falciparum*, or mixed. We plotted infection type over time for each participant and categorised trajectories as negative, new, cleared, chronic, or indeterminate infections. To estimate the effect of single timepoint sampling, we calculated the daily prevalence for each study day and estimated the number of infections that would have been detected in our population if sampling frequency was reduced.

**Findings:**

Between April 9 and May 20, 2021, 3577 DBSs were collected by 128 (40 male adults, 60 female adults, 12 male children, and 16 female children) study participants. 2287 (64%) DBSs were categorised as negative, 751 (21%) as positive for *P falciparum*, 507 (14%) as positive for non-*P falciparum*, and 32 (1%) as mixed infections. Daily *Plasmodium* prevalence in the population ranged from 45·3% (95% CI 36·6–54·1) at baseline to 30·3% (21·9–38·6) on day 24. 37 (95%) of 39 *P falciparum* and 35 (85%) of 41 non-*P falciparum* infections would have been detected with every other day sampling, whereas, with weekly sampling, 35 (90%) *P falciparum* infections and 31 (76%) non-*P falciparum* infections would have been detected.

**Interpretation:**

Parasite dynamics and species are highly variable among low-density asymptomatic *Plasmodium* infections. Sampling every other day or every 3 days detected a similar proportion of infections as daily sampling, whereas testing once per week or even less frequently could misclassify up to a third of the infections. Even using highly sensitive diagnostics, single timepoint testing might misclassify the true infection status of an individual.

**Funding:**

US National Institutes of Health and Bill and Melinda Gates Foundation.

## Introduction

The complex life cycle of *Plasmodium* parasites results in highly dynamic infections, with densities that can change more than 90-fold over several hours.^1^ As such, *Plasmodium* densities can drop below the limit of detection of routinely available clinical diagnostic tools, such as thick blood smears and antigen-based rapid diagnostic tests (RDTs) during the course of infection.^1^, ^2^, ^3^, ^4^ Nevertheless, most epidemiological studies of malaria burden and clinical trial protocols for malaria vaccines and therapeutics use single-timepoint sampling to identify the malaria infection status of an asymptomatic individual, which is an approach that is likely to miss infections if they fluctuate below the detection limit of the assay used.^5^^,^^6^ Several studies have challenged the accuracy of a single measurement for infection status;^1^^,^^3^^,^^4^^,^^7^^,^^8^ however, the quantity and quality of evidence on the dynamics of subpatent, asymptomatic *Plasmodium* infections are poorly understood.

**Research in context**

**Evidence before this study**

We searched PubMed for human studies published in the English language from database inception to July 21, 2022, using the search terms: (((*Plasmodium* AND *falciparum*) OR malaria)[Title/Abstract])) AND (dynamic∗ OR (natural history) OR longitudinal)[Title/Abstract] AND (daily[Title/Abstract] OR repeat[Title/Abstract] OR day[Title/Abstract] OR single[Title/Abstract]). Five studies were identified that have looked at the daily dynamics of non-treated *Plasmodium* infections in a total of 66 individuals. Studies were done in Tanzania, Senegal, Ghana, Mali, and Burkina Faso. In all five studies, asymptomatic infections were identified by thick blood smear and then tracked using thick blood smear or PCR methods. Sampling length ranged between 3 days and 6 weeks. All five studies showed large daily variations in parasite densities.

**Added value of this study**

To our knowledge, this is the largest study looking at the daily dynamics of asymptomatic infections in an endemic population and the first to use quantitative RT-PCR for parasite detection. Except for one study that collected samples from a single participant for 6 weeks, this is also the longest study of daily dynamics of infection and the first to examine dynamics in a group that includes female adults. Our study provides valuable insights into the variation in the kinetics and magnitude of *Plasmodium* infections in an area of high transmission and provides a feasible solution to expand this type of sampling to larger populations.

**Implications of all the available evidence**

Our study strengthens the evidence that sampling at a single timepoint is insufficient to reliably detect infection in individuals who are asymptomatic. Given the highly dynamic nature of low-density infections, serial testing should be considered when determining the true infection status of an individual. These findings might have implications for the design of clinical trials and epidemiological field studies where asymptomatic infections could influence the outcome.

In 2018, Drakeley and colleagues^9^ outlined several potential outcomes and theoretical trajectories of a low-density *Plasmodium* infection captured at a single point in time, including becoming symptomatic, clearing, or remaining chronic and fluctuating in density. Each varied trajectory could contribute differently to the infectious reservoir in a region. An improved understanding of infection dynamics over time could enhance targeted control strategies and reduce malaria burden but there is a scarcity of prospectively collected data describing the actual proportion and profile of each of these types of infections in malaria-endemic areas.

The paucity of data on the daily dynamics and natural history of asymptomatic *Plasmodium* infections is mostly due to the logistical complexity and financial constraints of daily clinical sampling. However, using data from the same study, we recently published that self-collected dried blood spots (DBSs) are acceptable and reliable for studying the daily quantitative dynamics of *Plasmodium* infections in adults and older children.^10^ In this study, we evaluated infection profiles and dynamics of asymptomatic, low-density *Plasmodium* infections using DBSs self-collected by participants daily for 29 days from adults and older children in Katakwi District, Uganda. The results were used to estimate the proportion of all infections that might be missed using single timepoint sampling.

## Methods

### Study design and participants

This was a longitudinal cohort study carried out in Katakwi District in northeastern Uganda between March 31 and May 20, 2021. From March 31 to April 22, participants were recruited from two villages near St Anne Health Center III (Katakwi District, Uganda) through community engagement meetings and mobilisation led by study staff and aided by village health team members and local council chairpersons. A detailed description of the study design, area, and population was published previously^10^ and the full study protocol is available online. All homesteads were registered and homesteads or individuals were invited to a screening visit through random selection. To be enrolled, participants had to be asymptomatic and negative for malaria using the SD Malaria Ag Pf/Pan RDT (Standard Diagnostics, Suwon, South Korea) at enrolment, have no clinically significant chronic diseases, and be willing to follow study procedures. Being pregnant and currently taking antimalarial treatment were the main exclusion criteria. We included participants who were aged 8–59 years.

The study was approved by the National HIV/AIDS Research Committee of the Uganda National Council for Science and Technology (UNCST; ARC 228) and the University of Washington institutional review board (STUDY00009434). Adult participants provided written consent. Children provided assent and a parent or guardian provided written consent, per UNCST guidelines.

### Procedures

In brief, participants were taught how to self-collect DBSs and then were asked to self-collect a daily DBS (roughly 50 μL of blood) for 29 days. Six DBSs were self-collected at home each week between weekly clinic visits. During weekly clinic visits, staff collected a DBS and a sample containing 4 mL of venous blood. Participants who developed any malaria signs or symptoms during the study were tested by RDT. Participants positive for malaria were treated following the Ugandan national guidelines and withdrawn from further DBS collections. All data on demographics and sex were self-reported. We did not record the ethnicity of the participants.

DBSs from participants were sent to the University of Washington Malaria Molecular Diagnostic Laboratory (Seattle, WA, USA) where individual DBSs were excised using contact-free laser-cutting methods^11^ and analysed by multiplex quantitative RT-PCR (qRT-PCR) targeting two *Plasmodium* 18S rRNA sequences: one conserved across human-infecting *Plasmodium* species and one specific to *Plasmodium falciparum*.^12^ Nucleic acids were extracted from pools of ten or fewer individual DBSs from the same participant and subjected to qRT-PCR. Samples from positive pools were tested individually and those from negative pools were all considered negative.^10^^,^^13^

As an exploratory analysis, venous blood from non-*P falciparum* infections containing more than 55 000 parasites per mL with sufficient residual material were selected for Sanger sequencing for species identification. qRT-PCR products were purified with QIAquick PCR Purification Kit (Qiagen, Germantown, MD, USA) and submitted to GeneWiz (Azenta Life Sciences, Seattle, WA, USA). Sense and antisense sequence pairs were assembled against reference 18S sequences of *P falciparum* (GenBank M19172.1), *Plasmodium ovale wallikeri* (KF219561), *P ovale curtisi* (KF696373), *Plasmodium knowlesi* (L07560), *Plasmodium malariae* (XR_003751948), and *Plasmodium vivax* (XR_003001206) using CLC Main Workbench software (8.1.3; QIAGEN, Aarhus, Denmark) to determine species identification. Samples without a consensus sequence due to absence of overlap or absence of assembly against a reference sequence were examined individually for evidence of mixed *Plasmodium* infections.

### Outcomes

The primary outcome was the presence and classification of *Plasmodium* 18S rRNA in individual DBSs. We classified DBS infection types based on measured densities of pan-*Plasmodium* (Pan; ie, any *Plasmodium* species) and *P falciparum*-specific targets in each spot. DBSs in which the measured densities of the *P falciparum* targets were within an order of magnitude of the density of the Pan target were classified as *P falciparum* infections. DBSs in which the density of the pan-*Plasmodium* target exceeded that of the *P falciparum* target by an order of magnitude or more were classified as mixed. DBSs in which only the Pan target was detected were classified as non-*P falciparum*. DBSs in which neither target was detected were classified as negative.

We categorised and evaluated infection profiles over the study period, based on the patterns of the classifications of the individual DBS. For individuals with at least one positive DBS during the study, primary categories included *P falciparum* only (all DBSs were classified as *P facliparum*), non*-P falciparum* only (all DBSs were classified as non-*P falciparum*), or mixed infections. Mixed infections included those with all individual DBSs classified as mixed (both *P falciparum* and non-*P falciparum* infections at the same time), as well as those with a *P falciparum* infection at one point in the study and a non-*P falciparum* infection at another. Participants with no positive DBSs during the study period were categorised as negative. Within each major category, where possible, we further classified infections as new infections (negative for at least the first 3 days of study and positive after), cleared infections (positive during the first DBS [baseline] and then negative for ≥7 days), or chronic oscillating infections (positive at baseline and throughout the study period). Profiles were independently classified by two study staff members (DEBH and SCM), and discrepancies were discussed and resolved. If a discrepancy could not be resolved by these study staff members, if the infection had fewer than five timepoints, or if the pattern was not described by a subcategory, the profile was classified as indeterminate. As secondary outcomes, we calculated the prevalence of *P falciparum*, non-*P falciparum*, mixed, and all *Plasmodium* infections by study day and the number and proportion of positive DBSs that were above 100 000 parasites per mL.

### Statistical analysis

We reported the total number of DBSs collected as mean DBS per person, and *Plasmodium* classifications of all individual DBSs collected during the study by age group (ages 18–59 years and 8–17 years) and sex (male or female). We calculated the number and proportion of each infection profile primary category and subcategory. The study was powered to detect an overall prevalence of asymptomatic infection of 40% in the catchment area of 2000 individuals. The main study was powered to determine compliance of 70% (SD 9). To explore the possibility of these low-density infections becoming detectable by RDT, we analysed the number and proportion of all positive DBSs with estimated parasite densities of 100 000 parasites per mL of blood or more (a density that should be detected as positive in most RDTs^14^). We describe infection profiles of individuals who developed symptomatic, RDT-positive infections during the study period.

To understand the possible diagnostic consequences of testing at a single timepoint, we conducted several analyses. First, we calculated the prevalence (95% CI) of *P falciparum*, non-*P falciparum,* and mixed infections identified on each study day. Additionally, among participants who had a positive DBS at any point throughout the follow-up period, we calculated the number of days individuals had a negative test and presented the median (IQR) by age group and sex. For each study day, we calculated the probability that an individual who had a negative test was positive on the following day, as well as the probability that an individual who was positive was negative the following day. The minimum and maximum probabilities and their 95% CIs are reported. Finally, we estimated the effect of less frequent sampling strategies on capturing infections identified over three different periods of time: 7 days, 21 days, and 29 days. Only participants with complete DBS sets (ie, 29 DBSs) were included in all analyses. Infection was defined as one or more positive DBSs during the defined period of time. *P falciparum* and non-*P falciparum* infections were analysed separately and mixed DBSs counted as positive for both. Within a particular period, we calculated the proportion of infections detected by daily sampling (29 days of DBSs) that would have been detected had samples been collected less frequently, including every other day, every third day, weekly, every two weeks, or at the beginning and end of the period only. For the 7 day and 21 day sampling periods, we performed the analysis in rolling windows, changing the first day of the time period, to account for the possible effect of sampling start day on the probability of capturing infection. Details about the sampling schemes are presented in the appendix (p 1). Boxplots are presented to show the range of the proportion of infections captured with each start day. All analyses were performed in R (3.6.2).

### Role of the funding source

The funders of the study had no role in study design, data collection, data analysis, data interpretation, or writing of the report.

## Results

Of 236 participants screened, 131 were enrolled between April 9 and April 22, 2021, and collected at least one DBS. Of the 131 participants enrolled, DBS cards were received for qRT-PCR processing at the University of Washington Malaria Molecular Diagnostic Laboratory from 100 adults (aged 18–59 years; 40 male adults and 60 female adults) and 28 children (aged 8–17 years; 12 male children and 16 female children) and were included in the primary analysis. DBS cards from three participants were collected but not received at the laboratory for qRT-PCR processing. There were 3577 total DBSs (2953 self-collected and 624 unique clinic-collected) during the study (table 1), with a mean of 27·9 DBSs (SD 4·0) collected per person. On average, female adults and children collected more DBSs than male adults and children (28·7 [SD 1·1] in female adults *vs* 26·8 [6·5] in male adults and 28·1 [2·4] in female children *vs* 27·7 [7·4] in male children), which was mostly due to some early discontinuations in male participants.^10^ 3209 [89·7%] of 3577 DBSs had 40 μL of blood or more, as reported.^10^ Of 3577 DBSs, 2287 (63·9%) were categorised as negative, 751 (21·0%) were categorised as *P falciparum* infection, 507 (14·1%) were categorised as non-*P falciparum* infection, and 32 (1·0%) were categorised as mixed infections (*P falciparum* and other *Plasmodium* species). Negative DBSs were more common in female adults (1248 [72·4%] of 1724) compared with adult males (611 [57·0%] of 1072), whereas male children had more negative DBSs (202 [60·8%] of 332) than female children (226 [50·3%] of 449; table 1).

**Table 1:** Select characteristics of the total number of participants, DBSs, mean DBSs collected over the study period, and number and proportion of DBSs that were categorised as negative, positive for *Plasmodium falciparum*, positive for non-*P falciparum*, or mixed infections

	Male adults∗	Female adults∗	Male children∗	Female children∗	Total
Demographics					
Participants	40 (31·2%)	60 (46·9%)	12 (9·4%)	16 (12·5%)	128
Median age (IQR)	30 (23–40)	29 (25–44)	13 (12–15)	13 (11–16)	26 (19–39)
DBS results					
Total unique DBSs collected†	1072 (30·0%)	1724 (48·2%)	33 (9·3%)	449 (12·6%)	3577
Mean spots per person (SD)	26·8 (6·5)	28·7 (1·1)	27·7 (7·4)	28·1 (2·4)	27·9 (4·0)
Negative DBS‡	611 (0·6)	1248 (0·7)	202 (0·6)	226 (0·5)	2287 (0·6)
*P falciparum*-positive DBS‡	273 (0·3)	244 (0·1)	71 (0·2)	163 (0·4)	751 (0·2)
Pan-*Plasmodium*-positive (non-*P falciparum*) DBS‡	167 (0·2)	228 (0·1)	52 (0·2)	60 (0·1)	507 (0·1)
Mixed (*P falciparum* and non-*P falciparum*) DBS‡	21 (0·0)	4 (0·0)	7 (0·0)	0 (0)	32 (0·0)

Data are n (%), unless otherwise stated. DBS=dried blood spot.

∗ Sex assigned at birth was self-reported by participants at screening.

† A unique spot is one collected at a unique location, date, and participant.

‡ Presented as n (proportion of total unique spots by group).

Non-*P falciparum* species identification was successful in 17 of 20 samples from 15 individuals and showed seven *P ovale wallikeri,* four *P ovale curtisi,* and six *P malariae* positive samples. Three samples that could not be definitively sequenced showed sequence-based evidence of mixed infections (data not shown).

Among 128 participants, 77 (60·2%) had at least one positive DBS during the study (table 2). Of those with infections, 28 (36·4%) of 77 had infection profiles that were *P falciparum* only, 29 (37·7%) had infections that were non-*P falciparum* only, and 20 (26·0%) had infections with both *P falciparum* and non-*P falciparum* parasites throughout the study.

**Table 2:** Cumulative prevalence of *Plasmodium* infections in the study population during the 29 day period, by sex and age group

	Male adults (n=40)	Female adults (n=60)	Male children (n=12)	Female children (n=16)	All (n=128)
*Plasmodium falciparum* only	8 (20%)	11 (18%)	2 (17%)	7 (44%)	28 (22%)
Pan-*Plasmodium* only	9 (23%)	12 (20%)	4 (33%)	4 (25%)	29 (23%)
Mixed infection	10 (25%)	6 (10%)	3 (25%)	1 (6%)	20 (16%)
All positives	27 (68%)	29 (48%)	9 (75%)	12 (75%)	77 (60%)

Adults were aged 18–59 years and children were aged 8–17 years.

**Figure 1:**
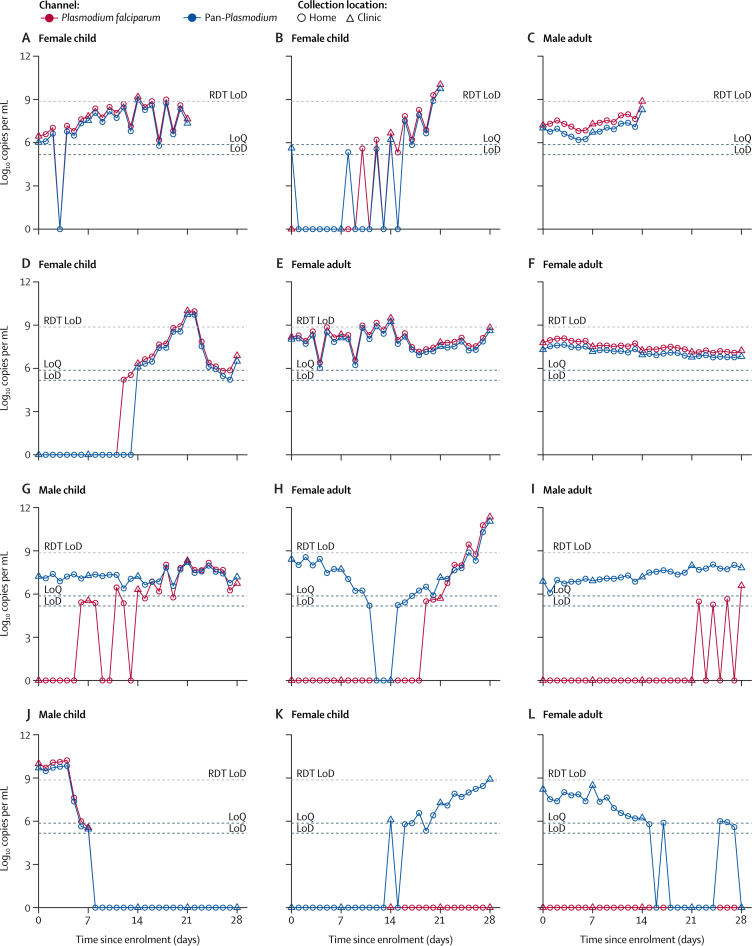
Daily parasite density patterns for selected participants (A–C) Three examples of *Plasmodium falciparum* infection with sawtooth patterns culminating in clinical illness and malaria diagnosis. (D) An example of a new *P falciparum* infection without clinical illness. (E, F) Two examples of continuous *P falciparum*-only infection without clinical illness. (G–I) Three examples of pre-existing non-*P falciparum* infection joined by a new *P falciparum* infection. (J) An example of clearing *P falciparum* infection. (K) An example of a new non-*P falciparum* infection. (L) An example of a clearing non*-P falciparum* infection. RDT=rapid antigen-detection diagnostic tests. LoD=limit of detection. LoQ=limit of quantification.

Complete trajectories of infection time courses for all infected individuals are in the appendix (pp 2–78). For the 28 *P falciparum-*only infections (appendix pp 2–29), four were newly acquired infections (appendix pp 26–29), seven were pre-existing infections that cleared (appendix pp 16–22), 14 were chronic oscillating infections (appendix pp 2–15), and two were indeterminate (appendix pp 24–25). One participant had an existing *P falciparum* infection at baseline that cleared on day 12 and then had a new infection on day 28 (appendix p 23). Among 29 non*-P falciparum* only infections (appendix pp 30–58), seven were newly acquired (appendix pp 52–58), seven cleared during the study (appendix pp 40–46), ten were chronic oscillating infections (appendix pp 30–39), and five could not be determined because of the low number of positive samples (appendix pp 47–51). Infection profiles for individuals who had both *P falciparum* and non*-P falciparum* signals over the course of the study (n=20) were difficult to classify definitively without additional sequencing. 15 of these 20 infections were chronic oscillating infections (appendix pp 59–73). Four of these 15 individuals appeared to have chronic non*-P falciparum* infections during the collection period as well as a newly acquired *P falciparum* infection (appendix pp 70–73), and four individuals initially had mixed infections followed by *P falciparum* infections later (appendix pp 59, 64, 66, 68). The remaining seven infection profiles were difficult to classify further. For some participants, parasite density fluctuated in a classical sawtooth pattern associated with growth and cyclical sequestration of parasites, especially among *P falciparum* infections (figure 1A, B). However, there were some individuals where parasite densities fluctuated minimally each day, both with *P falciparum* and non-*P falciparum* infections (figure 1F, I).

Of 1291 positive DBSs, 70 DBSs (5·4%) among 21 individuals had more than 100 000 parasites per mL. However, only five of these 21 individuals developed symptoms of malaria during the study period and were found to be positive for *Plasmodium* using RDTs. Two of these individuals developed new *P falciparum* infections during the study, although the other three had parasite 18S rRNA detected at baseline and appeared to have chronic oscillating infections before testing RDT-positive.

**Figure 2:**
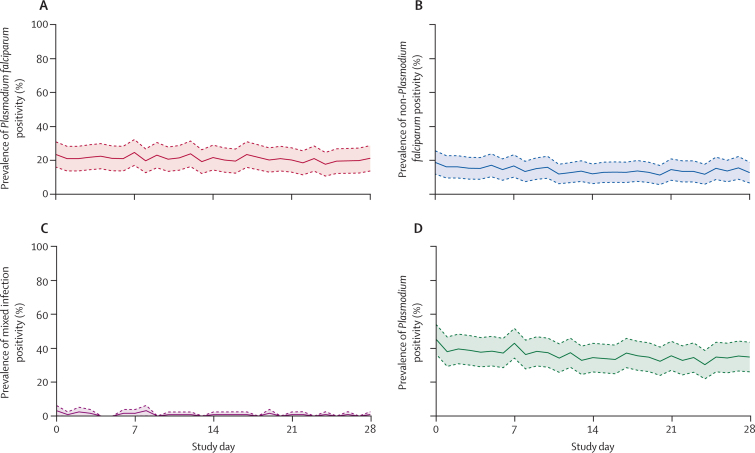
*Plasmodium* prevalence on each day of sampling for 128 study participants *Plasmodium* prevalence (solid line) and 95% CI (dashed lines and shaded area). (A) *Plasmodium falciparum* infections only. (B) Non-*P falciparum* infections only. (C) Mixed infections. (D) Any *Plasmodium* infection.

The prevalence of *Plasmodium* infections ranged from a high of 45·3% (95% CI 36·6–54·1) at baseline to a low of 30·3% (21·9–38·6) on day 24 of the study (figure 2). Variation was similar for *P falciparum* and non-*P falciparum* infections. 56 (56·0%) of 100 adults and 21 (75·0%) of 28 children had at least one positive DBS. Among children with at least one positive DBS, the median number of days negative for *Plasmodium* was 1·5 (IQR 0·0–11·8) for male participants and 0·5 (0·0–4·3) for female participants. Among adults, the median number of negative days was 0 for both male participants (IQR 0·0–7·5) and female participants (0·0–6·0). Of 63 individuals with a positive sample during the first week, five (7·9% [95% CI 2·6–17·6]) were negative on the first day of sampling.

Overall, there was 90% (95% CI 89–91) probability of participants having the same test result from one day to the next. Among individuals with a positive DBS on a given day, the probability of being negative the following day ranged from a low of 0% (0–9) to a high of 8% (7–21). Among individuals with a negative DBS on a given day, the probability of being positive the following day ranged from a low of 0% (0–5) to a high of 8% (5–19).

The proportion of infections that would have been detected with less frequent sampling was calculated for 106 individuals with complete DBS data. Among them, 64 (60·4%) had at least one positive DBS within the 29 day study period and were considered infected. Of those, 39 (60·9%) had at least one *P falciparum-*positive DBS and 41 (64·1%) had at least one non-*P falciparum*-positive DBS (table 2). With every other day sampling, 37 (94·9%) of 39 *P falciparum* and 35 (85·4%) of 41 non-*P falciparum* infections would have been detected. With weekly or every other week sampling, 35 (89·7%) *P falciparum* infections would have been detected. Sampling weekly would have detected 31 (75·6%) non-*P falciparum* infections, and sampling every other week would have detected 28 (68·2%) non-*P falciparum* infections. Sampling only at the beginning and end of the study would have detected 34 (87·2%) *P falciparum* and 27 (65·9%) non-*P falciparum* infections.

**Figure 3:**
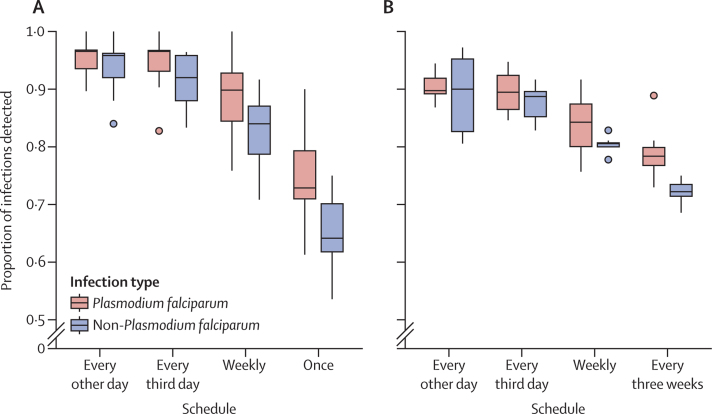
Proportion of infections detected with reduced sampling schedules in rolling windows The numerator of each proportion is the number of infections detected in each window with the indicated subset of samples, and the denominator is the number of infections detected in each window by daily sampling. (A) Infection defined within windows of 7 days (n=22). (B) Infection defined within windows of 21 days (n=8). Reduced schedules are every other day, every third day, weekly, every three weeks (21 day period only) or once (7 day period only).

When infection was defined within a rolling 21 day period, the number of participants with one or more positive DBSs ranged between 59 (55·7%) and 62 (58·5%) with daily sampling, depending on start day. Sampling every other day detected a median of 90% (IQR 89–92) of *P falciparum* infections and 90% (83–95) of non-*P falciparum* infections, and sampling every 3 days detected a median of 89% (IQR 86–92) of *P falciparum* and 89% (85–90) of non-*P falciparum* infections. The median proportion of infections decreased when sampling was done weekly or only at the beginning and end of the detection period (figure 3).

Comparable results were seen when infections were defined within a rolling 7 day period. The number of participants with one or more positive DBS within the sampled 7 day periods ranged between 44 (41·5%) and 53 (50·0%) with daily sampling. Sampling every other day detected a median of 97% (94–97) of *P falciparum* infections and 96% (92–96) of non-*P falciparum* infections, although sampling every 3 days detected a median of 97% (93–97) of *P falciparum* and 92% (88–96%) of non-*P falciparum* infections. The median proportion of infections decreased with more infrequent sampling schemes (figure 3).

## Discussion

We analysed the daily dynamics of low-density, asymptomatic *Plasmodium* infections over a 29-day period in adults and older children in a high transmission area. About a third of all DBSs collected were positive for *Plasmodium* and 60% of all participants had a *Plasmodium* infection identified during the study. With highly sensitive diagnostics and a sampling method that allowed for larger sample sizes and more frequent testing, we observed more variation in daily species composition and parasite dynamics than previously reported.^1^^,^^3^^,^^4^^,^^7^^,^^8^

Our results highlight the pitfalls of single timepoint measurements for *Plasmodium* infection status, especially in areas with high prevalence of low-density infections. Although 60% of the study population in this study had a positive DBS at some point during the month-long study period, less than half were positive at baseline and daily prevalence fluctuated with a low of roughly 30%. Parasite sequestration was evident in most *P falciparum* cases, with densities often dropping below the limit of detection on several days during follow-up. This sequestration was demonstrated in two previous studies of asymptomatic infection in endemic populations that used blood smears^1^ or less analytically sensitive PCR.^2^ Additionally, the effect of endosplenic residence of parasites is beginning to be understood and might contribute to low densities of peripheral parasites in our study.^15^ When comparing the infections detected with daily sampling to those identified with less frequent sampling, we found the proportion of all infections detected in a 21 day period was similar between daily, every other day, and every third day sampling, and then became less reliable with weekly or study start and end sampling, demonstrating that, even with highly sensitive diagnostics, some low-density infections would still go undetected with infrequent or single timepoint testing. Even more infections would be missed if less analytically sensitive molecular tests were used. Missed infection might be especially problematic in clinical trials that rely on baseline sampling to rule out pre-existing infection in the study population. In our study, five individuals had a newly detected infection within the first 5 days of the study, suggesting that they had the infection at baseline but the parasites had not yet emerged from the liver stage. When we evaluated the effect of less frequent sampling on detecting infection during a 7 day period, which is most relevant to trials, the median proportion of infections detected was lower when sampling was done on weekly or single-day schedules (ie, just once) than with more frequent sampling.

Although cyclical sequestration was observed in many samples, not all trajectories demonstrated a sawtooth pattern of sequestration. For example, one adult female participant (figure 1F) had a chronic *P falciparum* infection during the study period, with very little variation in parasite density. Samples dominated by gametocytes could be one major reason for a *P falciparum* parasite density with no apparent sequestration pattern. Unlike asexual parasites, gametocytes do not sequester and can remain in the blood for at least 3 weeks.^16^ Many infections that did not show a sawtooth pattern in our study were those that were slowly clearing, which is supportive of slowly clearing gametocytaemia in these participants. Another factor that could affect the daily pattern is the time of day the sample was collected. A limitation of this study was that participants were not asked to take their sample at the same time each day. Since parasite densities have been shown to vary by more than 100-fold within a 6 h period,^1^ it is possible that some of the dynamics of infection were not observed because DBSs might have been collected at different parts of the day. In addition, individuals might have multiple infections with different synchronicities. In areas with high transmission, such as the study area, individuals often have polyclonal infections within a single *Plasmodium* species. Repeat sampling has been shown to be necessary for detecting multiclonal infections, with more than a quarter of additional clones not appearing on the first day of sampling.^8^ Densities of polyclonal infections have been shown to peak at different timepoints during a 3-day period,^3^ resulting in overall densities that have little fluctuation, even though individual genotypes are sequestering. Therefore, if an individual is infected with multiple clones with asynchronous amplification, overall densities might appear unchanging despite more complex underlying dynamics. Additional explanations might include sickle cell trait (HbAS), which has been shown to protect against high parasite densities^17^ and might affect dynamics as well as increase the likelihood of gametocytaemia.^18^ Another limitation of the current study is that we did not quantify gametocytes versus asexual stages, multiplicity of infection, or HbAS; such factors could be assessed in future studies.

Our study showed there is a high prevalence of low-density non-*P falciparum* infections in this population, as seen in other studies.^19^ A limitation of our study was that, in mixed infections where *P falciparum* predominated, the qRT-PCR assay that we used could not specify whether a non-*P falciparum* species was also present. It was likely that some of the *P falciparum* infections identified here were actually mixed infections. Additionally, due to the low-parasite densities and small blood volumes in the current study, it was not possible to perform species identification on all non-*P falciparum* samples.

Various longitudinal trajectories observed in our study align with the theoretical trajectories hypothesised by Drakeley and colleagues.^20^ Most infections in our study were chronic (over the 29 day period) and did not clear or become symptomatic; therefore, we could not further categorise these infections into specific theoretical trajectories proposed. Additionally, our study was limited by the study population and transmission setting. Younger children (younger than 8 years) were not included in the study and dynamics in this population might be different than in older children. Information on less frequent sampling derived from this study might be useful for designing future studies in younger children, where additional ethical consideration must be given regarding repeat sampling. Additionally, this study was conducted in an area of high transmission during a period of peak transmission.^21^ We might expect fewer asymptomatic infections and different dynamics in lower transmission areas or during different transmission periods.^22^ The methods used and data generated from our study can be leveraged to design longer-term studies in different populations, which are needed to better differentiate the proportions of various potential outcomes of low-density infections in endemic populations.

This study shows that there is a large depth and breadth of infection dynamics among asymptomatic, low-density *Plasmodium* infections in an endemic area. The findings indicate that more frequent sampling might be needed to better understand the different types of trajectories in endemic areas and the frequency at which these infections occur, and to assess the effect of these dynamics on transmission and elimination efforts.

## Data sharing

De-identified parasite dynamic data that includes age group, sex, study day, pan-*Plasmodium* and *Plasmodium falciparum* densities, and dried blood sample infection type classification are publicly available via the University of Washington ResearchWorks (http://hdl.handle.net/1773/50982), along with the study protocol (http://hdl.handle.net/1773/50981). Reasonable access to additional de-identified data and data dictionaries for research purposes can be obtained by writing to Sean Murphy (murphysc@uw.edu).

## Declaration of interests

We declare no competing interests.
