# Supplementary material for: Assessing the daily natural history of asymptomatic Plasmodium infections in adults and older children in Katakwi, Uganda: a longitudinal cohort study
Source: Lancet Microbe. 2024 Jan;5(1):e72–80. doi: 10.1016/S2666-5247(23)00262-8 (PMC10790327; doi:10.1016/S2666-5247(23)00262-8)
Supplement: Supplementary appendix 1 [file mmc1.pdf]

Supplementary Appendix

Table of Contents

**Content**	**Page**
Supplementary Table 1. Subsets of the data used to simulate alternate testing schedules.	1
Plots of *Plasmodium* 18S rRNA biomarker log_{10} copies/mL for each study participant with qRT-PCR positivity	2-78

Supplementary Table 1. Subsets of the data used to simulate alternate testing schedules.

Windows were rolled in 1-day increments until the last day in the window coincided with the last day of the study (Day 28).

Window size	Schedule	First window	Last window	DBS per window
**29-day**	Q2D	0, 2, 4, … 28	NA	15
	Q7D	0, 7, 14, 21, 28	NA	5
	Q14D	0, 14, 28	NA	3
	Q28D	0, 28	NA	2
**21-day**	Q2D	0, 2, 4, … 20	7, 9, 11, … 27	11
	Q3D	0, 3, 6, … 21	7, 10, 13, … 28	8
	Q7D	0, 7, 14, 21	7, 14, 21, 28	4
	Q21D	0, 21	7, 28	2
**7-day**	Q2D	0, 2, 4, 6, 7	21, 23, 25, 27, 28	5
	Q3D	0, 3, 6, 7	21, 24, 27, 28	4
	Q7D	0, 7	21, 28	2
	Once	7	28	1

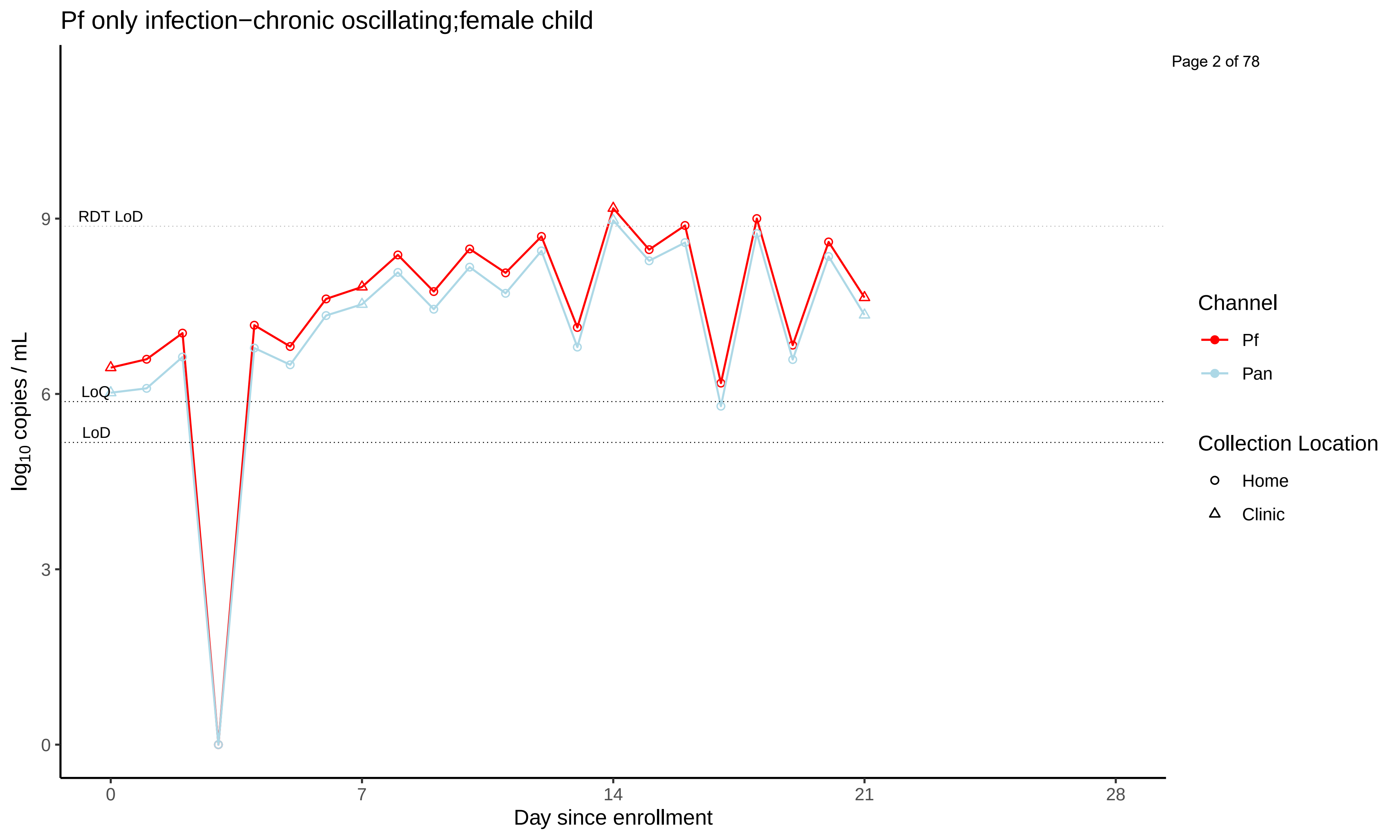
Pf only infection−chronic oscillating;female child
RDT LoD
LoQ
LoD
9
6
3
0
log10 copies / mL
0
7
14
21
28
Day since enrollment
Channel
Pf
Pan
Collection Location
Home
Clinic

Pf only infection−chronic oscillating;adult female

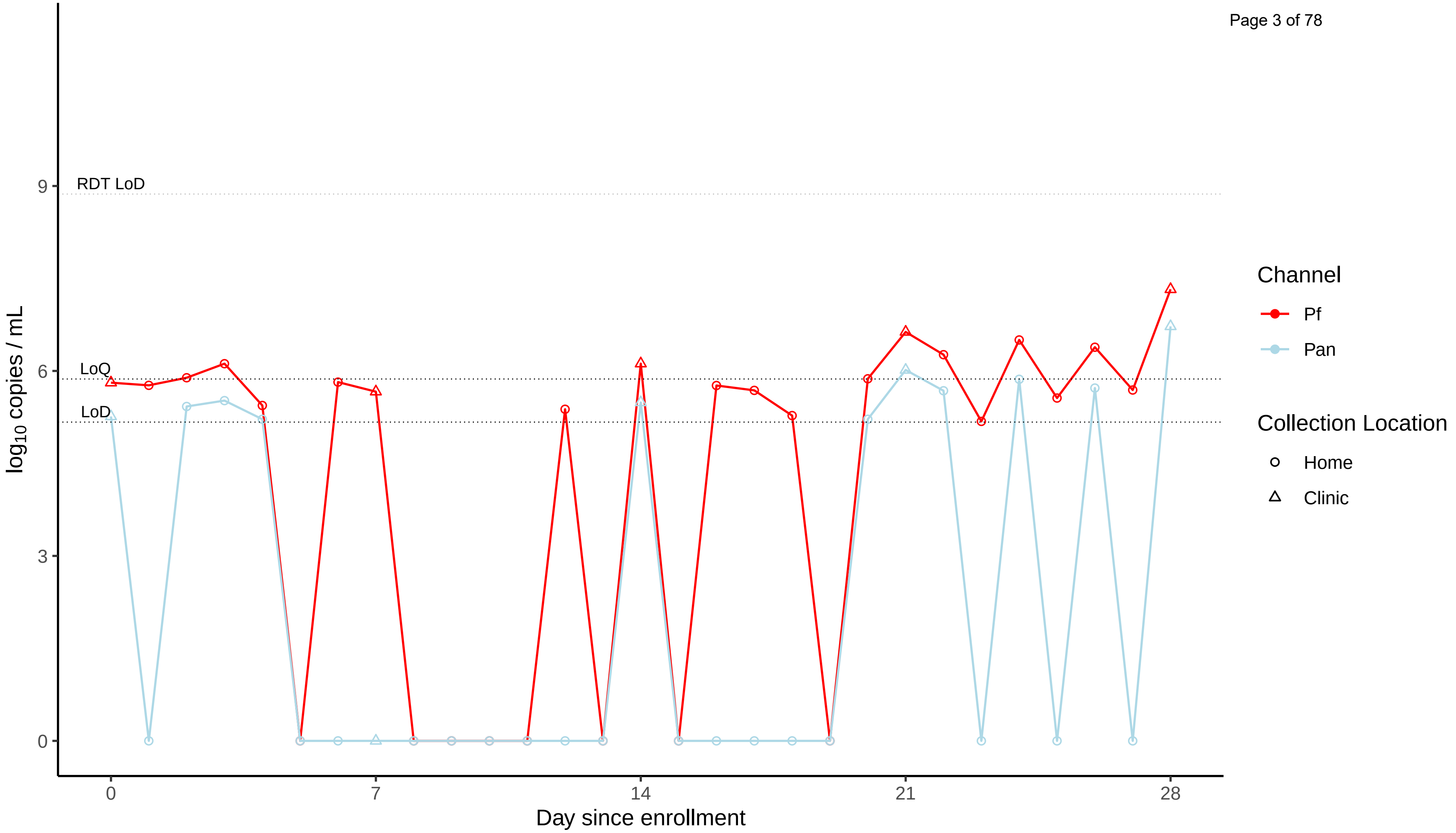

Pf only infection−chronic oscillating;adult female

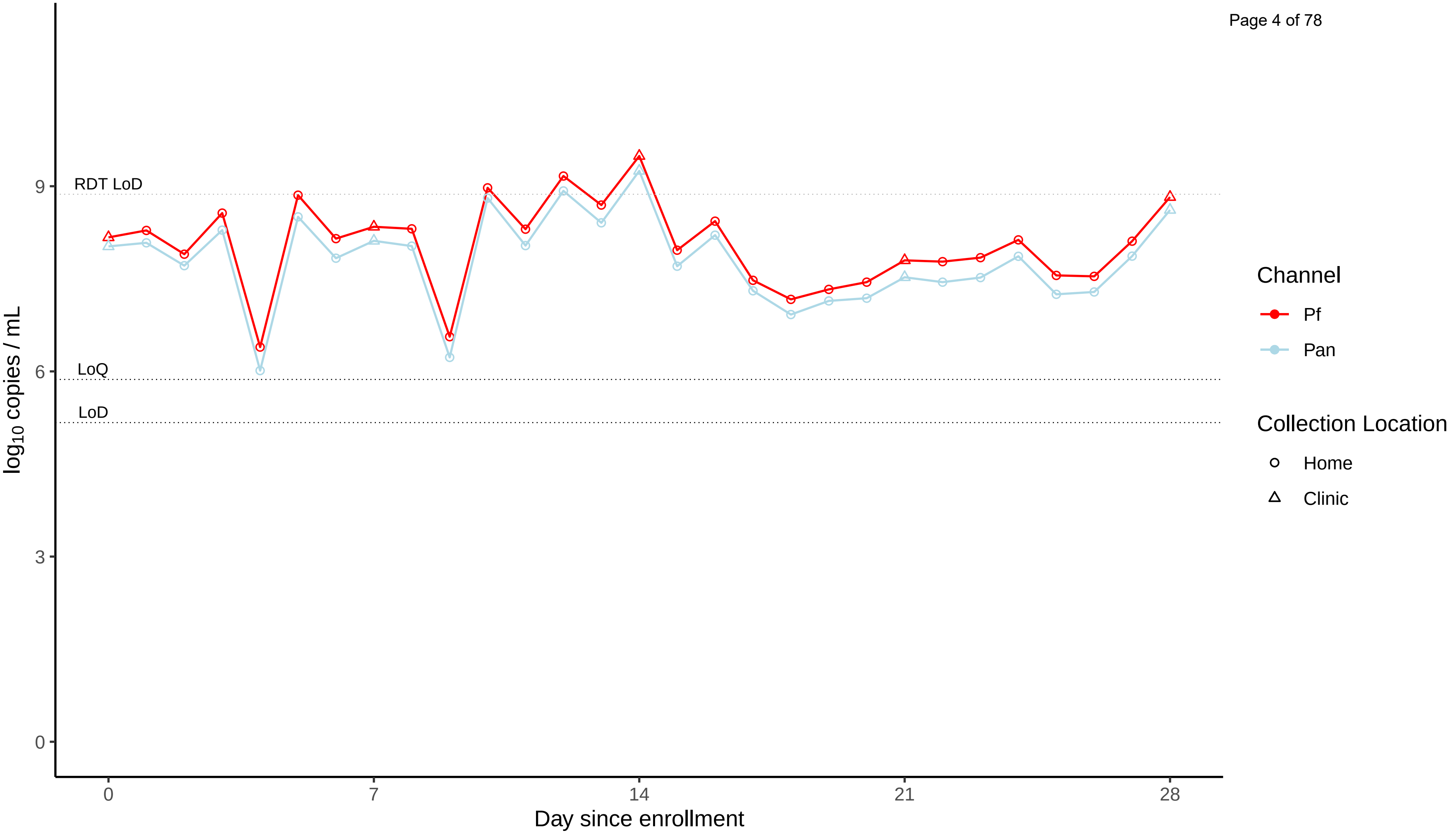

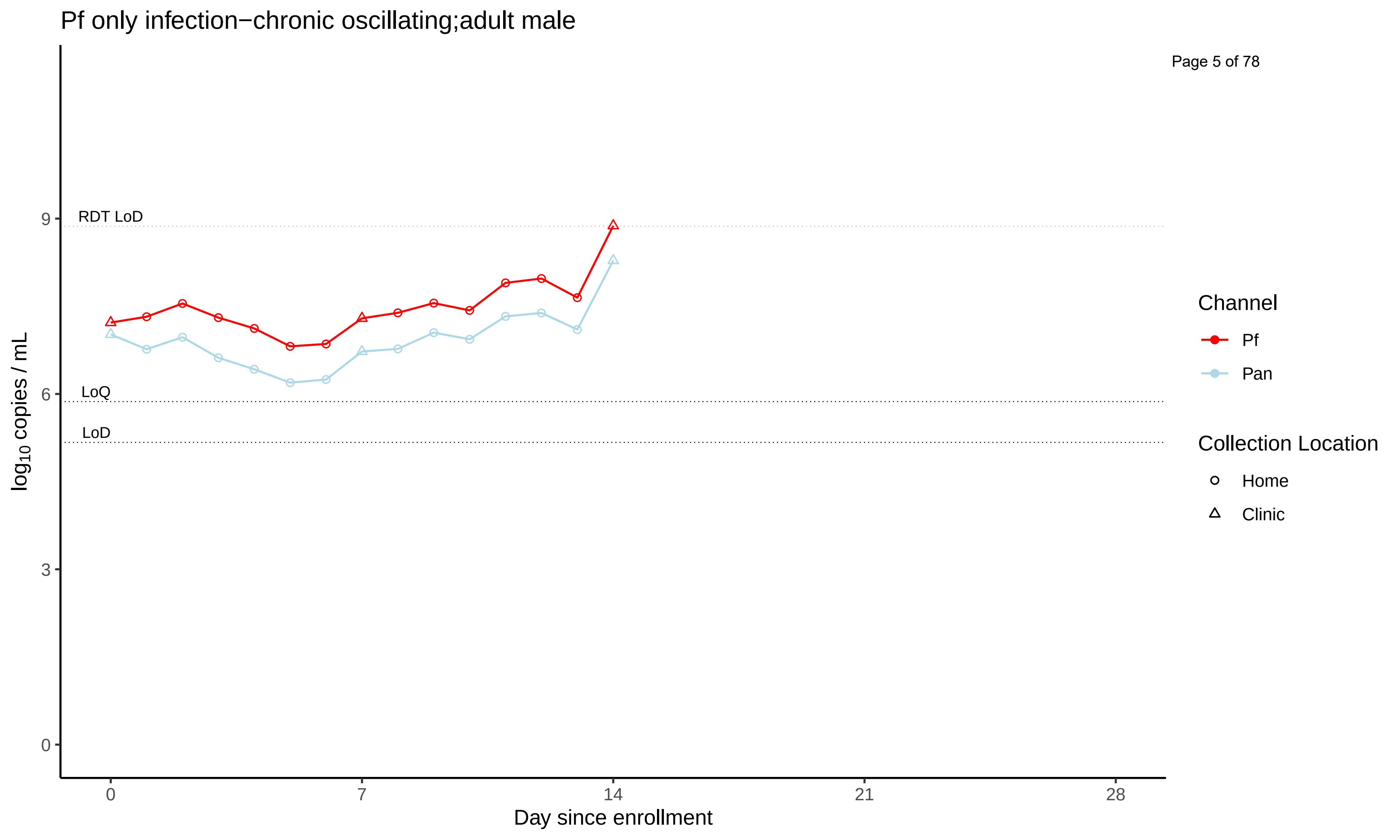

Pf only infection−chronic oscillating;adult male
Page 5 of 78
RDT LoD
LoQ
LoD
9
6
3
0
log10 copies / mL
0
7
14
21
28
Day since enrollment
Channel
Pf
Pan
Collection Location
Home
Clinic

Pf only infection−chronic oscillating;adult female

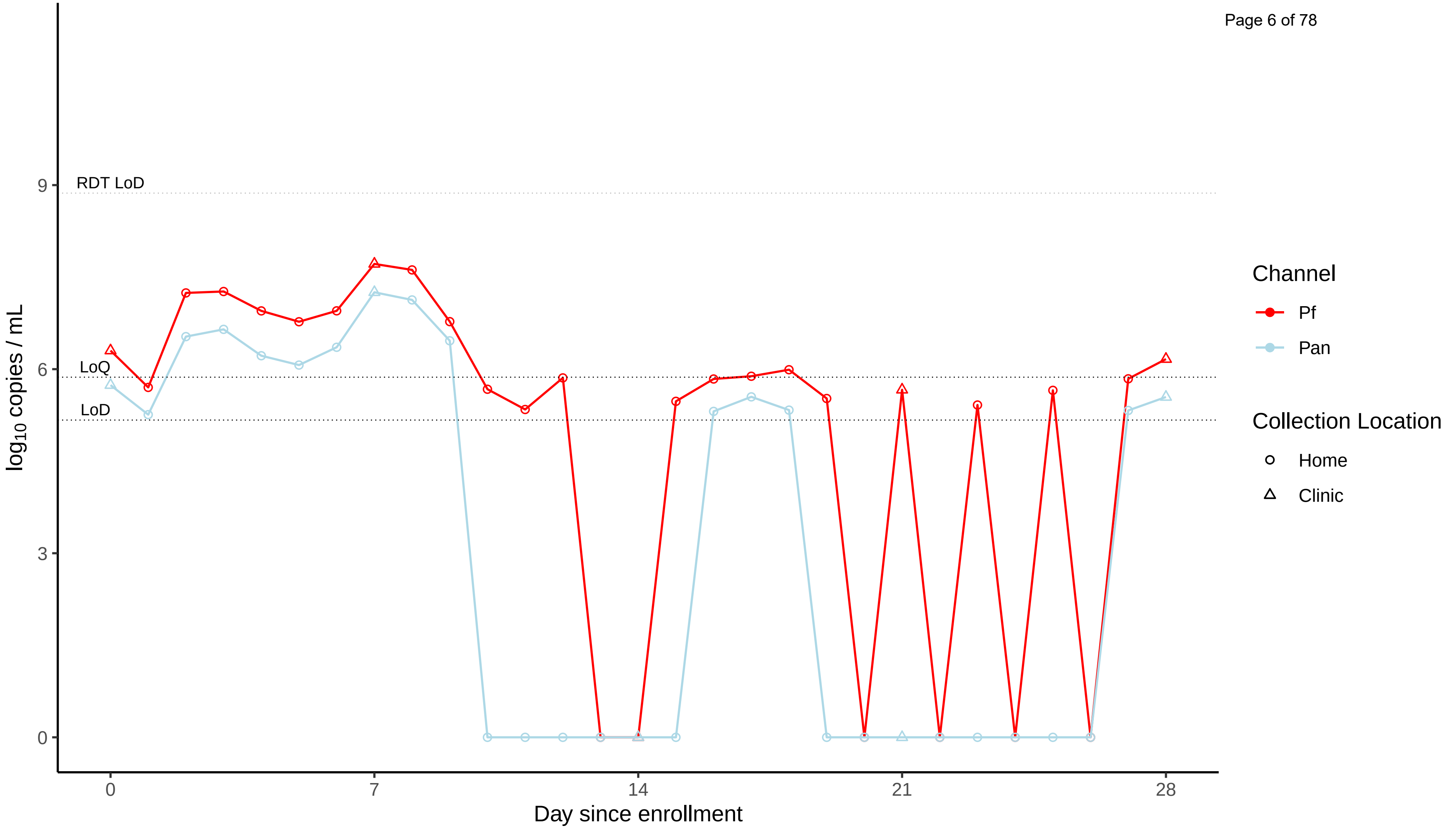

Pf only infection−chronic oscillating;male child

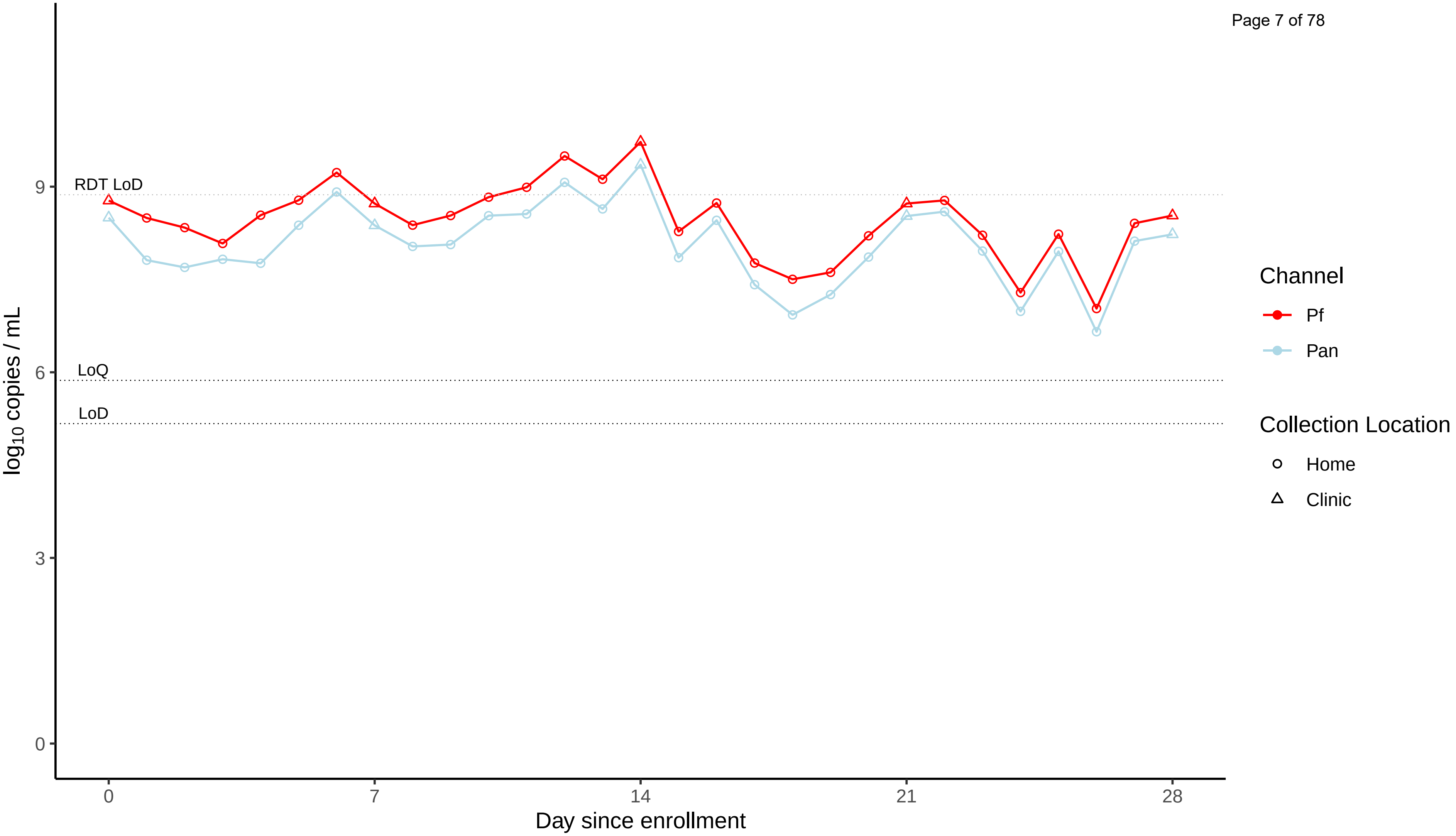

Pf only infection−chronic oscillating;adult male

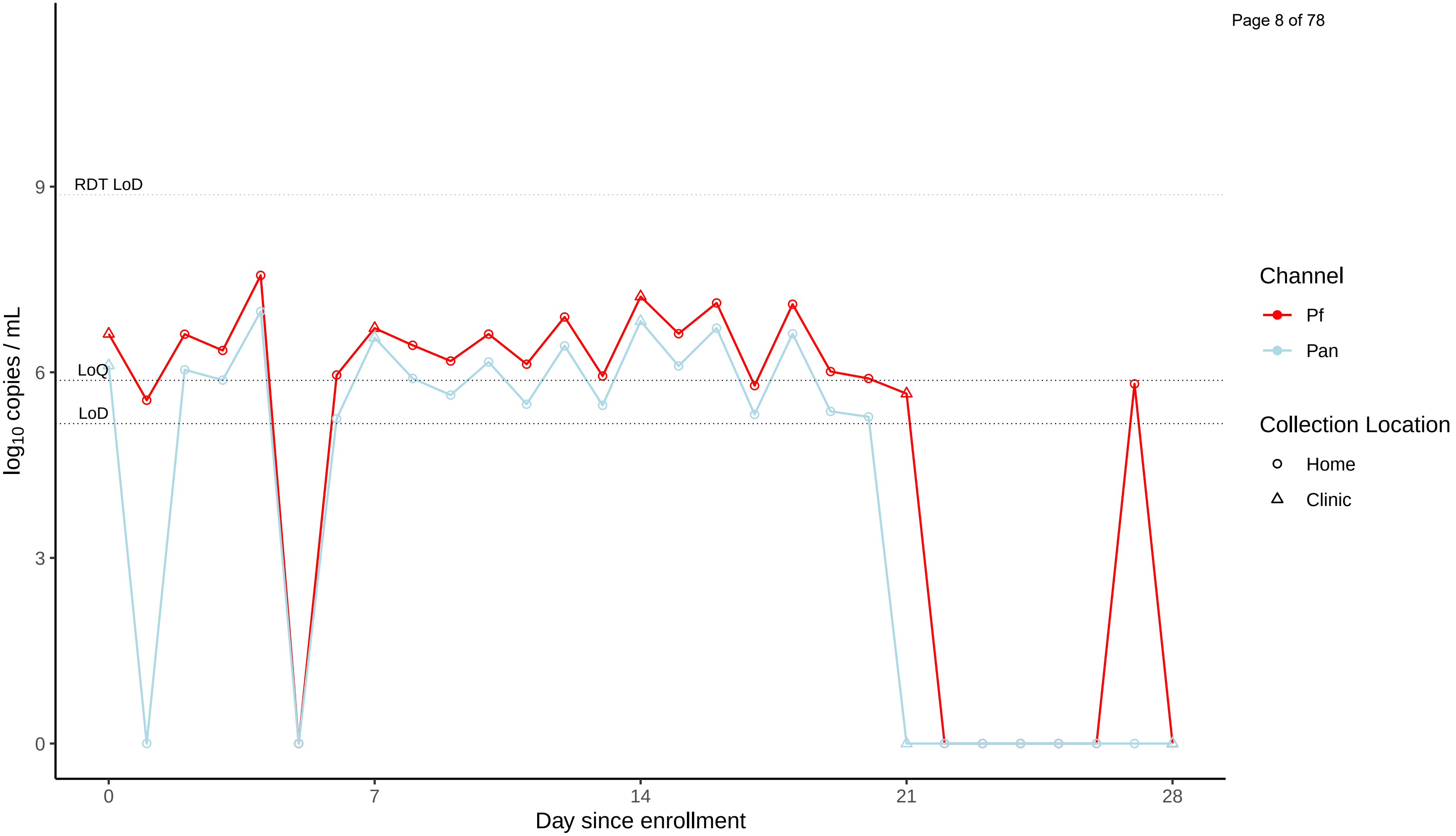

Pf only infection−chronic oscillating;female child

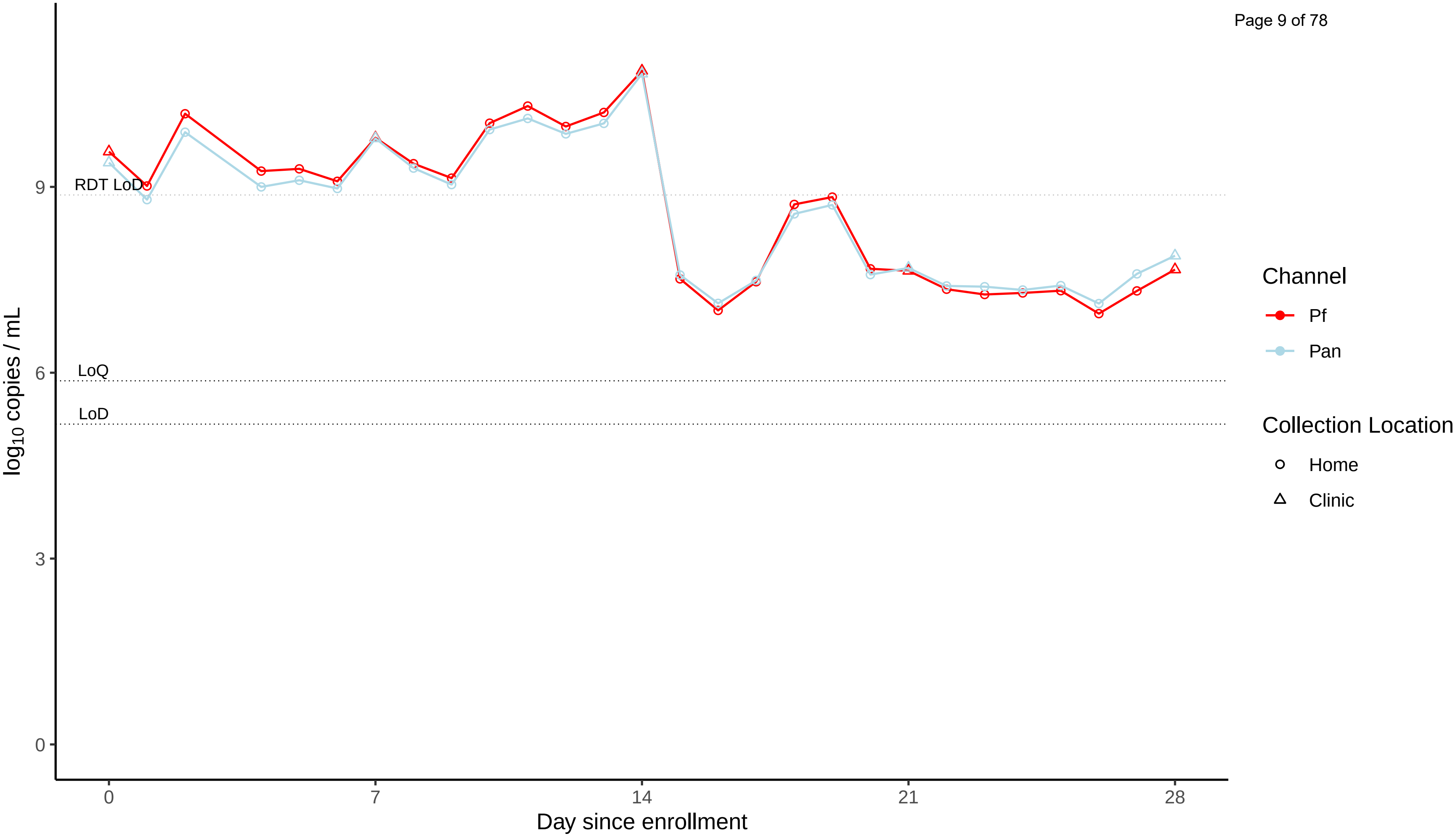

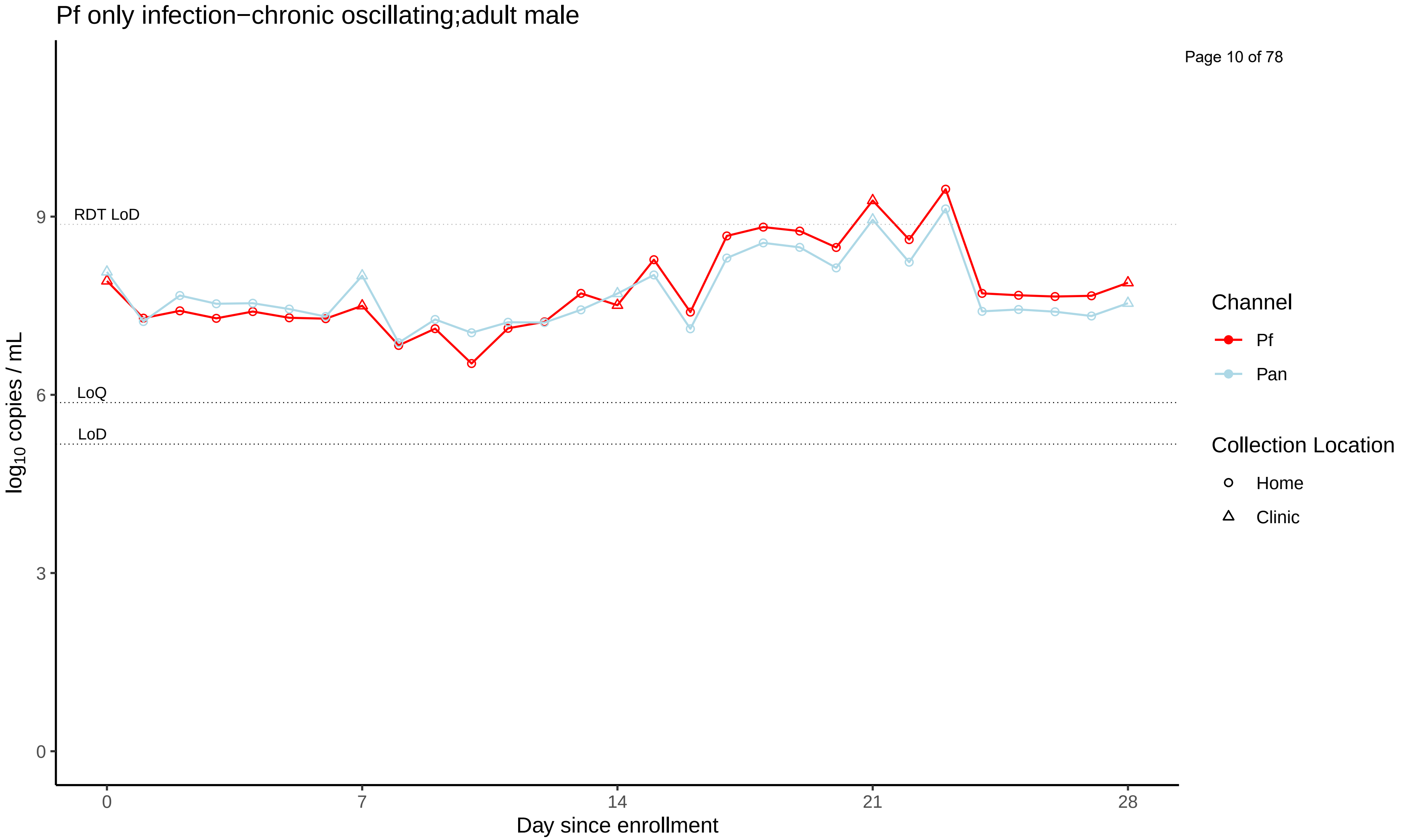

Pf only infection−chronic oscillating;adult male
RDT LoD
LoQ
LoD
9
6
3
0
log10 copies / mL
0
7
14
21
28
Day since enrollment
Channel
Pf
Pan
Collection Location
Home
Clinic

Pf only infection−chronic oscillating;adult female

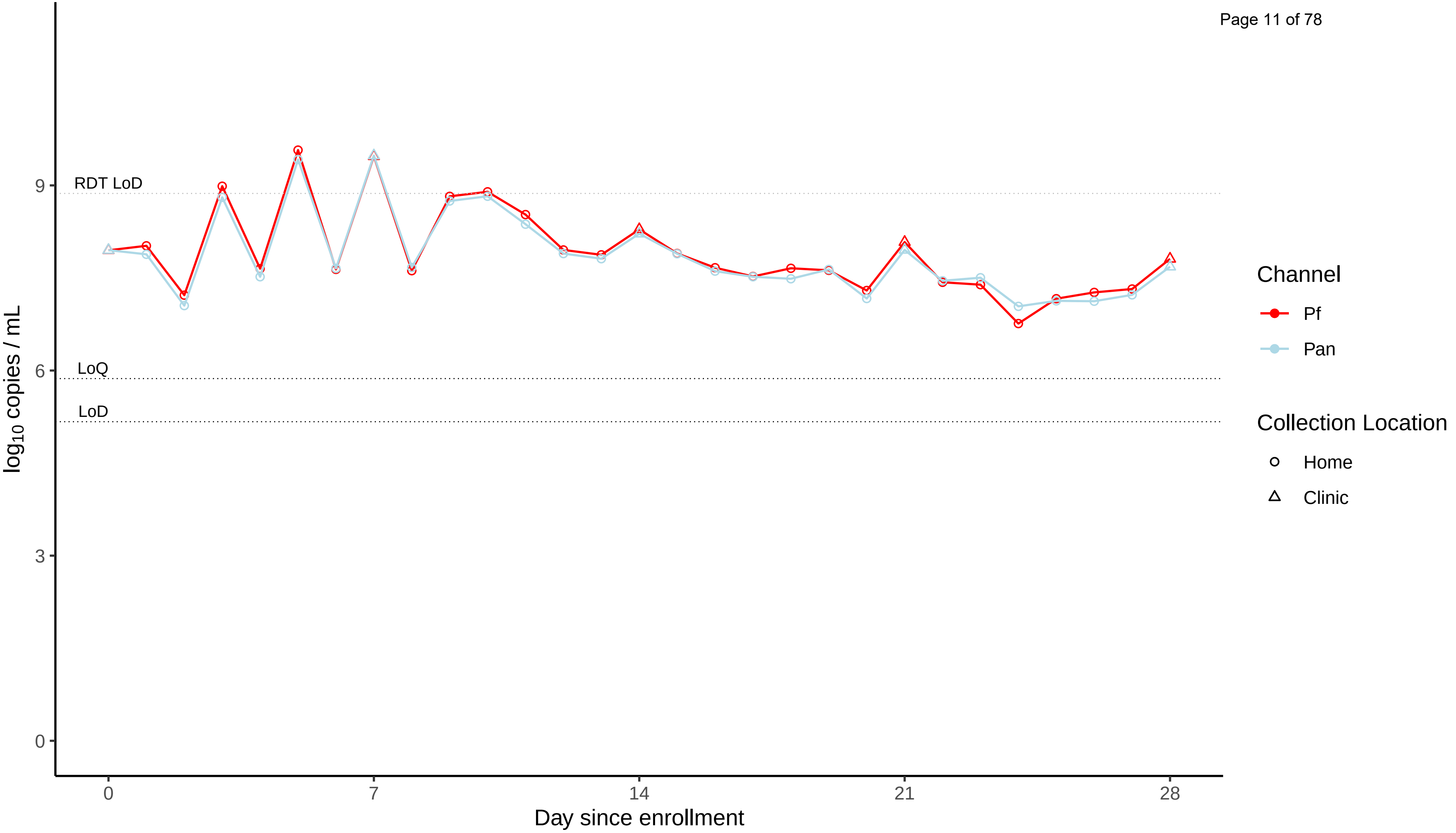

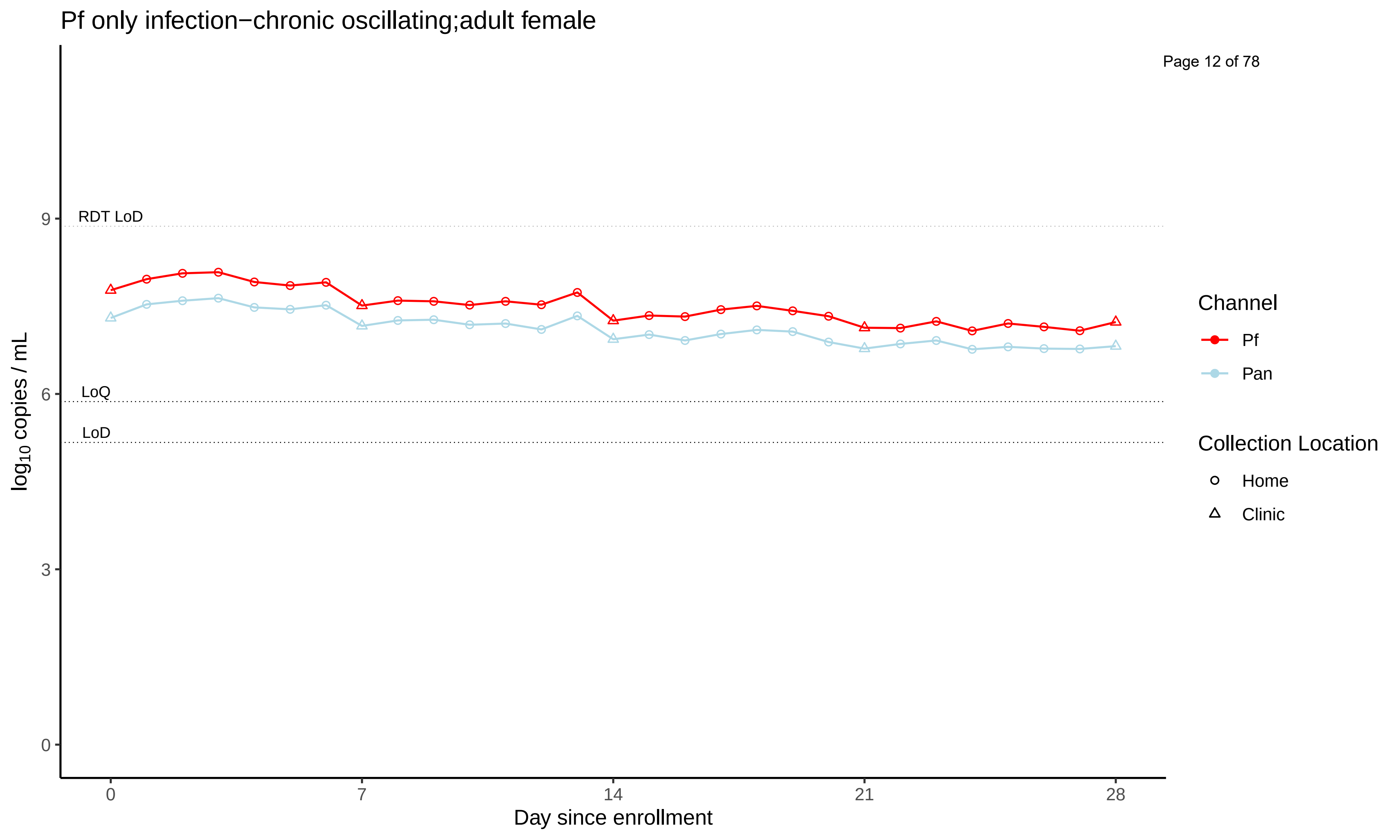
Pf only infection−chronic oscillating;adult female
RDT LoD
LoQ
LoD
9
6
3
0
$\log_{10}$ copies / mL
0
7
14
21
28
Day since enrollment
Channel
Pf
Pan
Collection Location
Home
Clinic

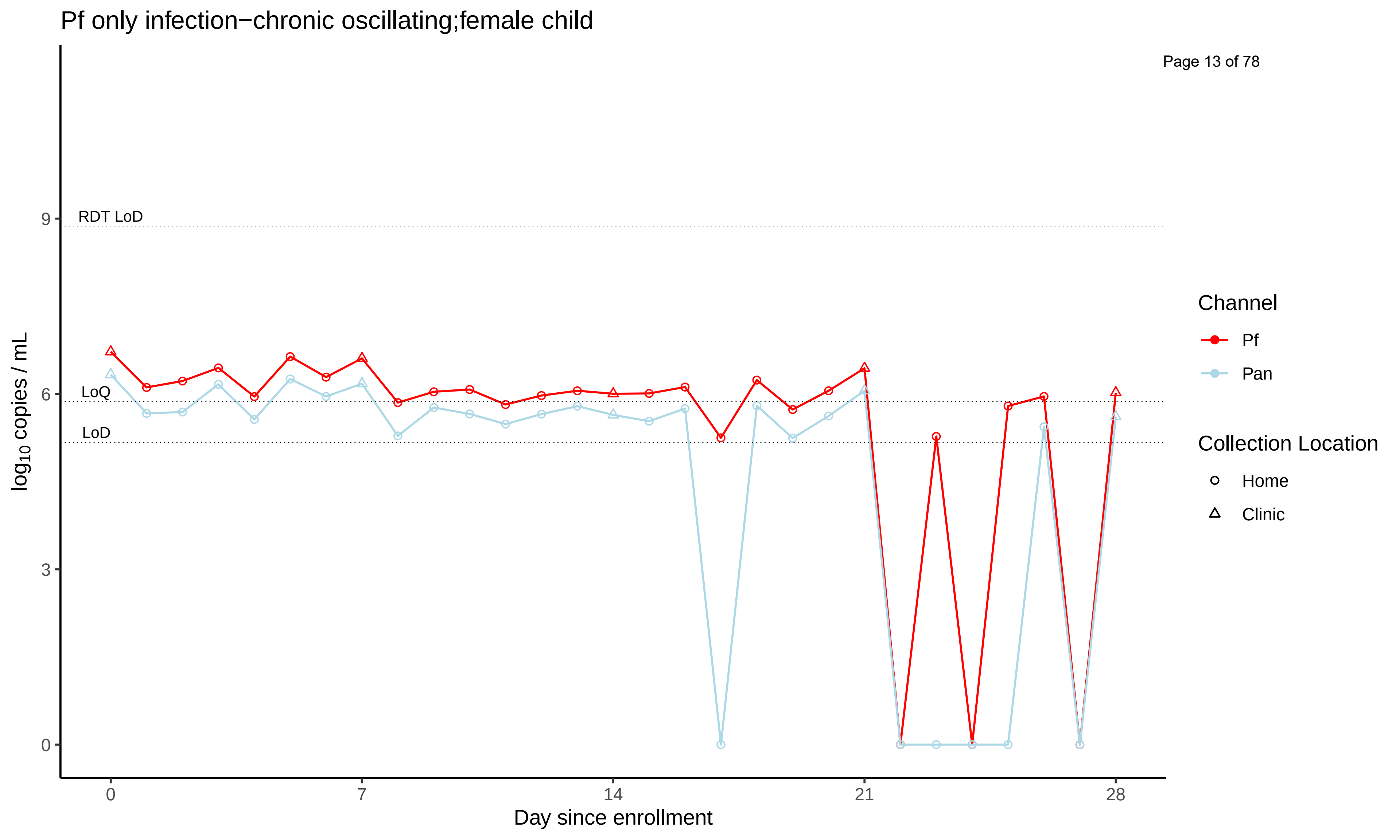
Pf only infection−chronic oscillating;female child
RDT LoD
LoQ
LoD
9
6
3
0
$\log_{10}$ copies / mL
0
7
14
21
28
Day since enrollment
Channel
Pf
Pan
Collection Location
Home
Clinic

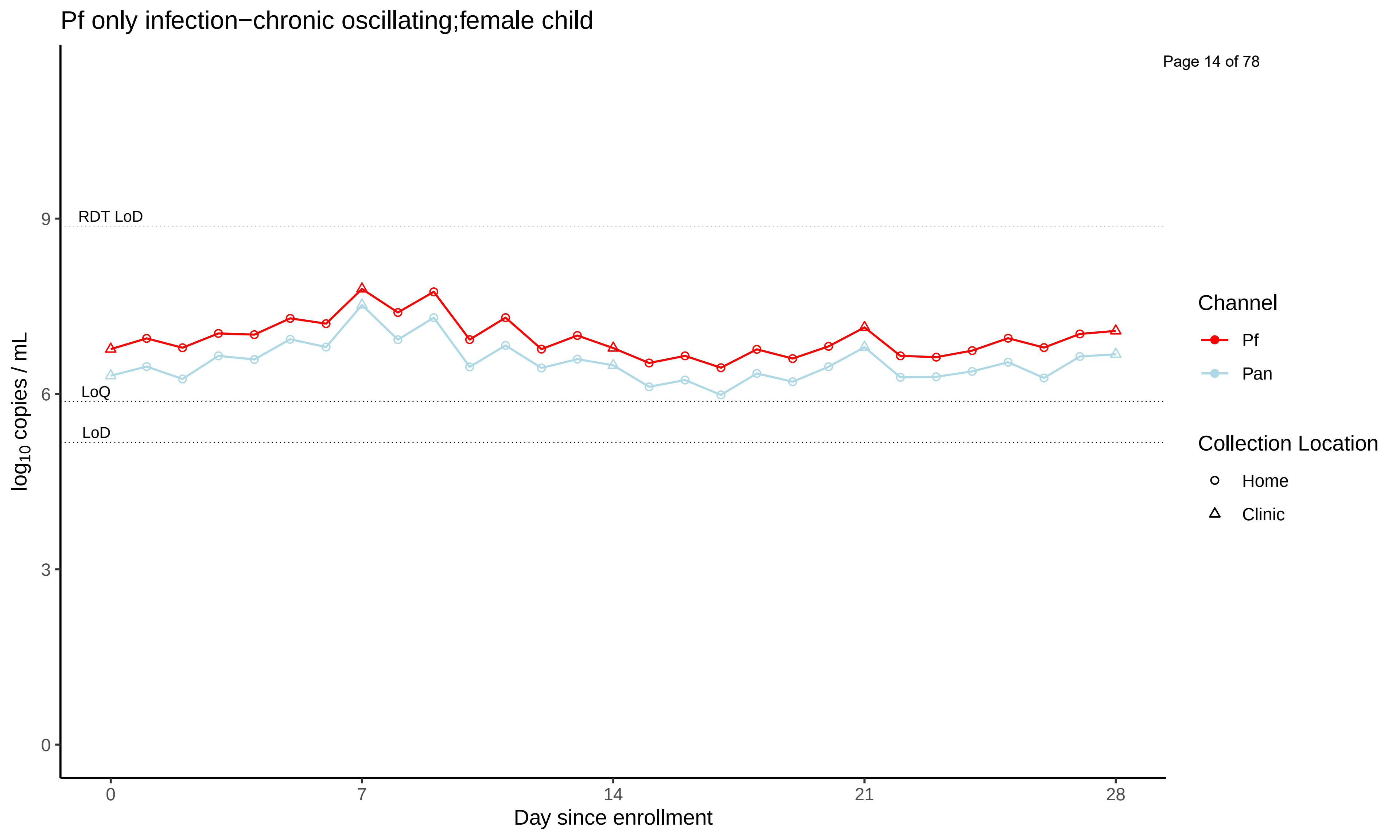
Pf only infection−chronic oscillating;female child
RDT LoD
LoQ
LoD
9
6
3
0
log10 copies / mL
0
7
14
21
28
Day since enrollment
Channel
Pf
Pan
Collection Location
Home
Clinic

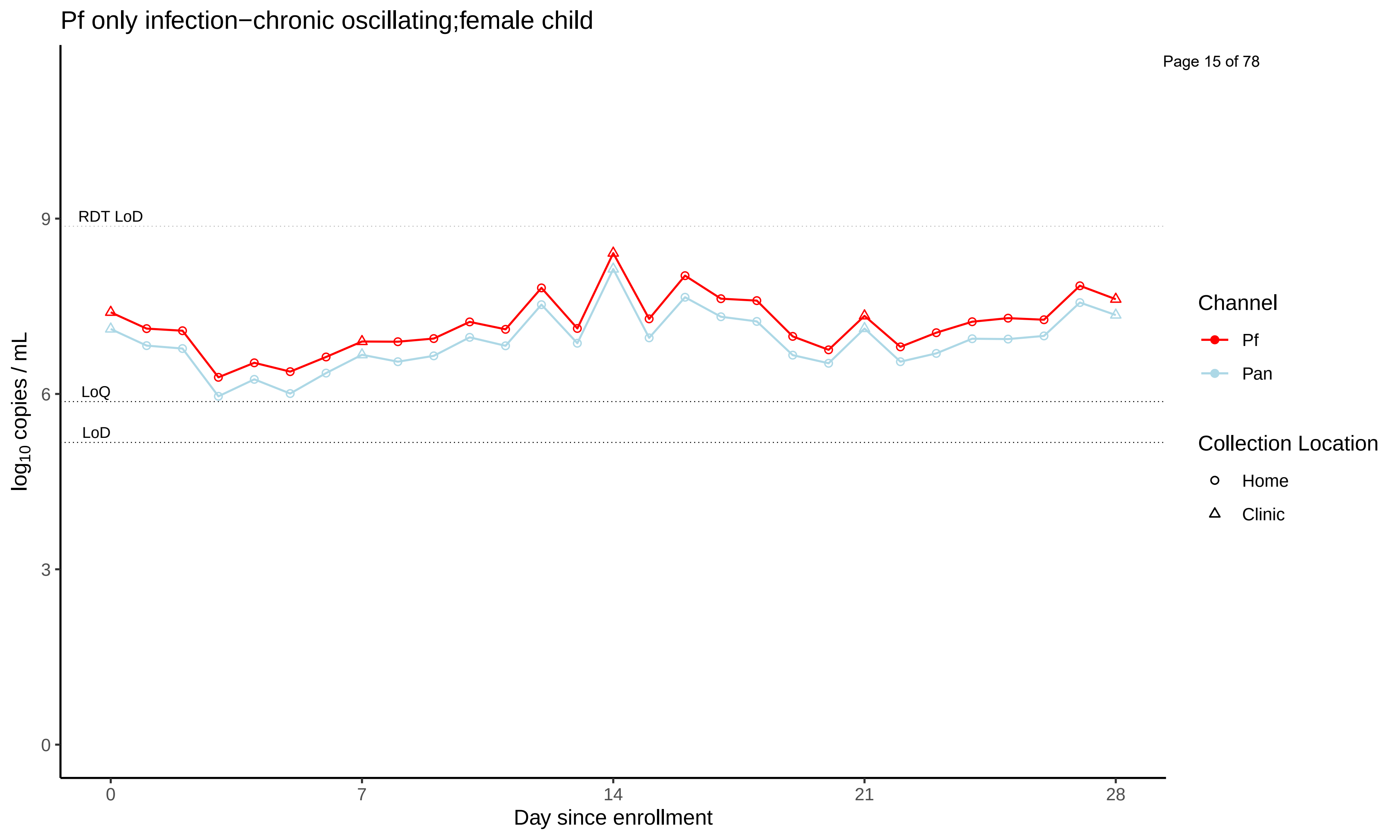
Pf only infection−chronic oscillating;female child
RDT LoD
LoQ
LoD
9
6
3
0
0
7
14
21
28
Day since enrollment
log10 copies / mL
Channel
Pf
Pan
Collection Location
Home
Clinic

Pf only infection−cleared;adult male

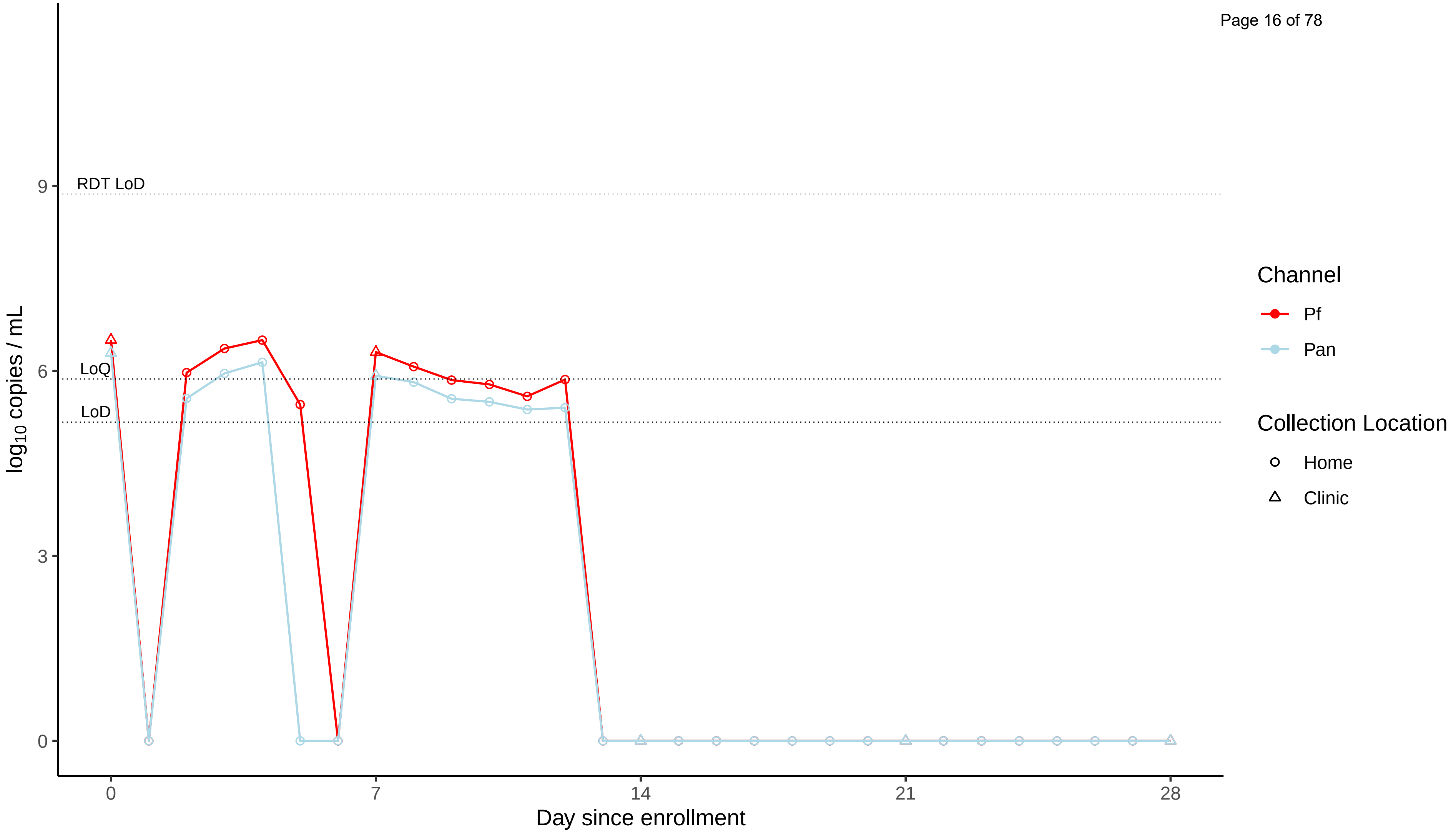

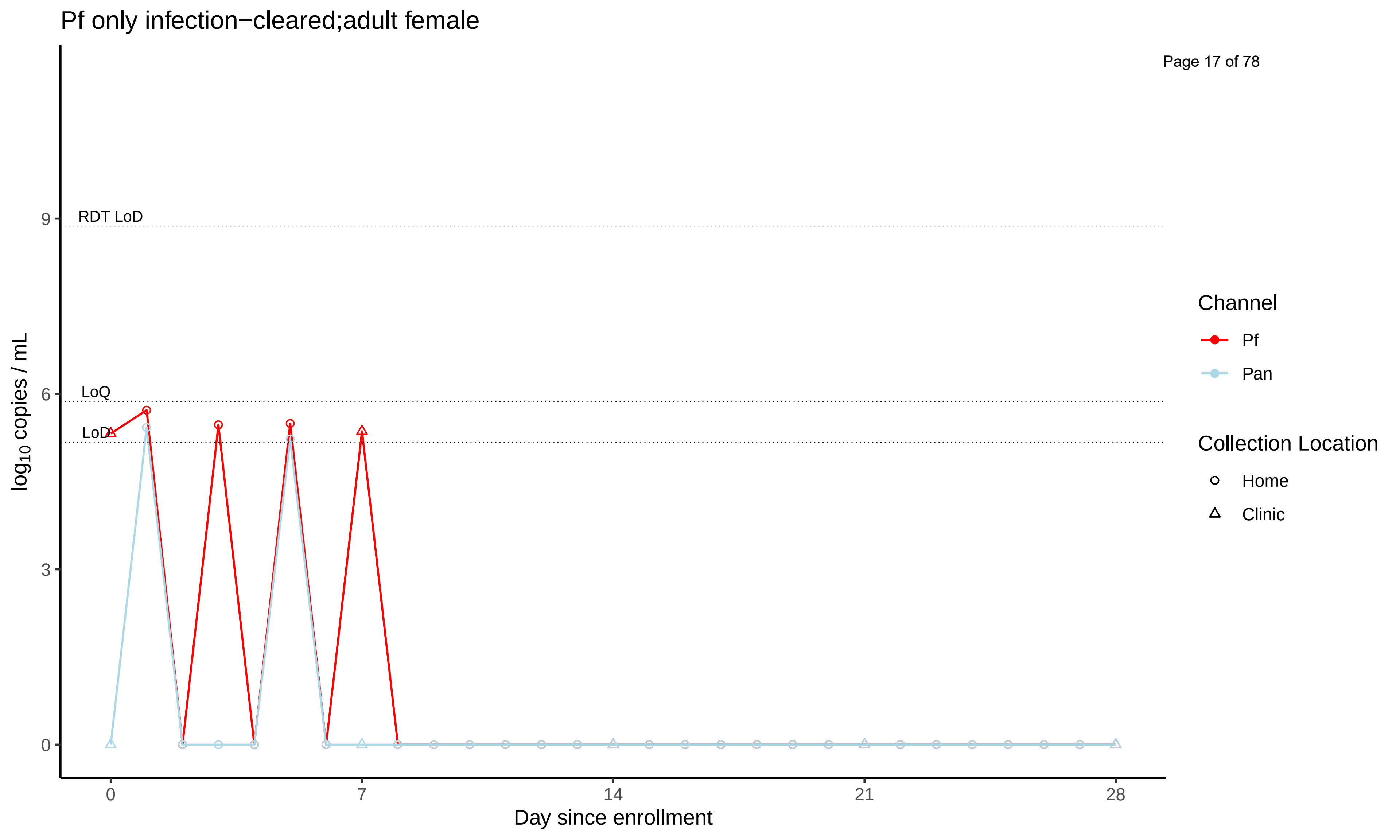
Pf only infection−cleared;adult female
RDT LoD
LoQ
LoD
9
6
3
0
0
7
14
21
28
log10 copies / mL
Day since enrollment
Channel
Pf
Pan
Collection Location
Home
Clinic

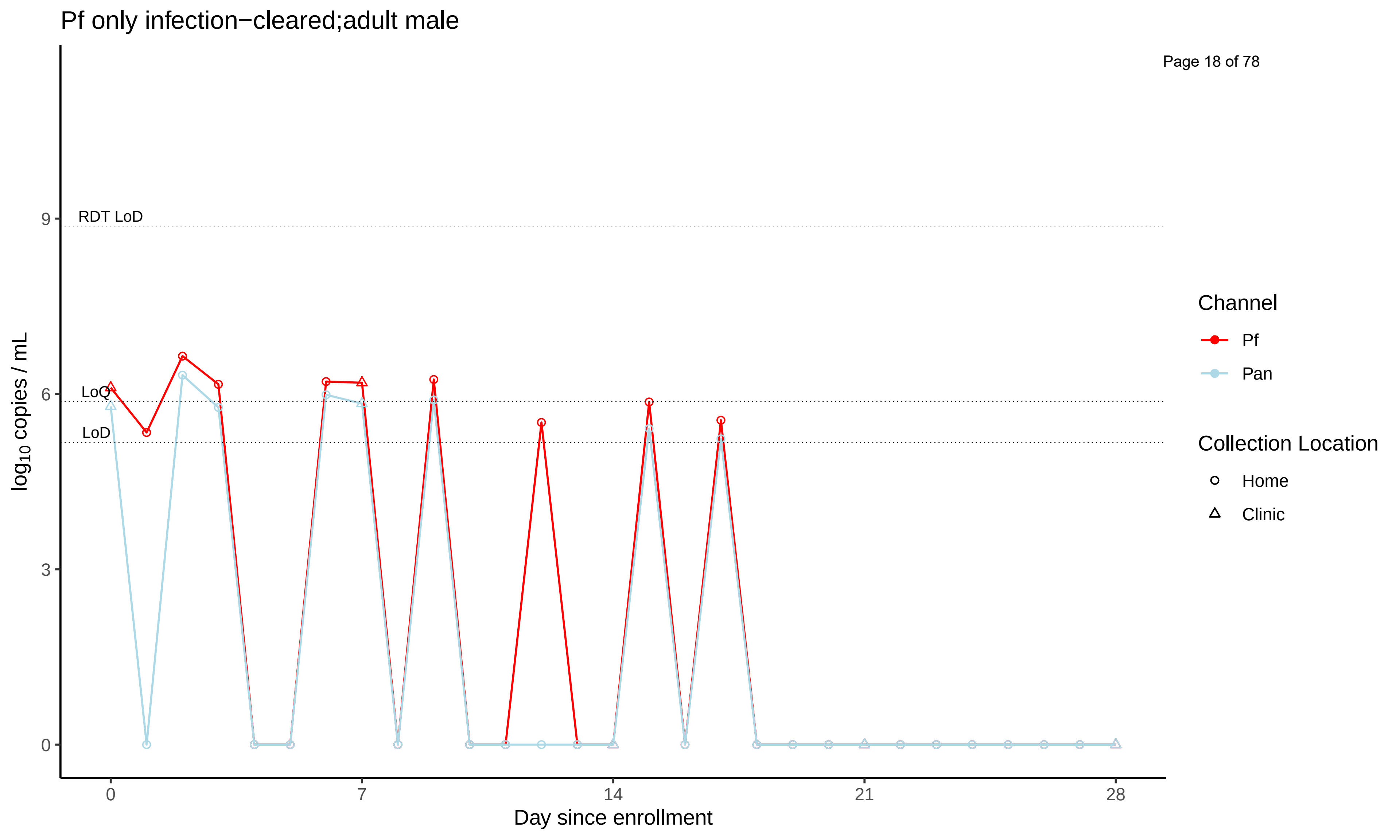

Pf only infection−cleared;adult male
Page 18 of 78
RDT LoD
LoQ
LoD
9
6
3
0
0
7
14
21
28
Day since enrollment
$\log_{10}$ copies / mL
Channel
Pf
Pan
Collection Location
Home
Clinic

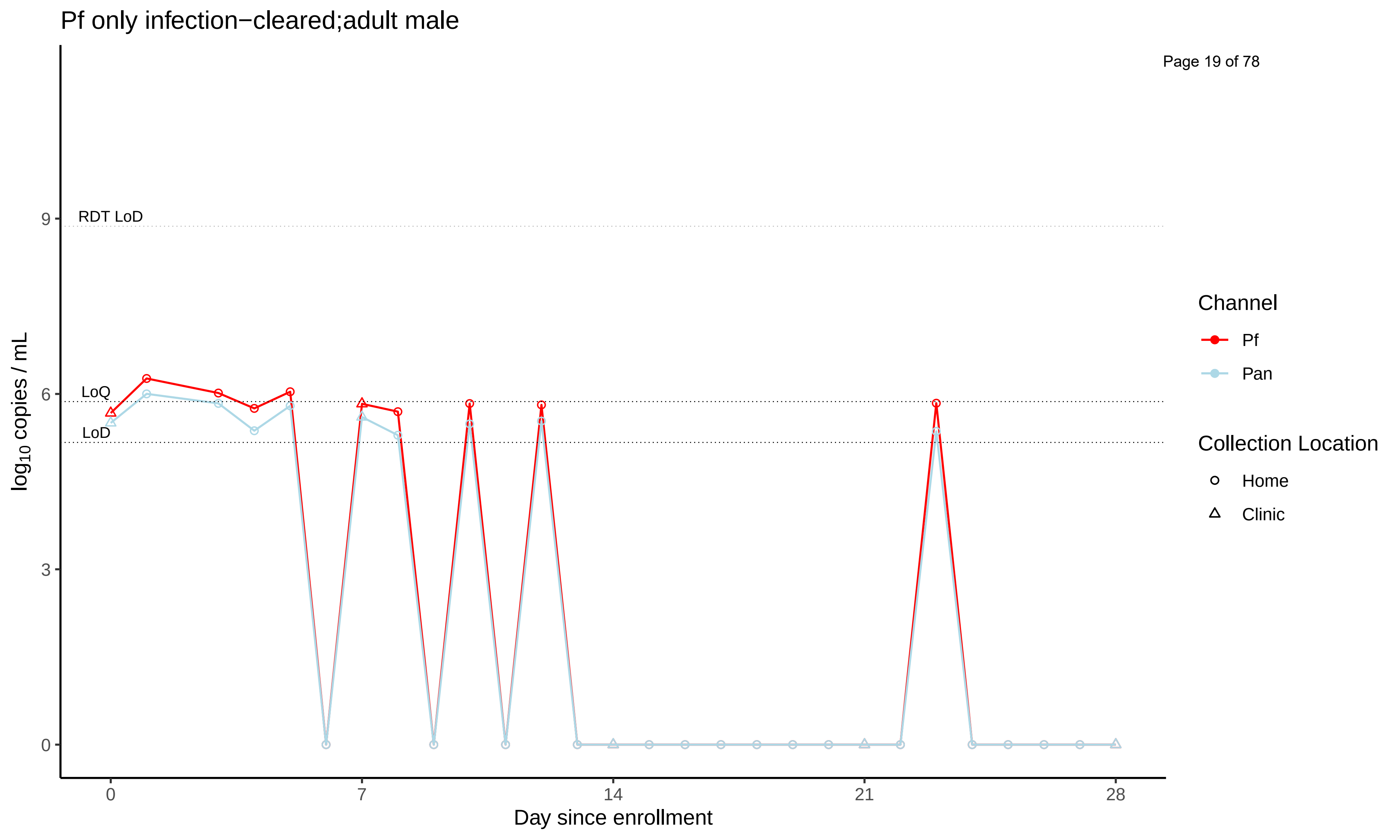
Pf only infection−cleared;adult male
RDT LoD
LoQ
LoD
9
6
3
0
log10 copies / mL
0
7
14
21
28
Day since enrollment
Channel
Pf
Pan
Collection Location
Home
Clinic

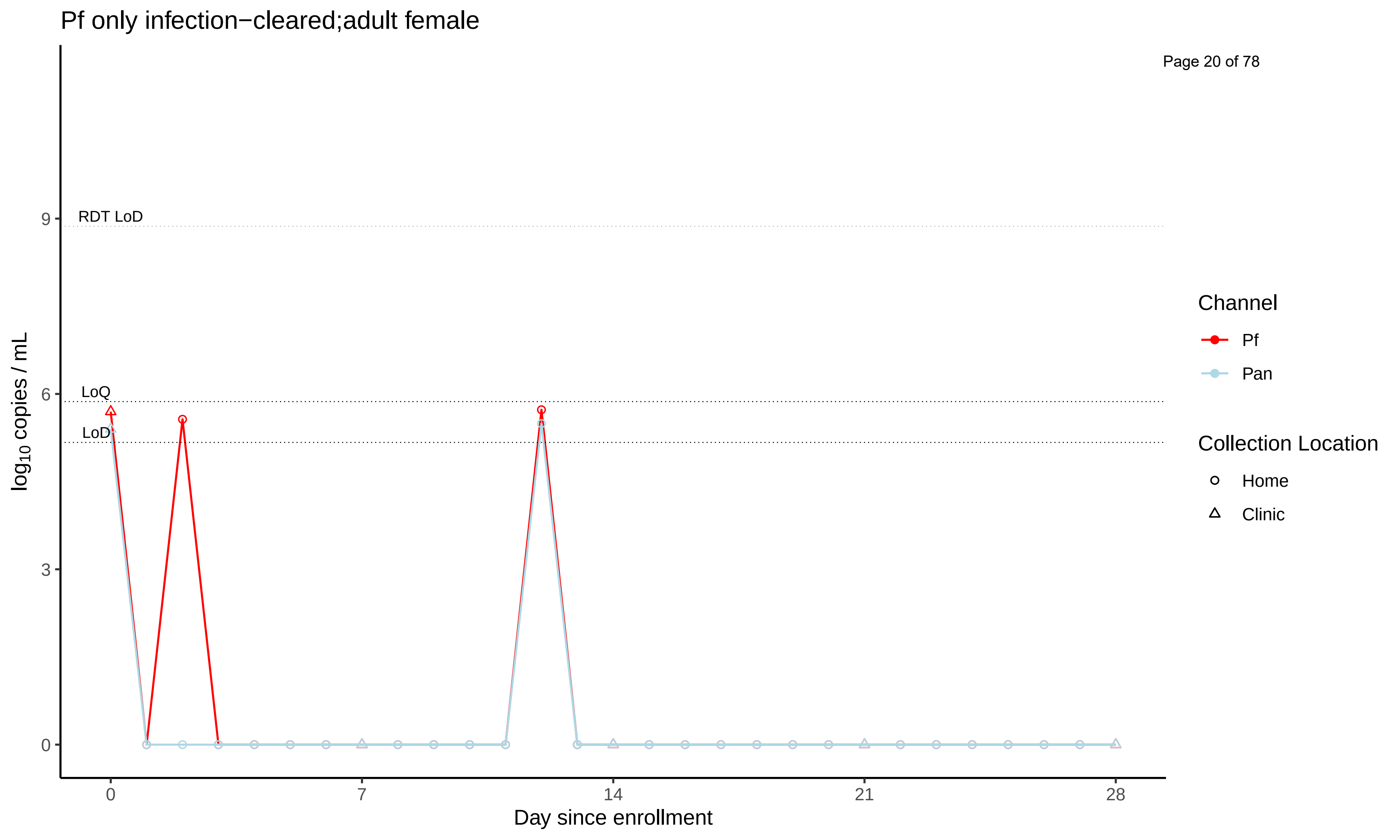
Pf only infection−cleared;adult female
RDT LoD
LoQ
LoD
9
6
3
0
0
7
14
21
28
$\log_{10}$ copies / mL
Day since enrollment
Channel
Pf
Pan
Collection Location
Home
Clinic

Pf only infection−cleared;adult male

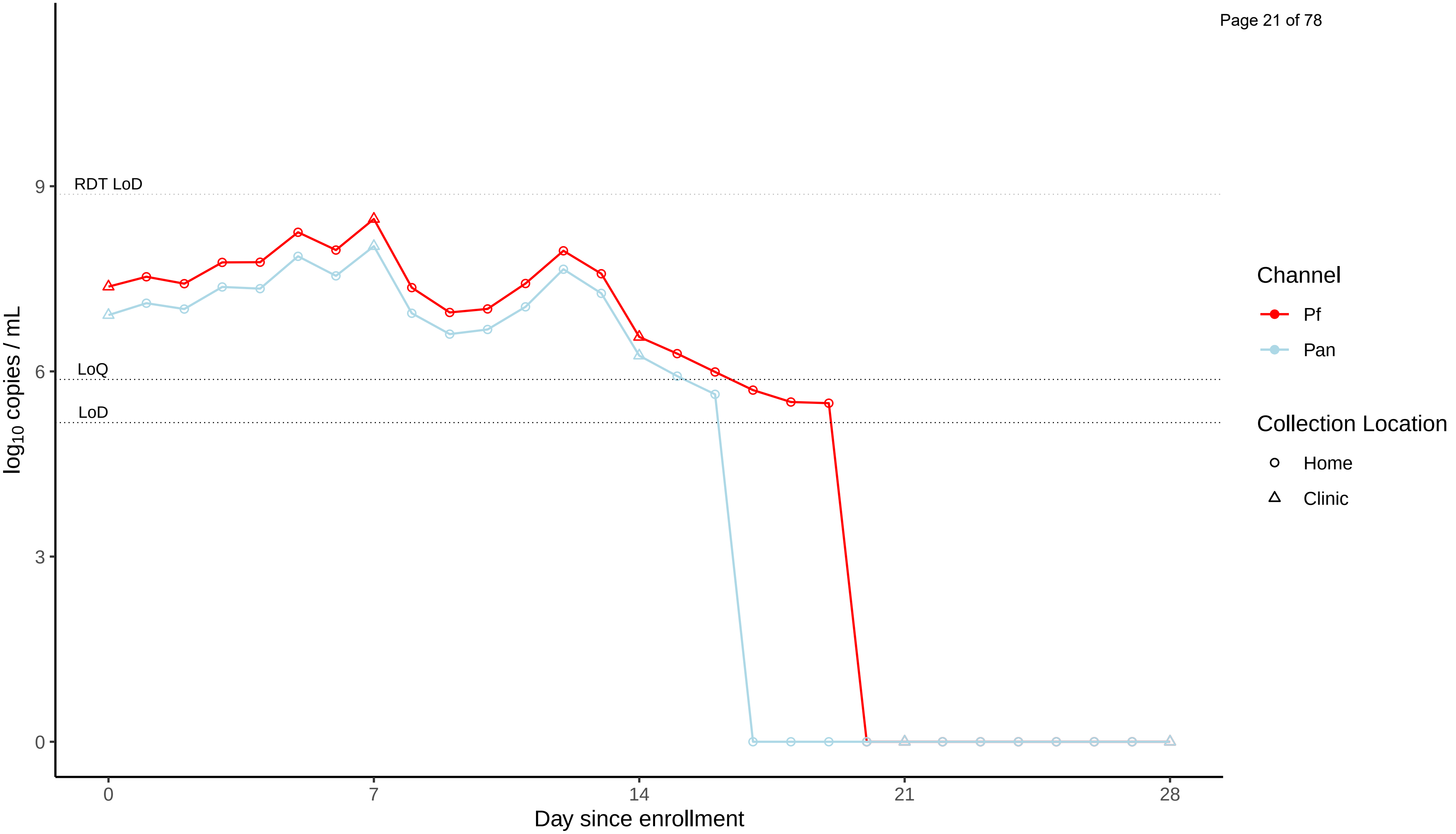

Pf only infection−cleared;male child

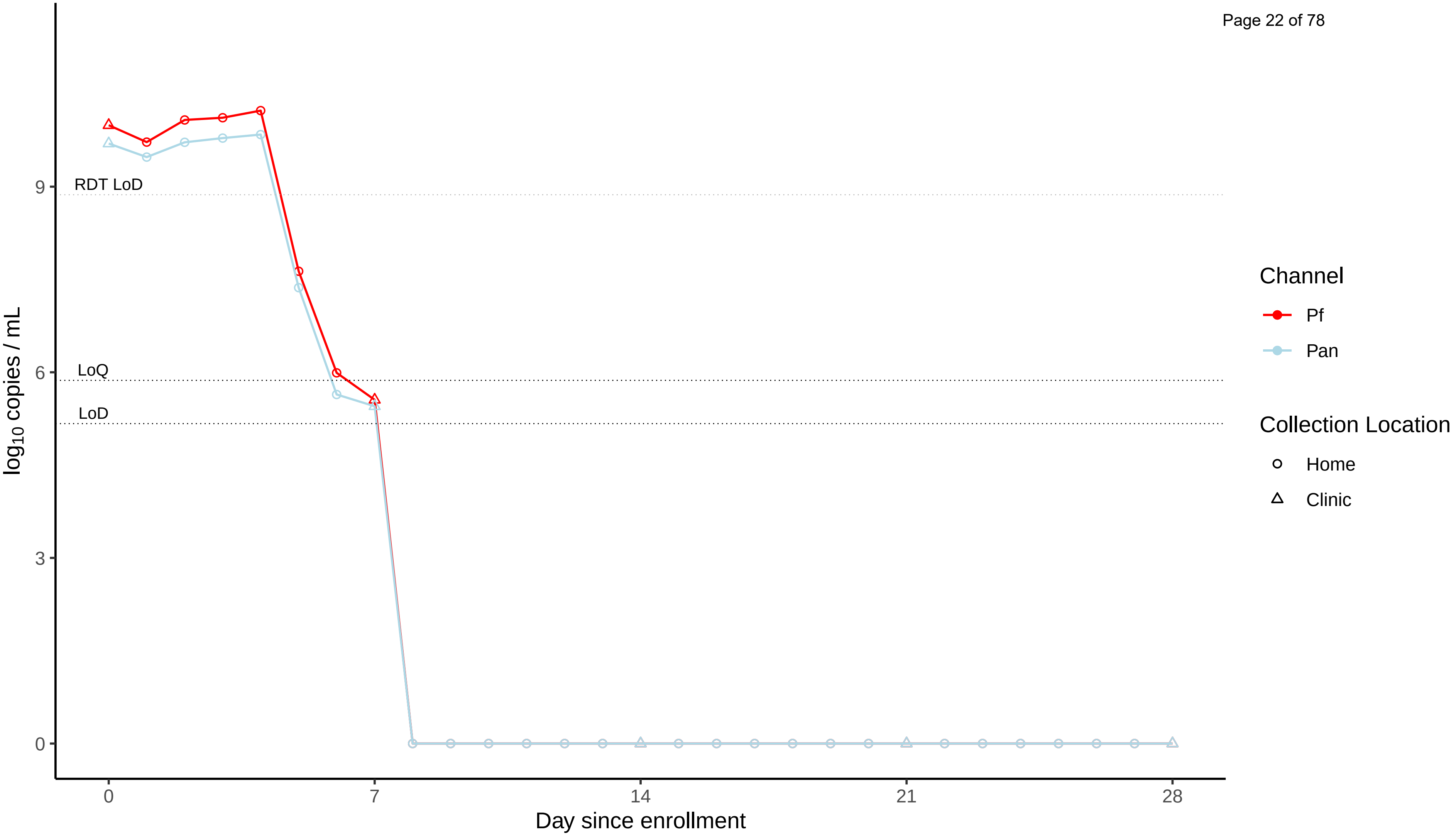

Pf only infection−cleared + newly acquired;adult female

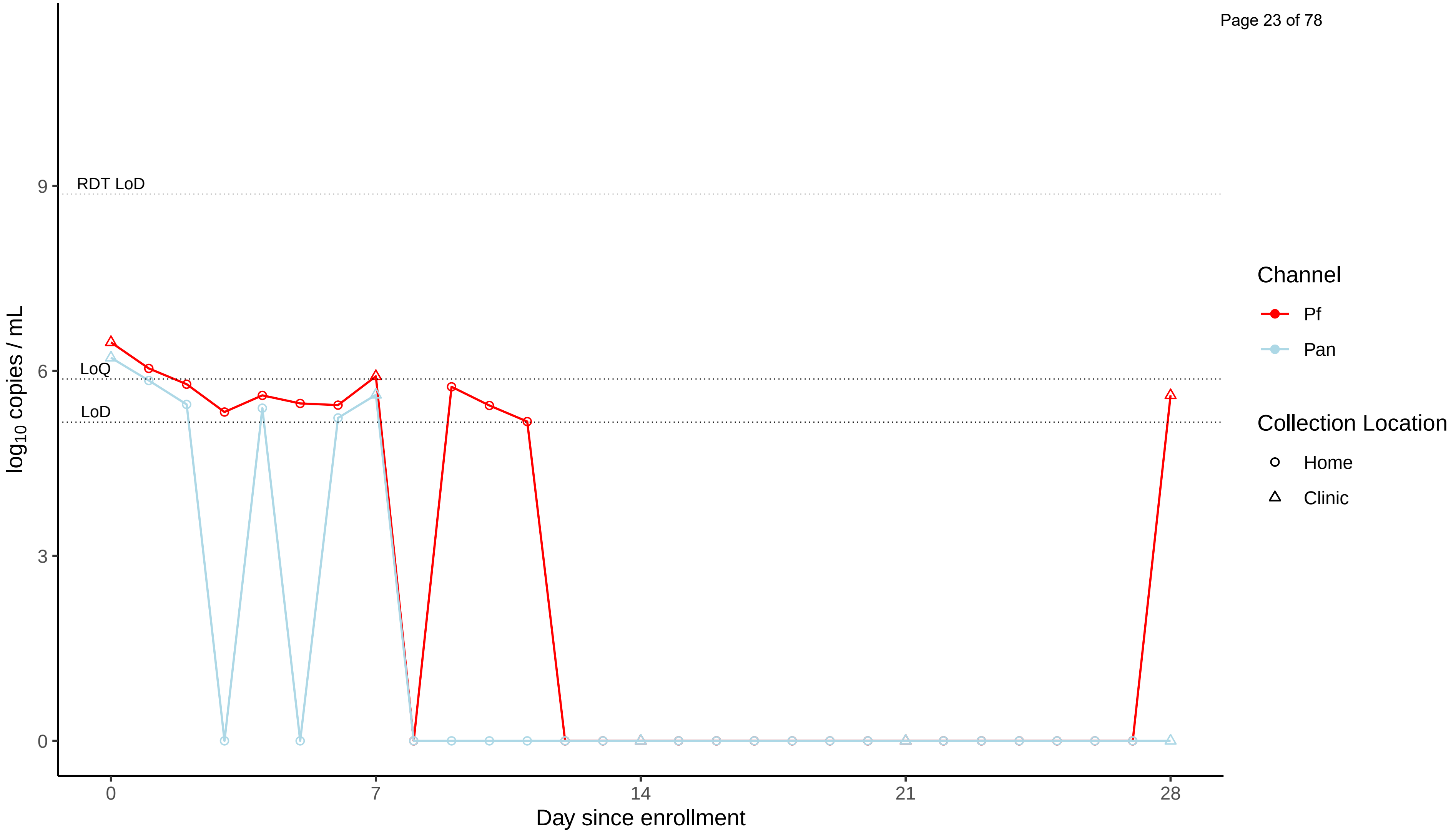

Pf only infection−indeterminate;adult female

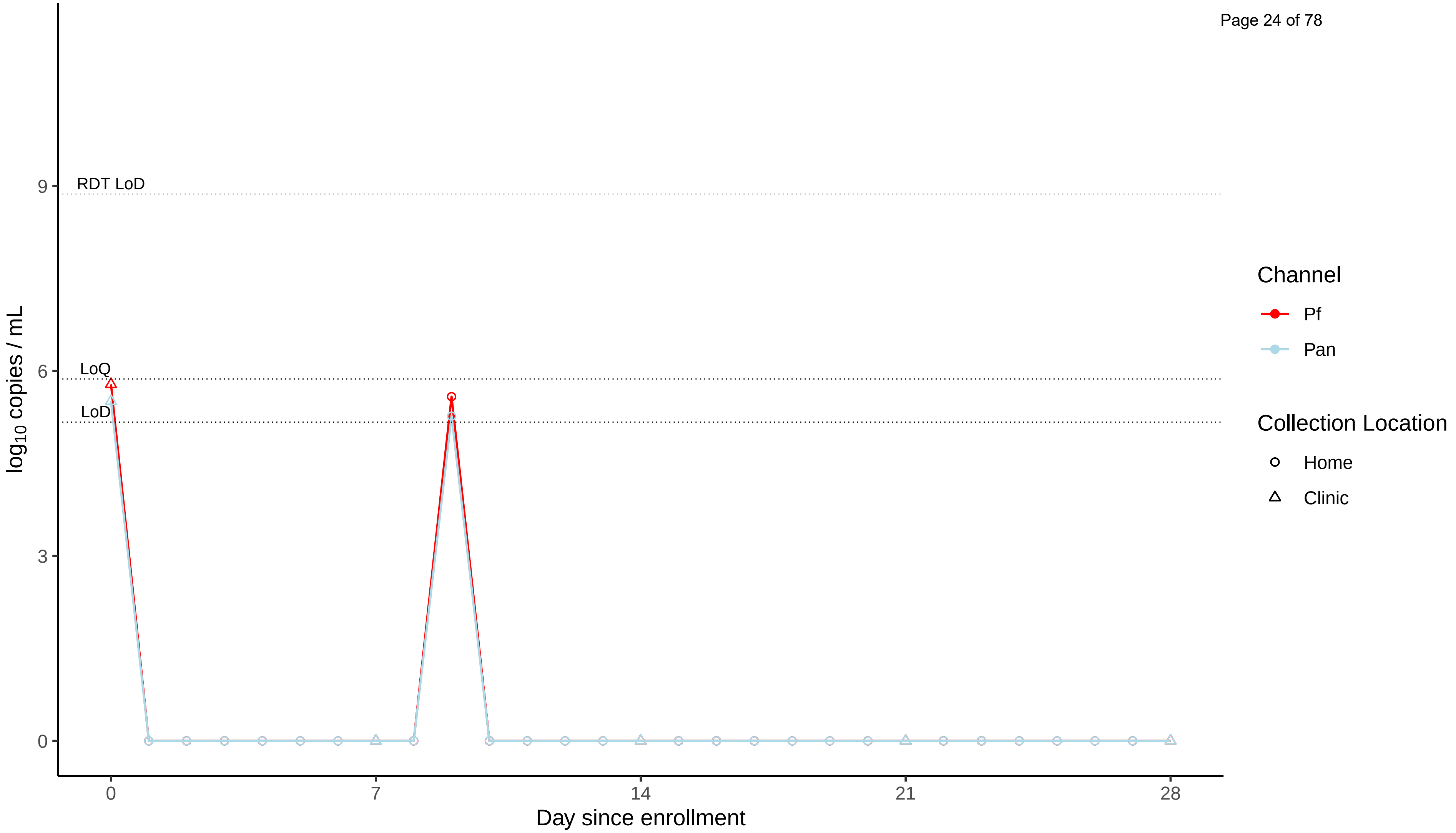

Pf only infection−indeterminate;adult male

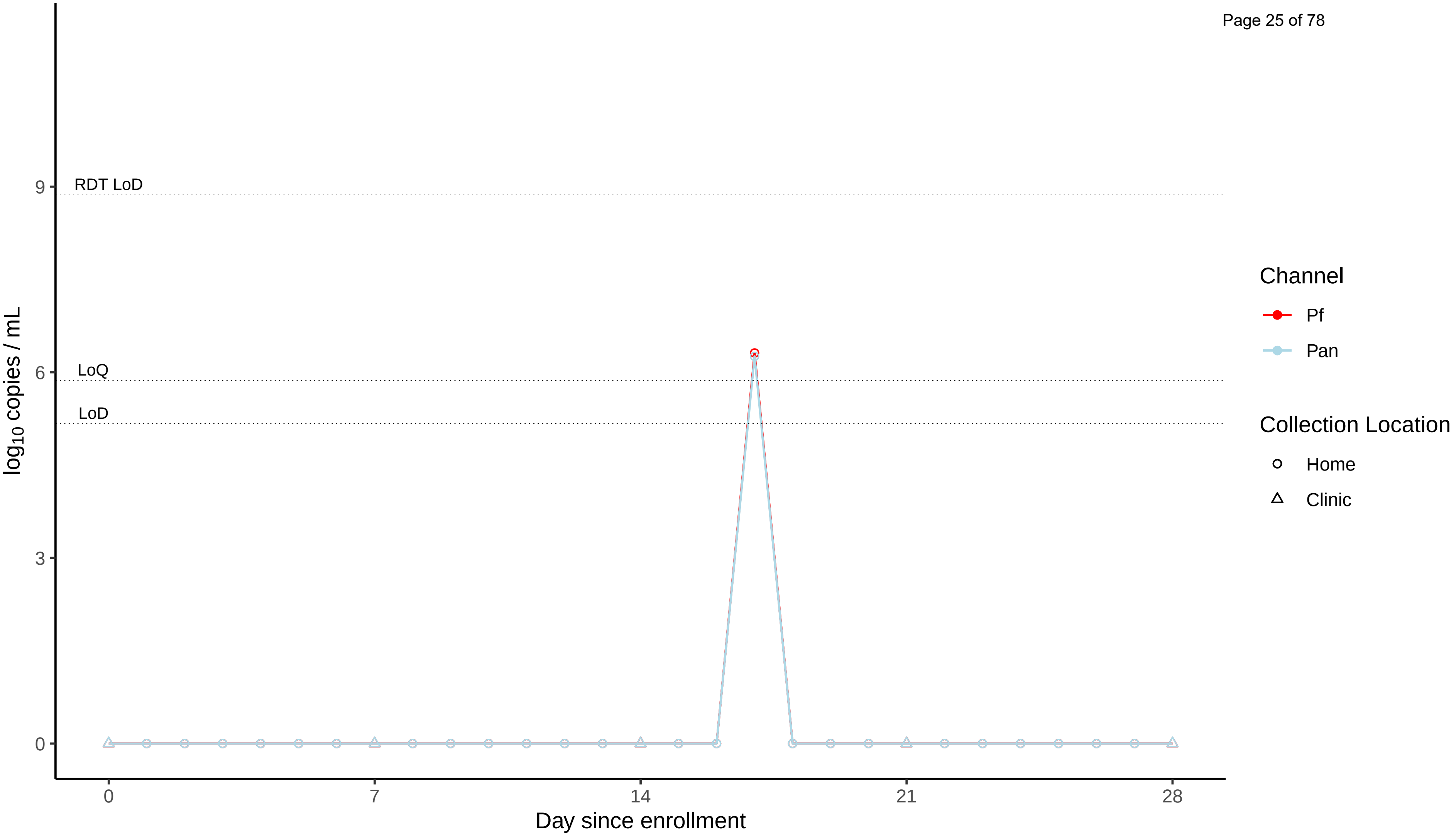

Pf only infection−newly acquired;female child

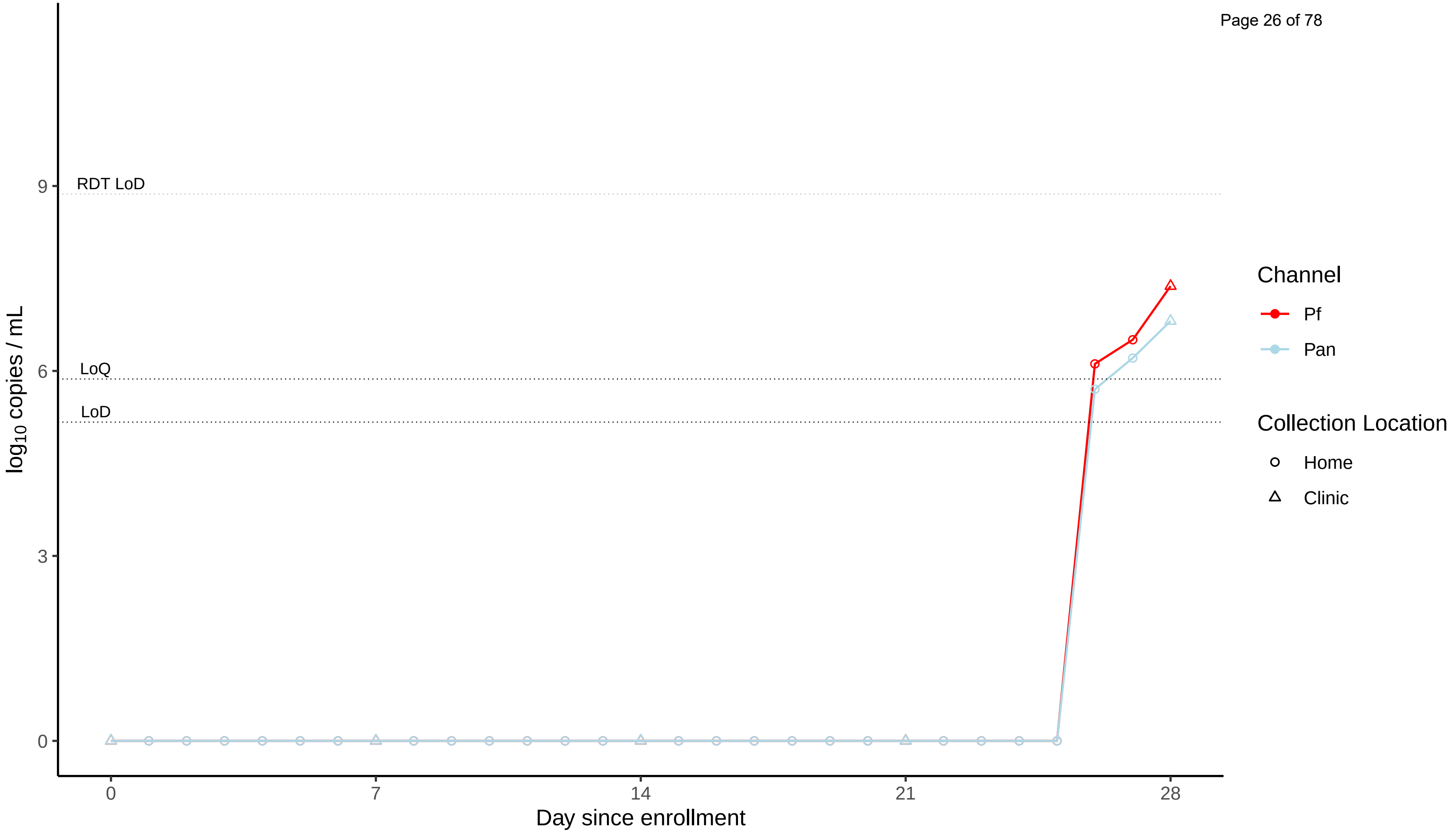

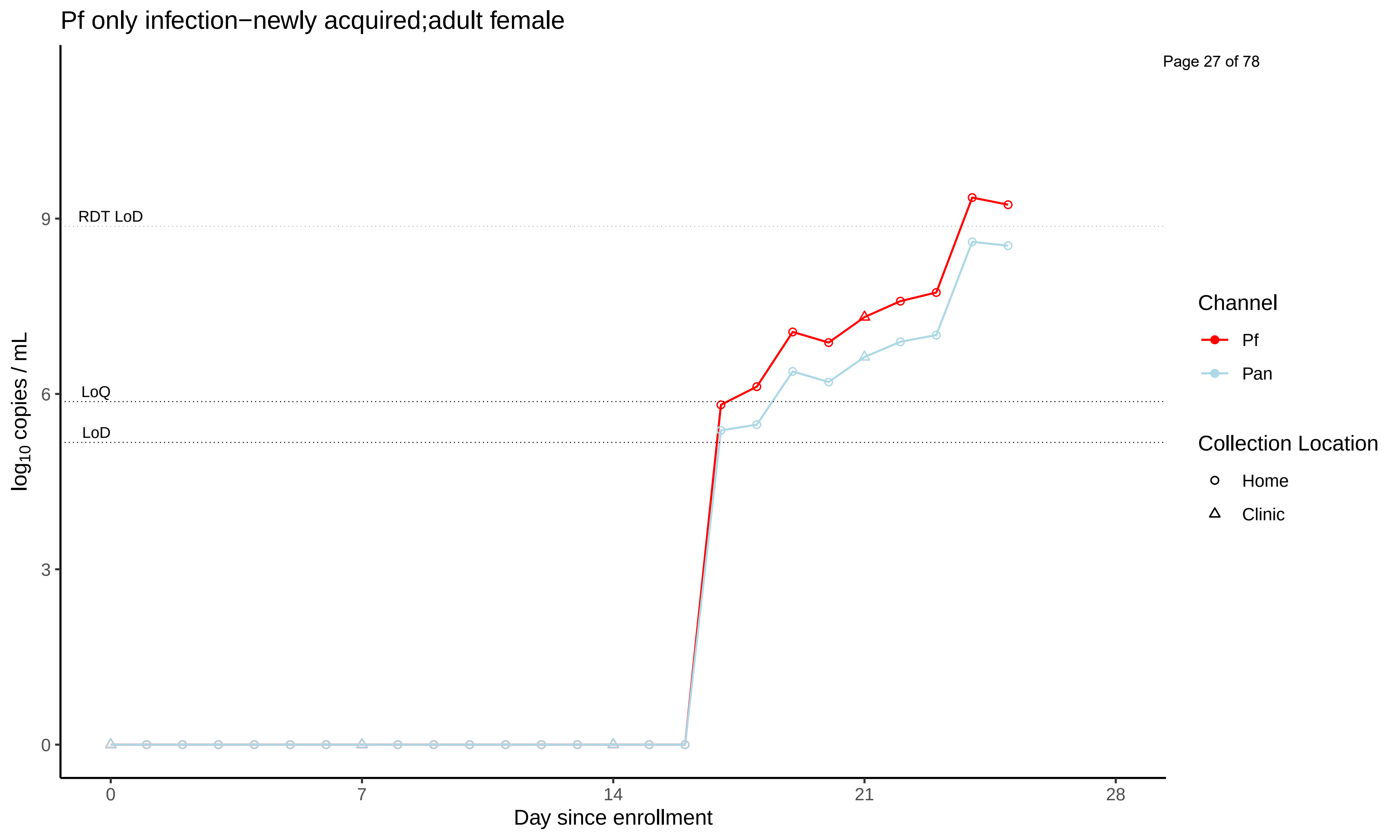
Pf only infection−newly acquired;adult female
Page 27 of 78
RDT LoD
LoQ
LoD
log10 copies / mL
0
3
6
9
0
7
14
21
28
Day since enrollment
Channel
Pf
Pan
Collection Location
Home
Clinic

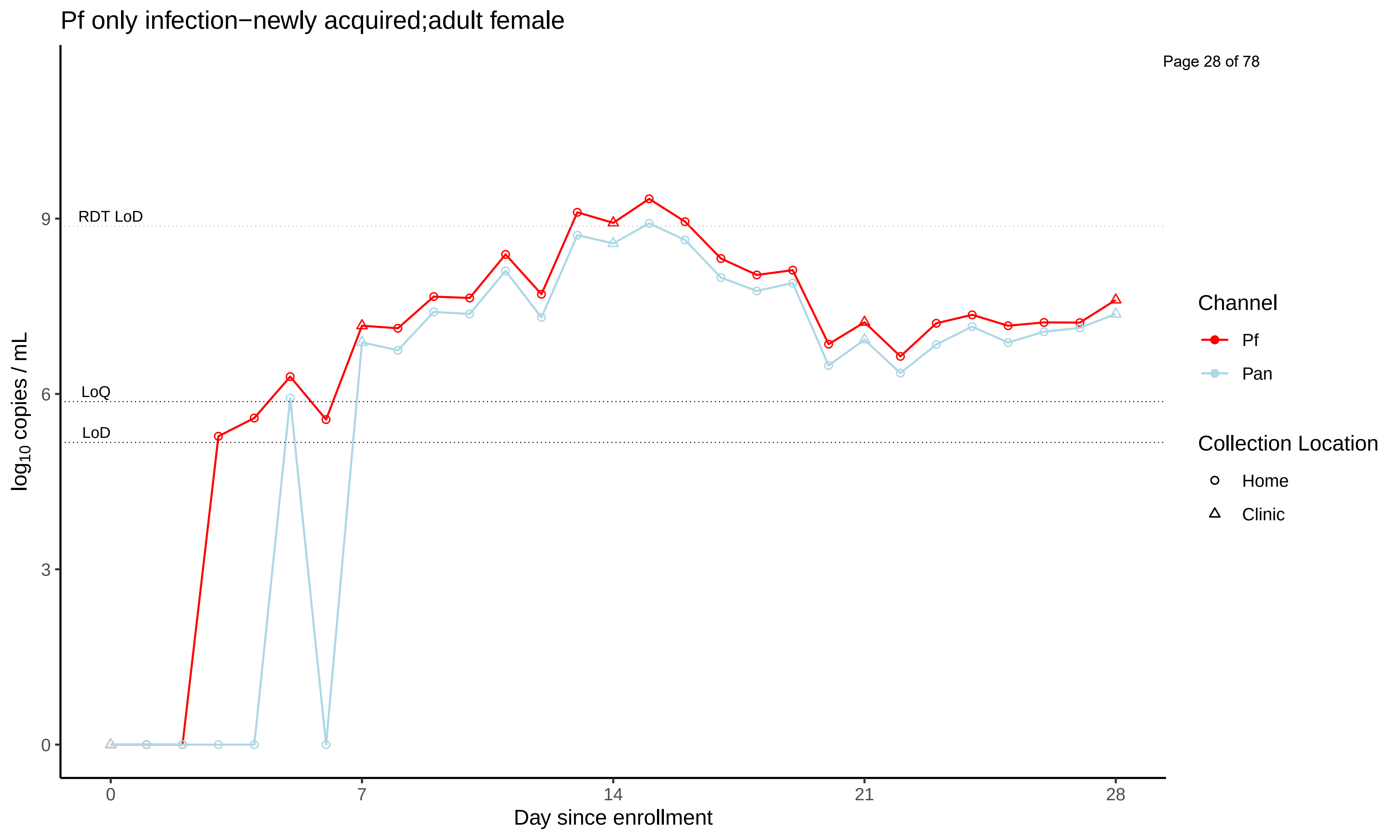
Pf only infection−newly acquired;adult female
RDT LoD
LoQ
LoD
9
6
3
0
$\log_{10}$ copies / mL
0
7
14
21
28
Day since enrollment
Channel
Pf
Pan
Collection Location
Home
Clinic

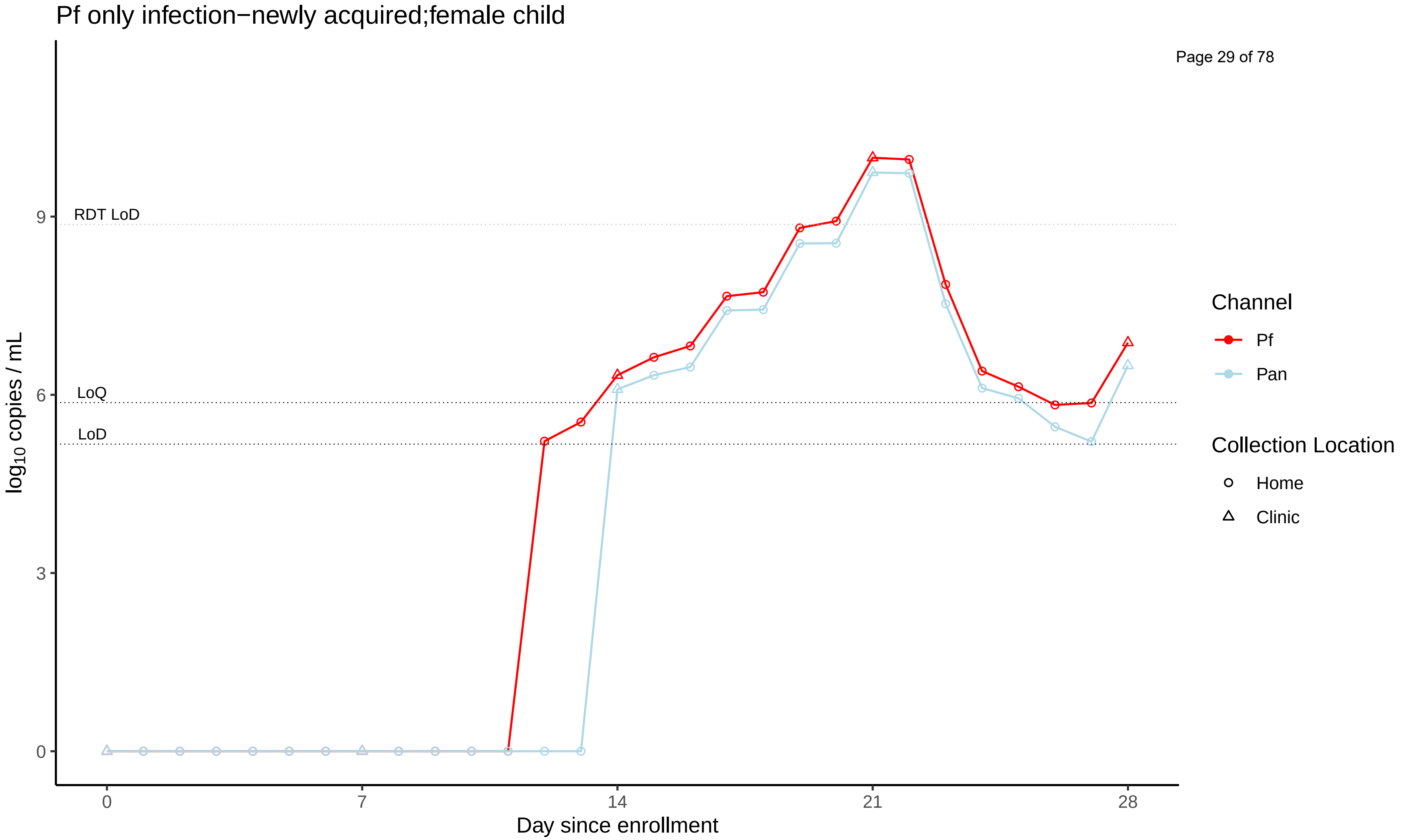
Pf only infection−newly acquired;female child
RDT LoD
LoQ
LoD
9
6
3
0
$\log_{10}$ copies / mL
0
7
14
21
28
Day since enrollment
Channel
Pf
Pan
Collection Location
Home
Clinic

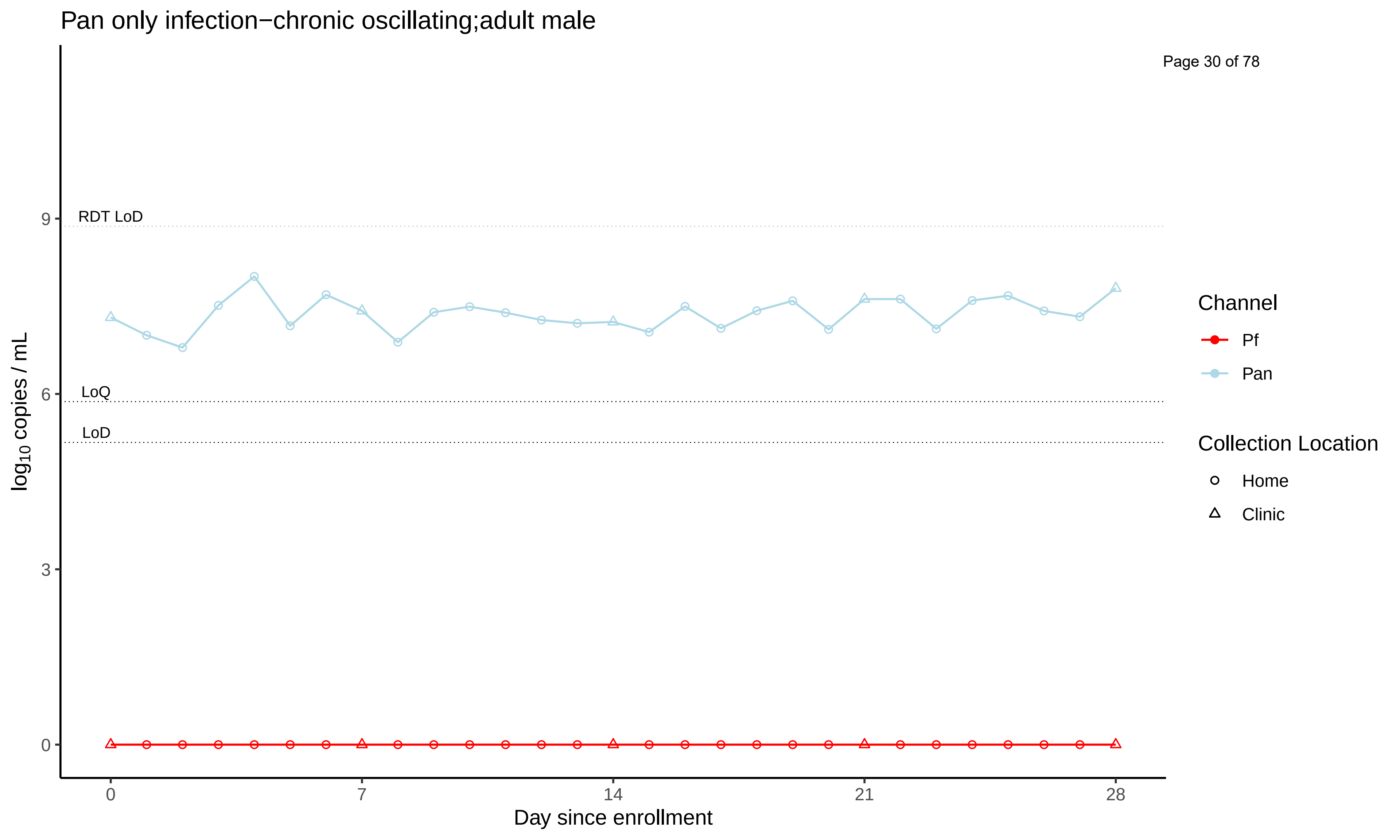

Pan only infection−chronic oscillating;adult male
Page 30 of 78
RDT LoD
LoQ
LoD
9
6
3
0
log10 copies / mL
0
7
14
21
28
Day since enrollment
Channel
Pf
Pan
Collection Location
Home
Clinic

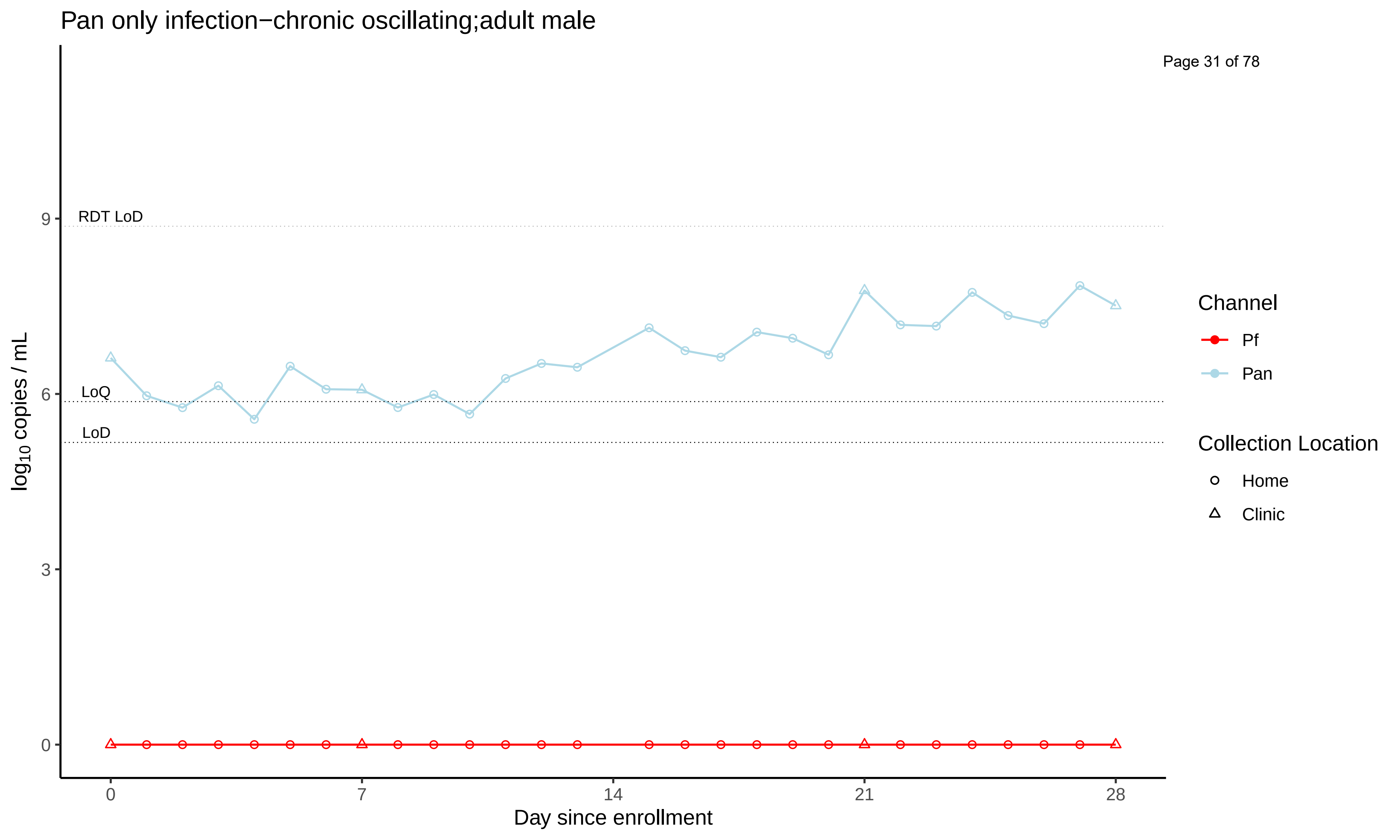
Pan only infection−chronic oscillating;adult male
RDT LoD
LoQ
LoD
9
6
3
0
0
7
14
21
28
Day since enrollment
log10 copies / mL
Channel
Pf
Pan
Collection Location
Home
Clinic

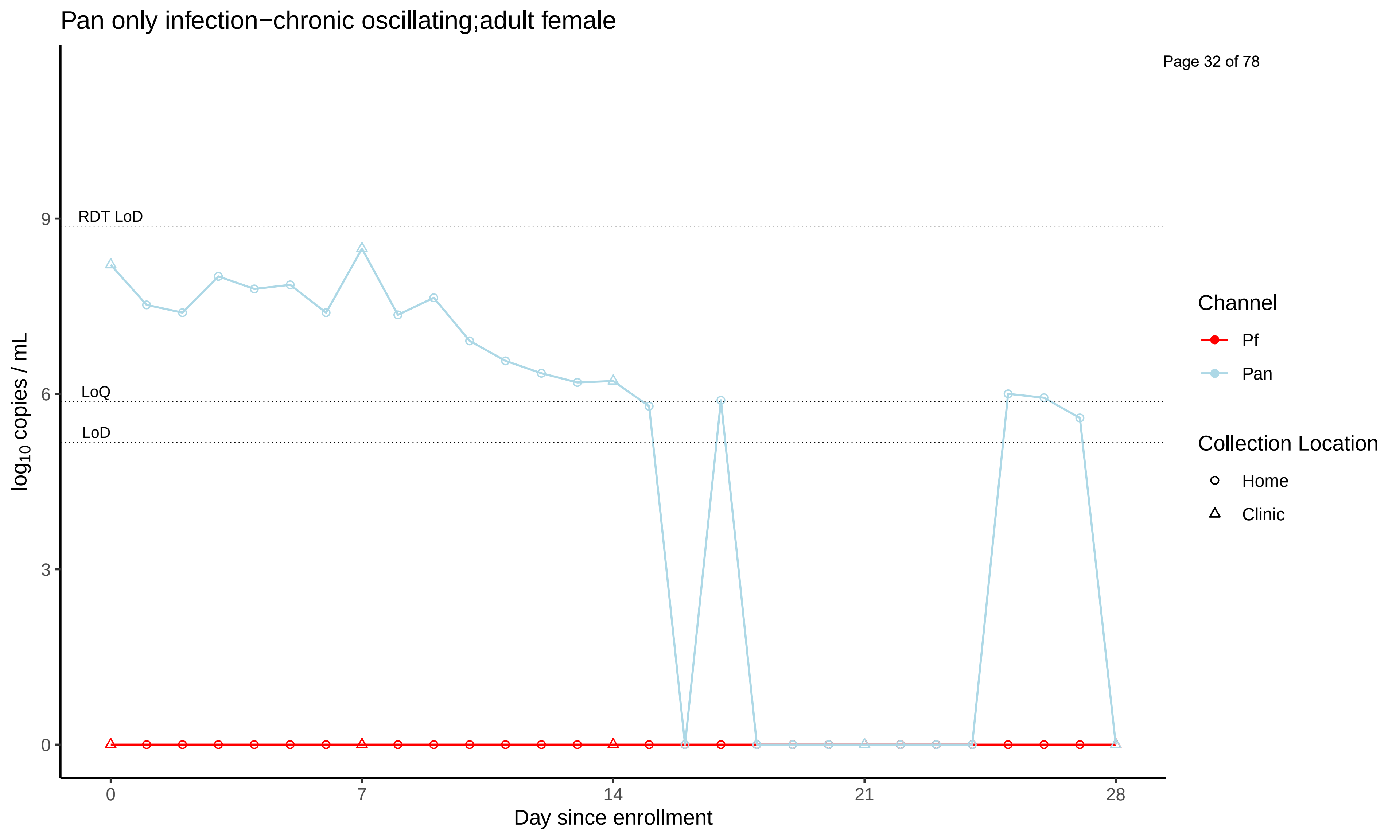
Pan only infection−chronic oscillating;adult female
Page 32 of 78
RDT LoD
LoQ
LoD
log10 copies / mL
9
6
3
0
0
7
14
21
28
Day since enrollment
Channel
Pf
Pan
Collection Location
Home
Clinic

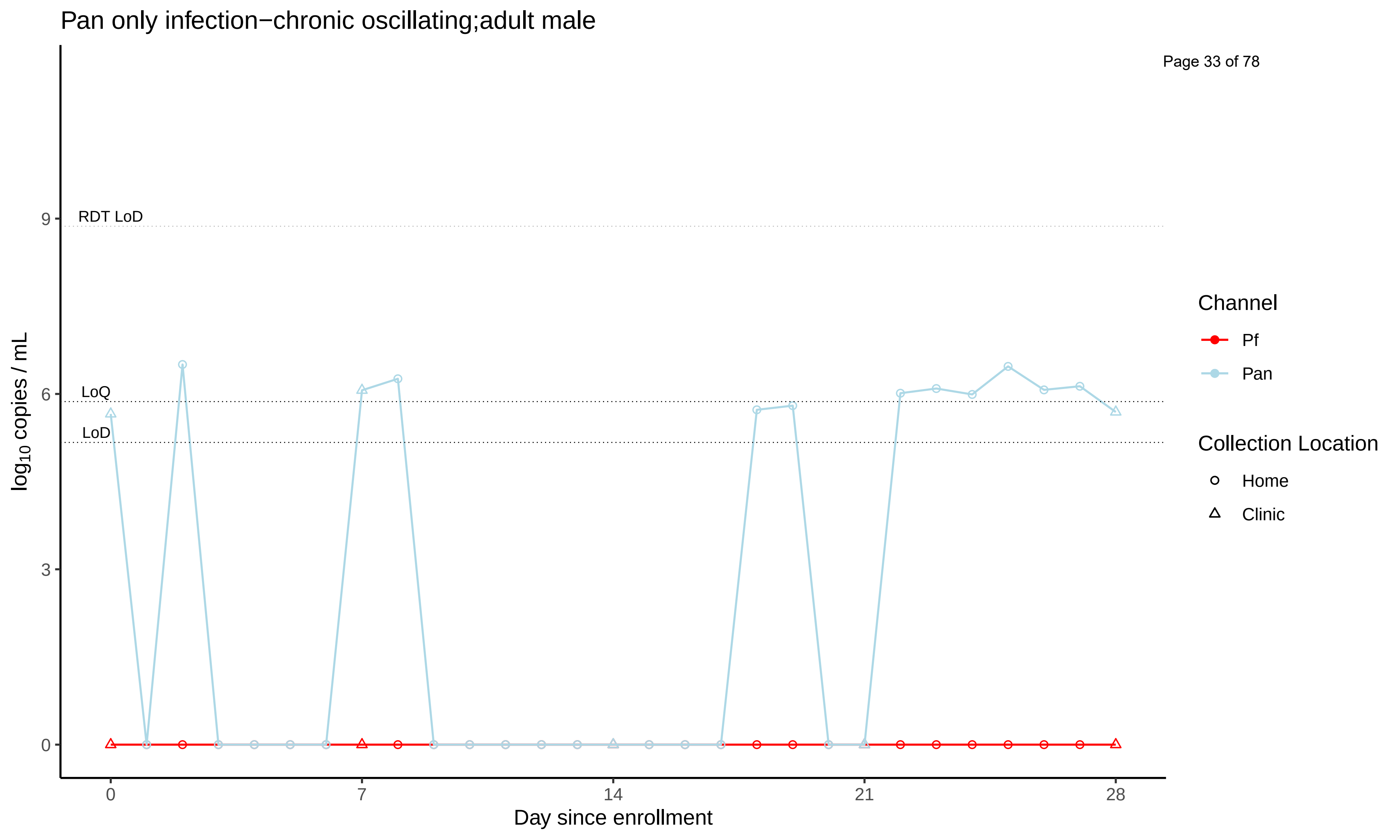
Pan only infection−chronic oscillating;adult male
RDT LoD
LoQ
LoD
9
6
3
0
log_{10} copies / mL
0
7
14
21
28
Day since enrollment
Channel
Pf
Pan
Collection Location
Home
Clinic

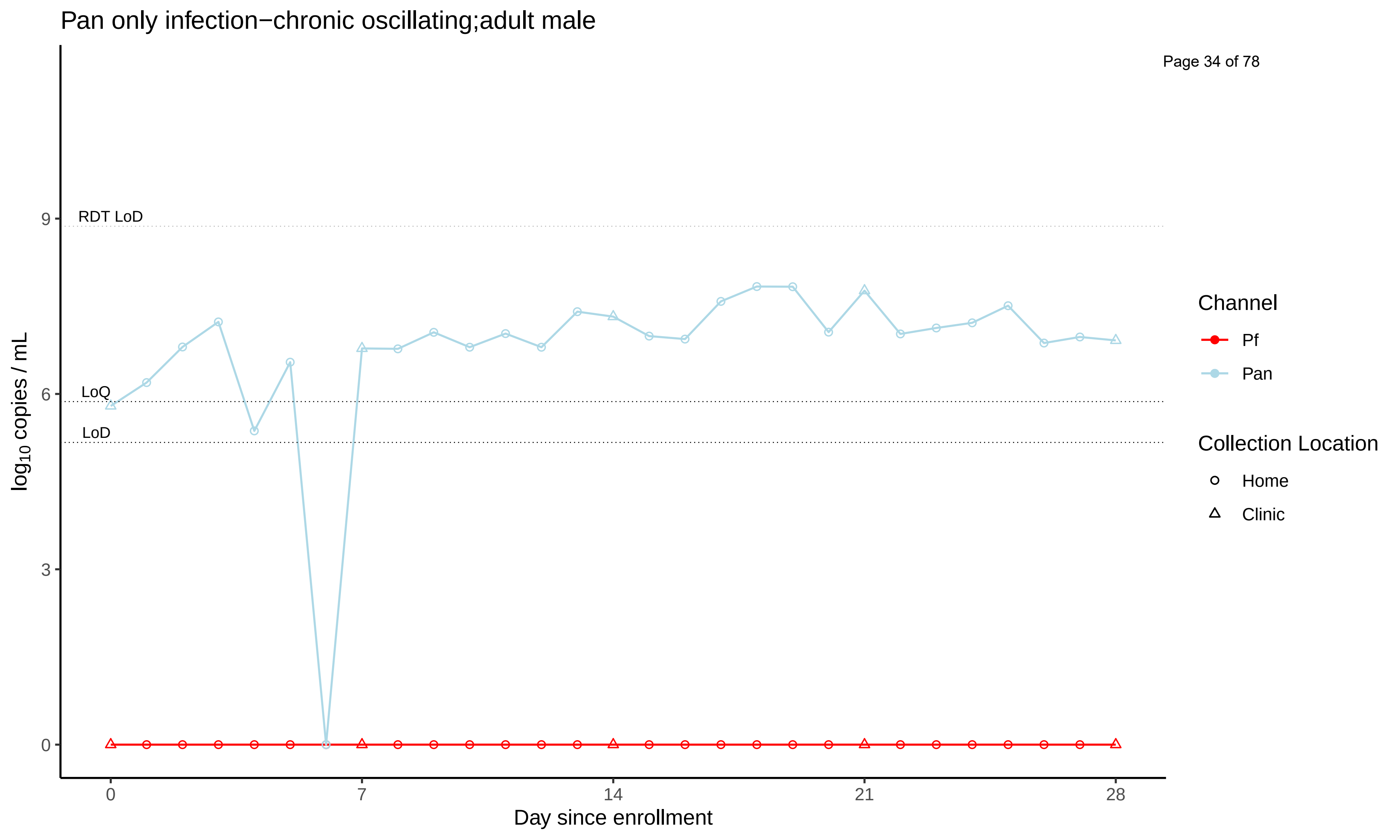
Pan only infection−chronic oscillating;adult male
RDT LoD
LoQ
LoD
9
6
3
0
$\log_{10}$ copies / mL
0
7
14
21
28
Day since enrollment
Channel
Pf
Pan
Collection Location
Home
Clinic

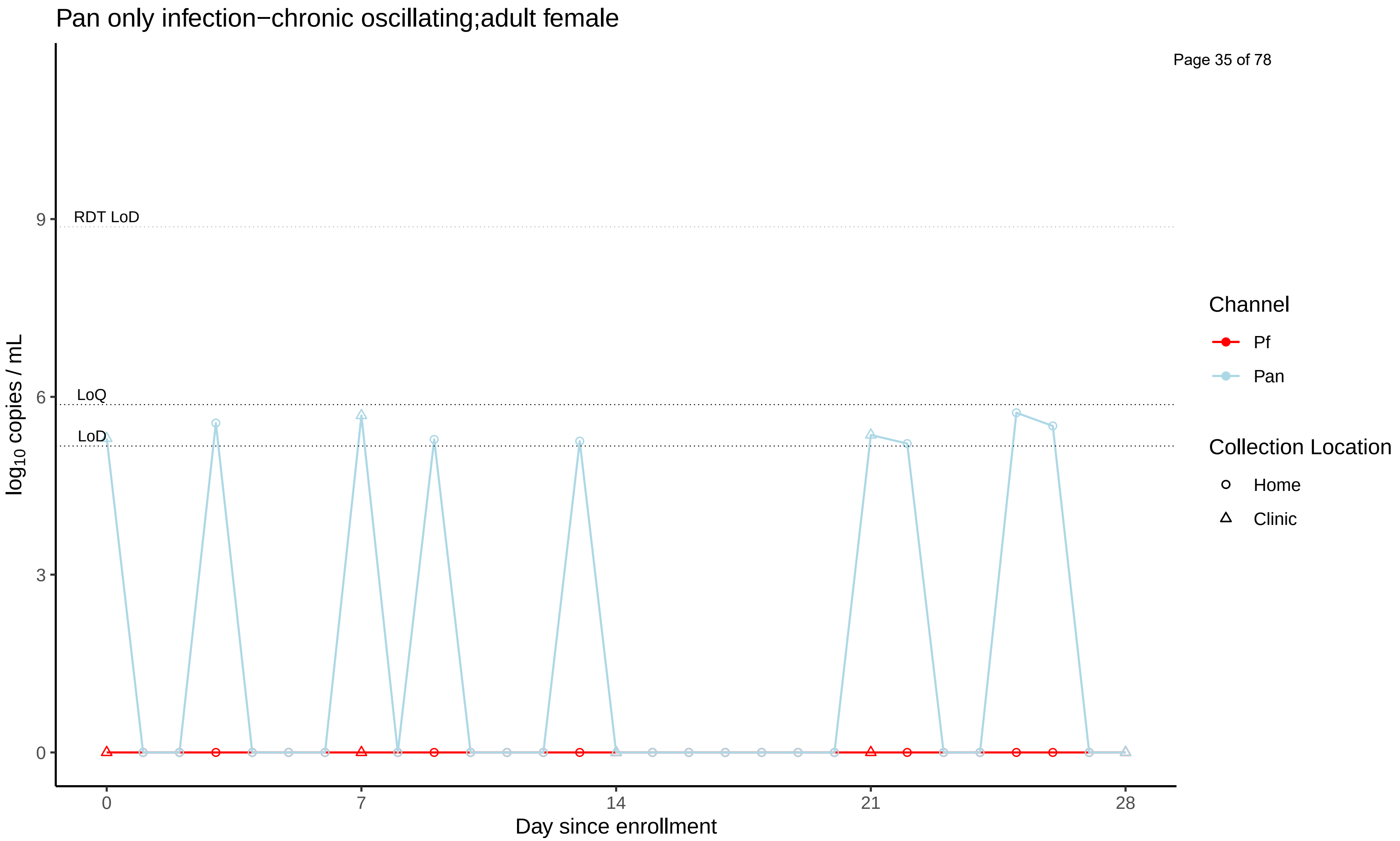
Pan only infection−chronic oscillating;adult female
RDT LoD
LoQ
LoD
9
6
3
0
log10 copies / mL
0
7
14
21
28
Day since enrollment
Channel
Pf
Pan
Collection Location
Home
Clinic

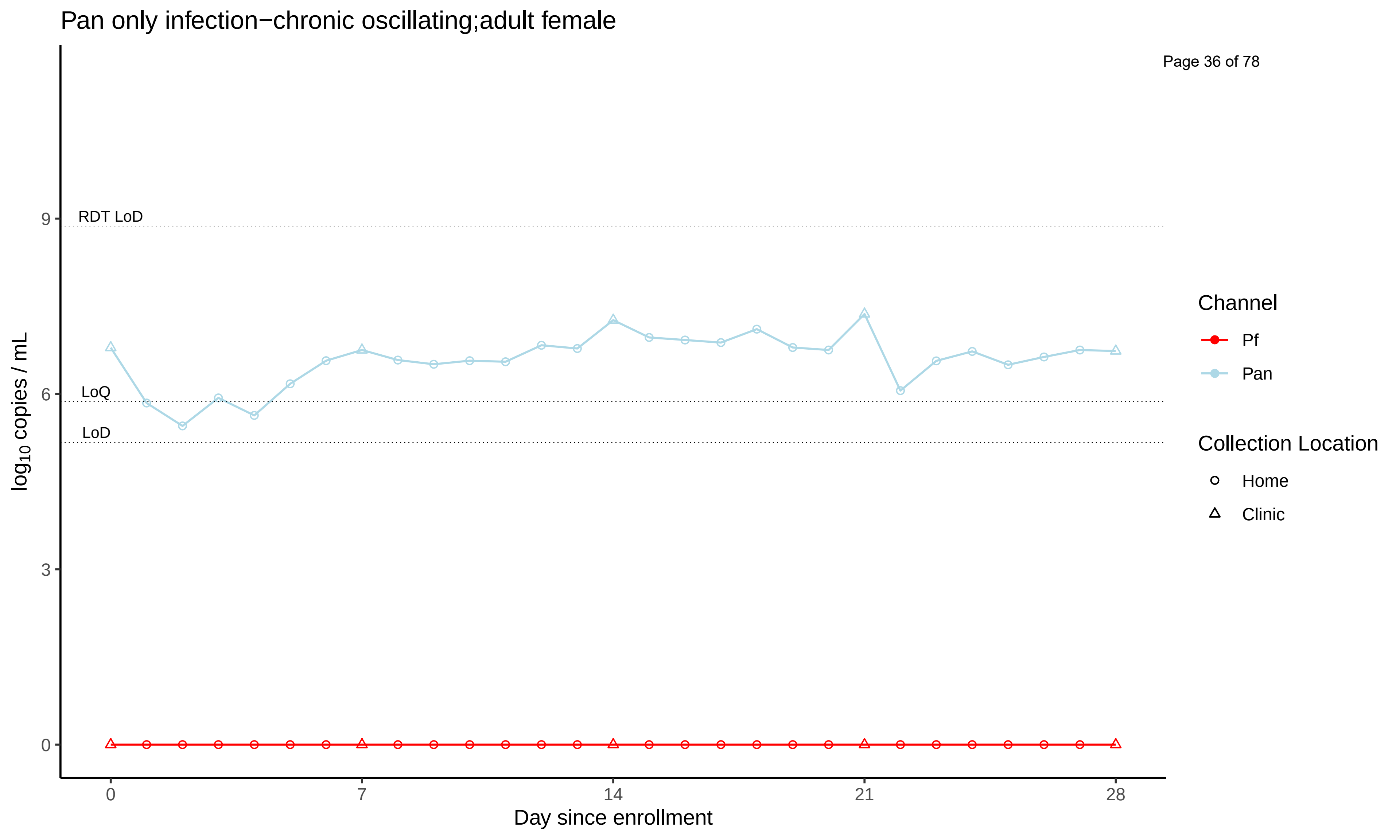
Pan only infection−chronic oscillating;adult female
RDT LoD
LoQ
LoD
9
6
3
0
$\log_{10}$ copies / mL
0
7
14
21
28
Day since enrollment
Channel
Pf
Pan
Collection Location
Home
Clinic

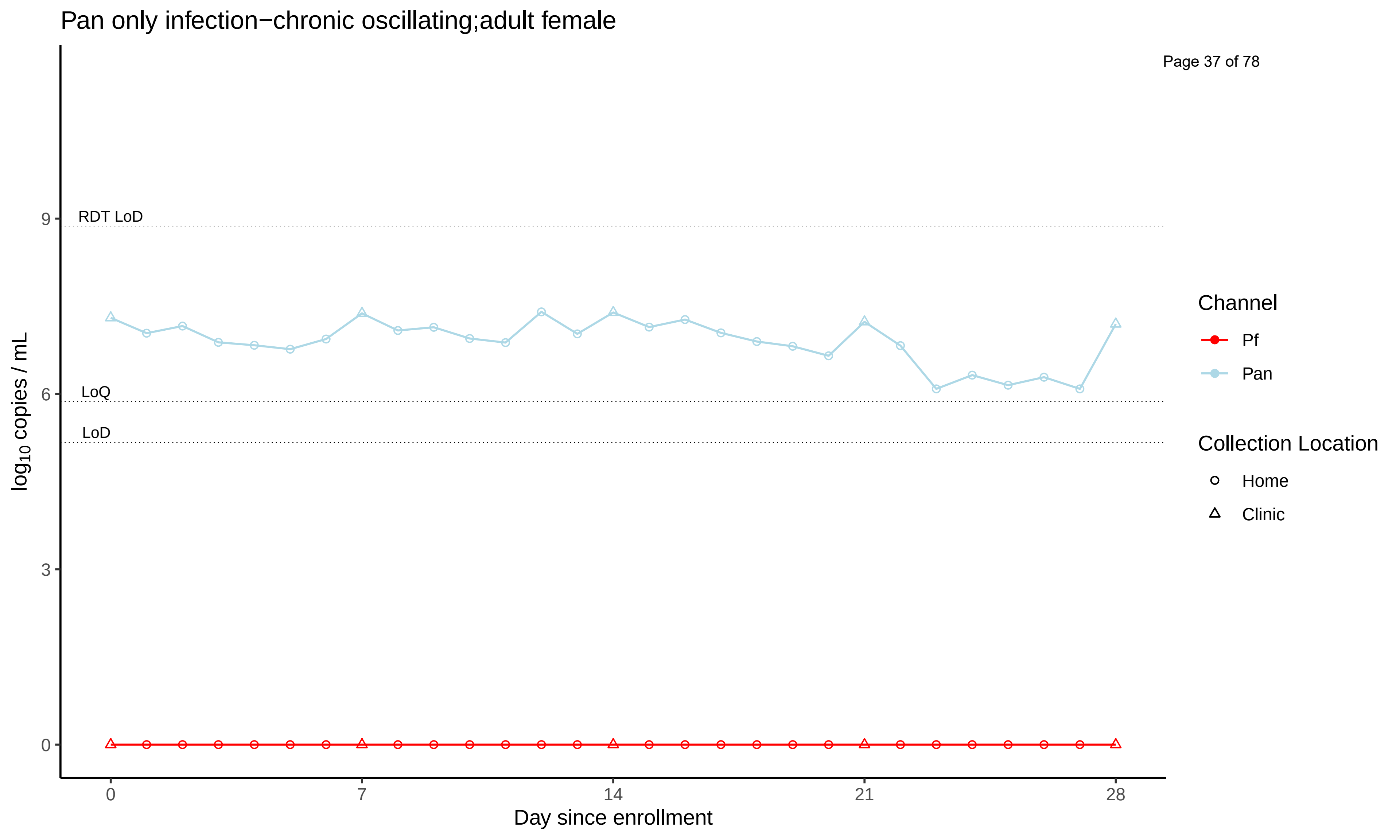
Pan only infection−chronic oscillating;adult female
RDT LoD
LoQ
LoD
9
6
3
0
log10 copies / mL
0
7
14
21
28
Day since enrollment
Channel
Pf
Pan
Collection Location
Home
Clinic

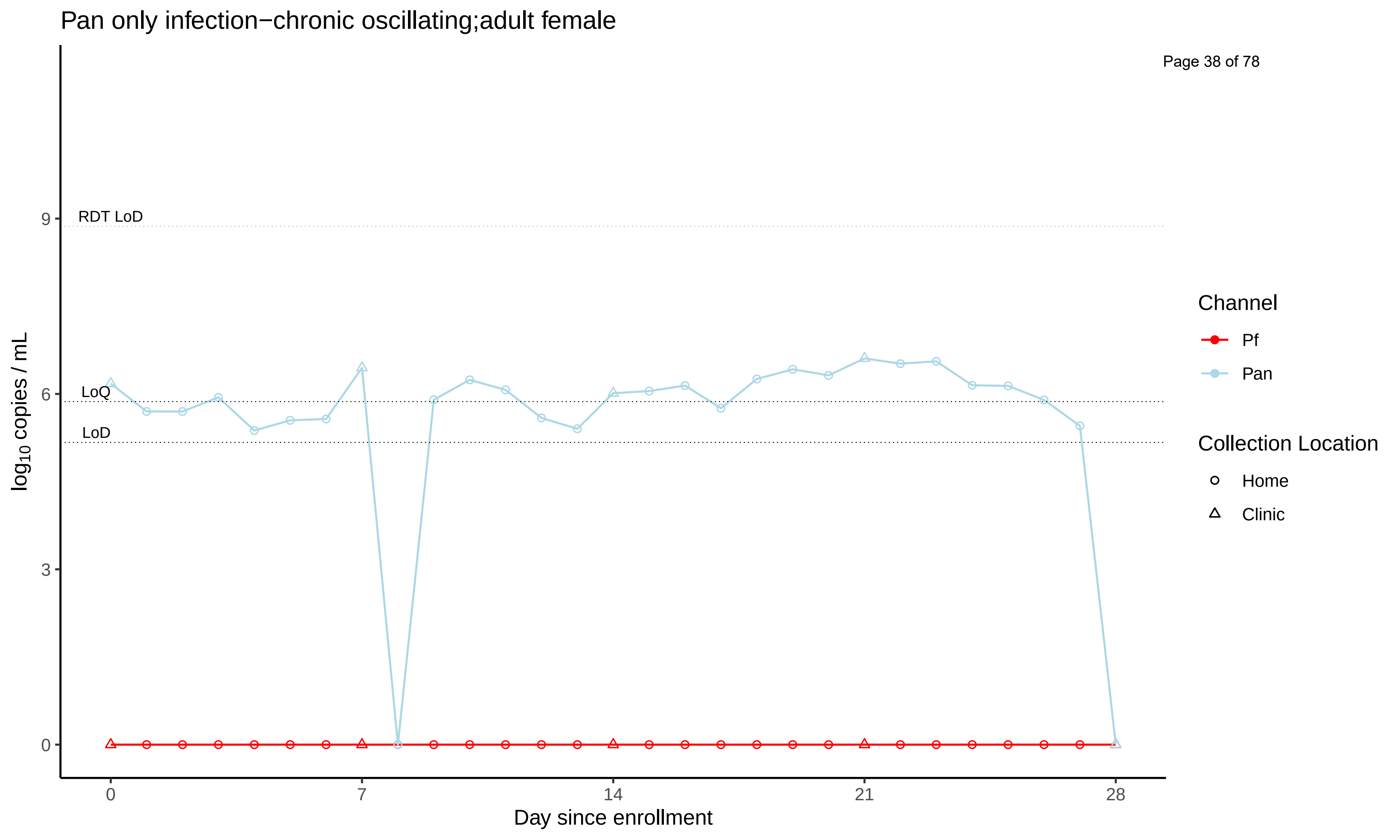
Pan only infection−chronic oscillating;adult female
RDT LoD
LoQ
LoD
9
6
3
0
0
7
14
21
28
Day since enrollment
log10 copies / mL
Channel
Pf
Pan
Collection Location
Home
Clinic

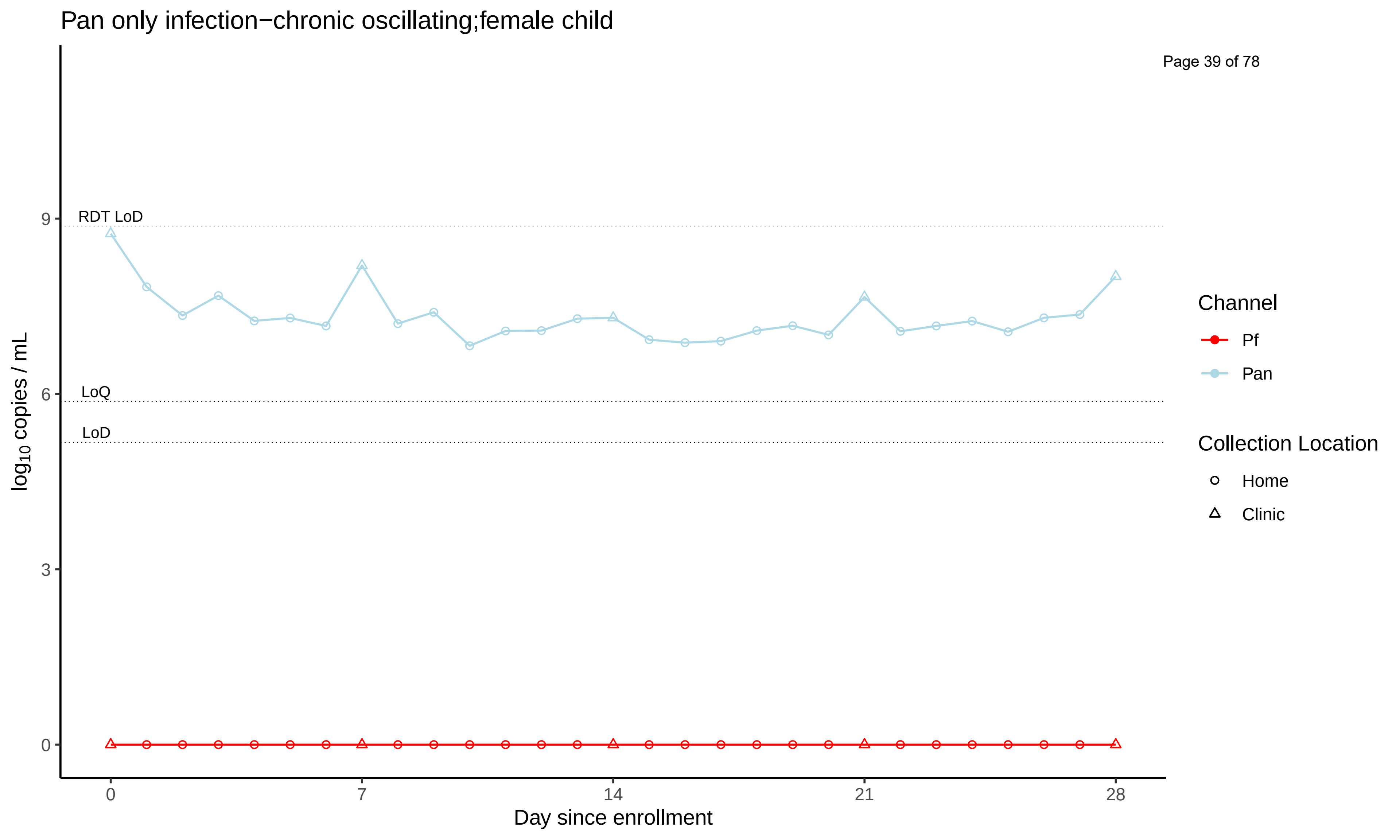
Pan only infection−chronic oscillating;female child
RDT LoD
LoQ
LoD
log10 copies / mL
0
3
6
9
0
7
14
21
28
Day since enrollment
Channel
Pf
Pan
Collection Location
Home
Clinic

Pan only infection−cleared;male child

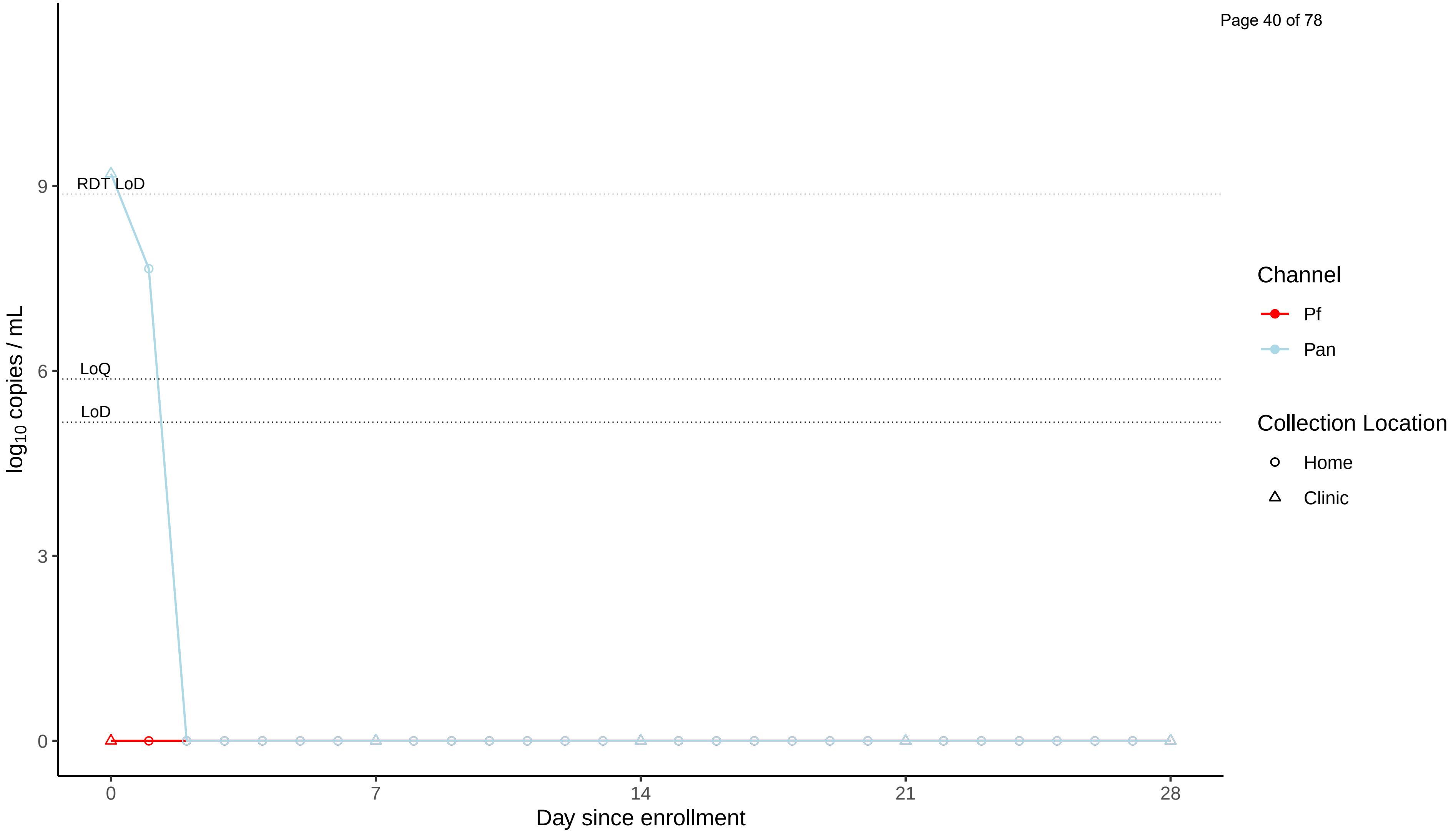

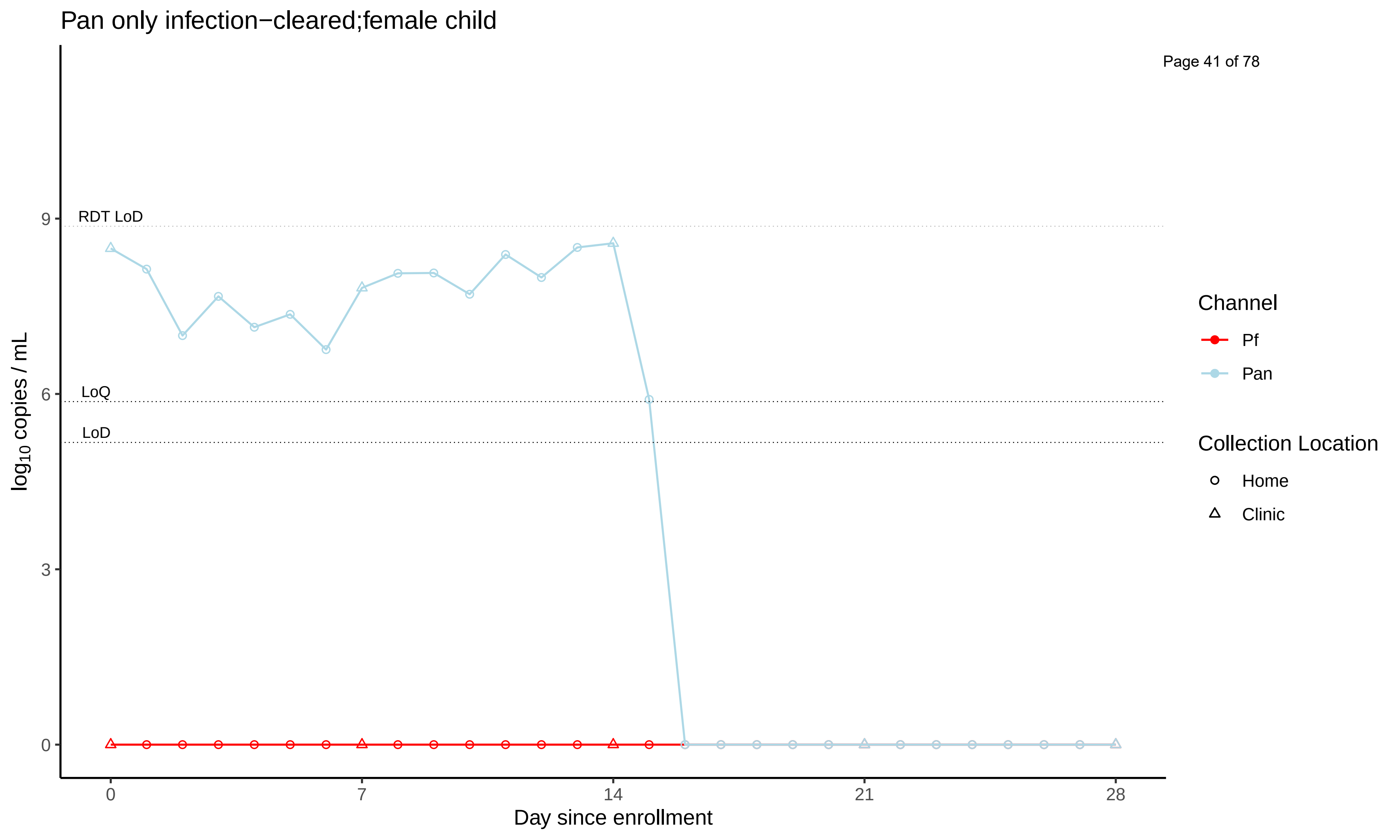
Pan only infection−cleared;female child
RDT LoD
LoQ
LoD
$\log_{10}$ copies / mL
0
3
6
9
0
7
14
21
28
Day since enrollment
Channel
Pf
Pan
Collection Location
Home
Clinic

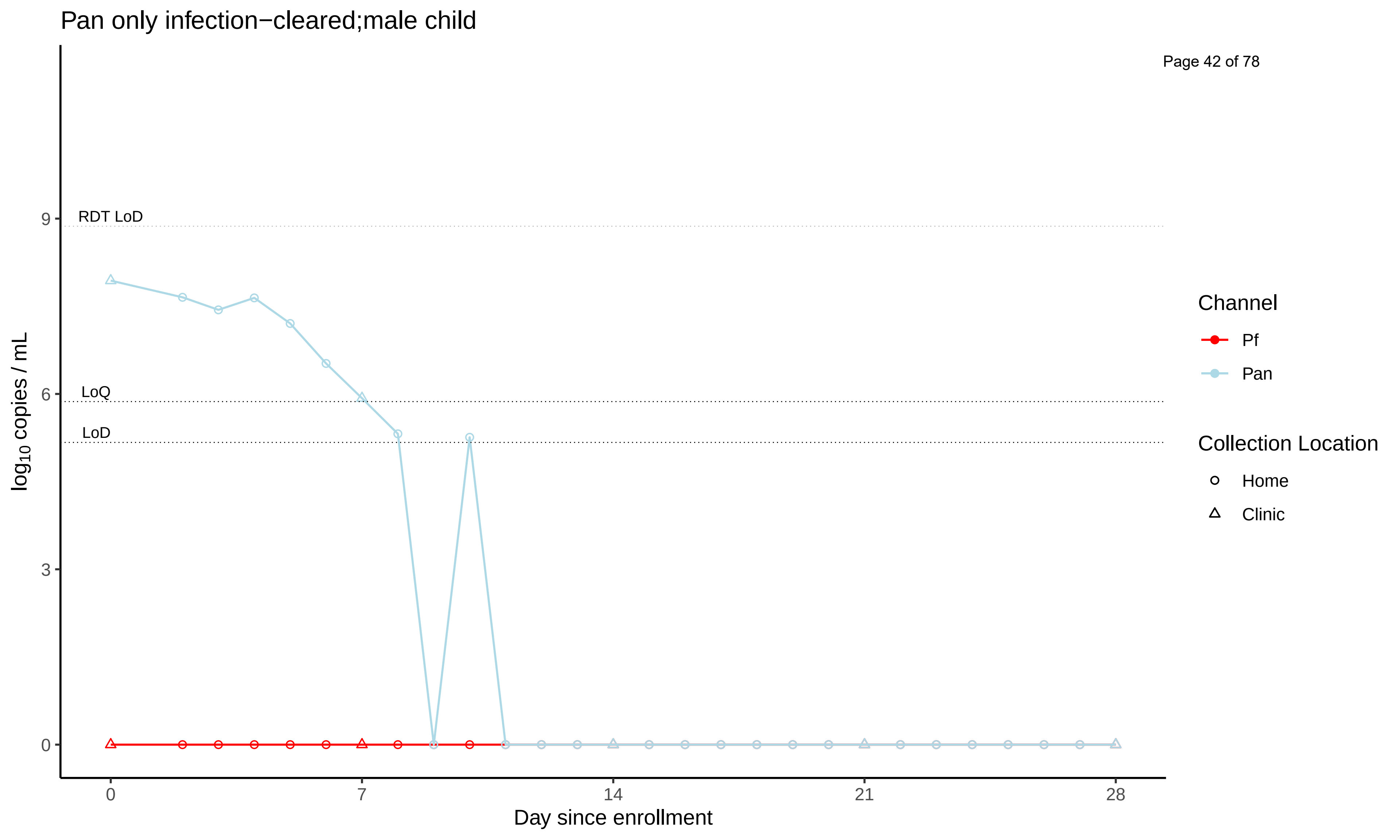

Pan only infection−cleared;male child
RDT LoD
LoQ
LoD
9
6
3
0
log10 copies / mL
0
7
14
21
28
Day since enrollment
Channel
Pf
Pan
Collection Location
Home
Clinic

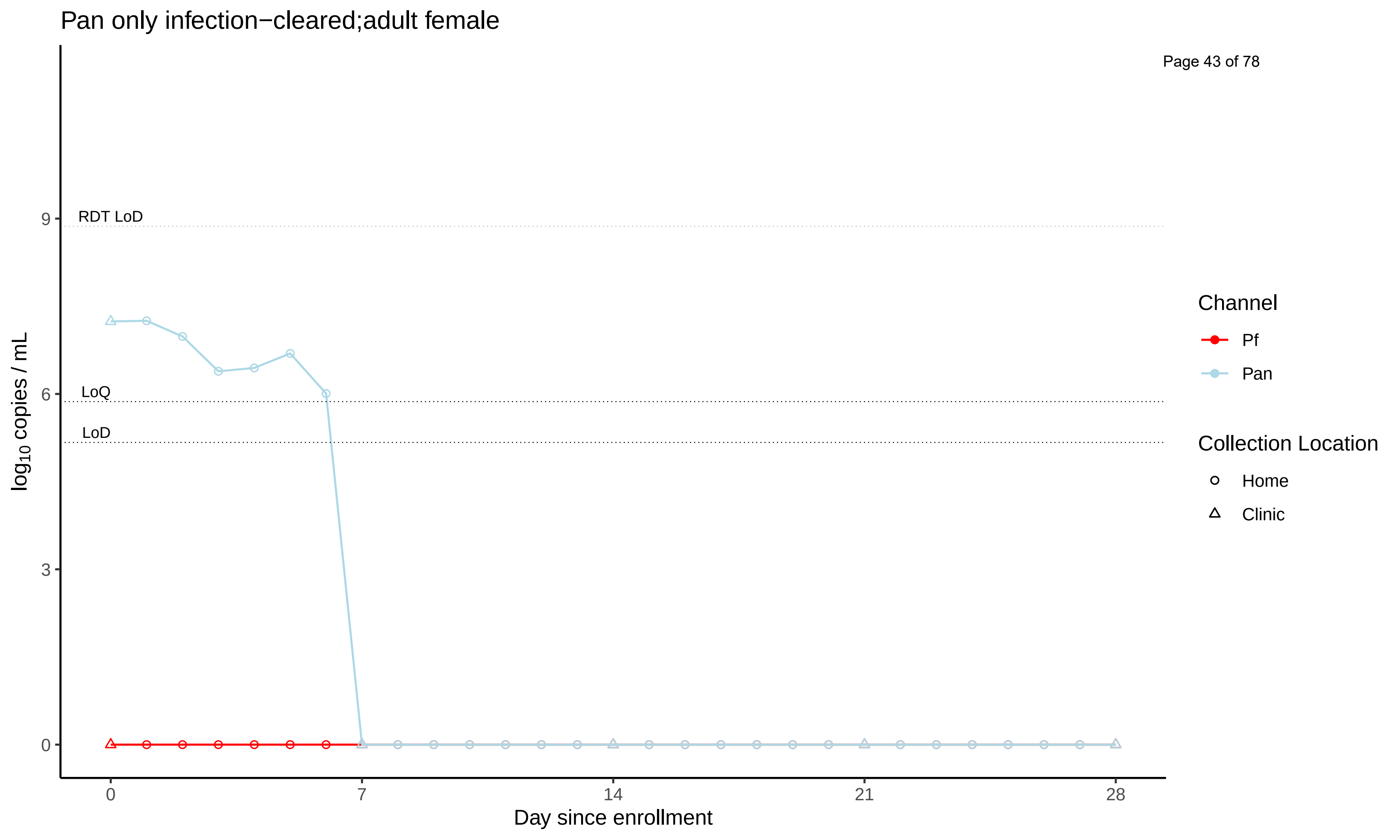
Pan only infection−cleared;adult female
RDT LoD
LoQ
LoD
log10 copies / mL
0
3
6
9
0
7
14
21
28
Day since enrollment
Channel
Pf
Pan
Collection Location
Home
Clinic

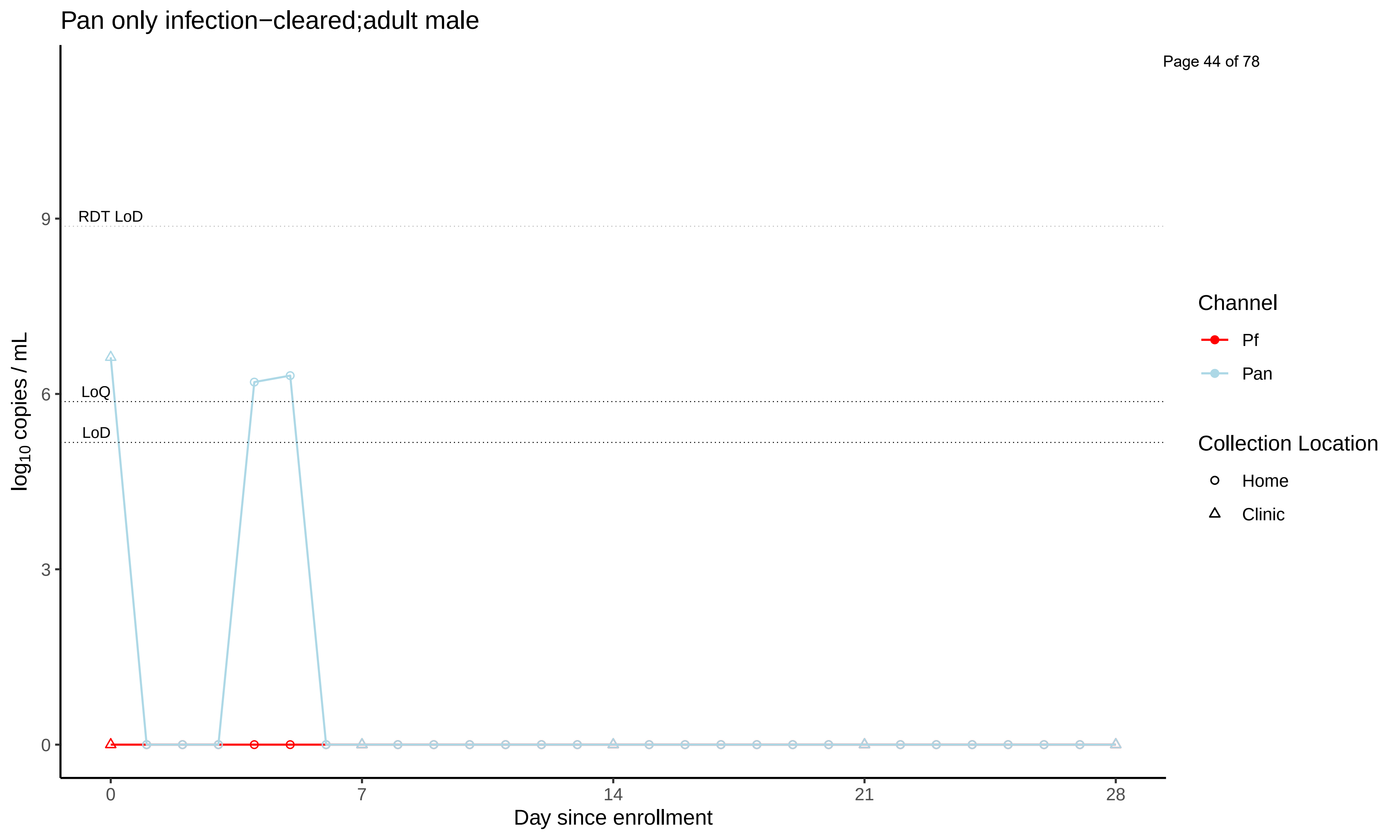
Pan only infection−cleared;adult male
Page 44 of 78
RDT LoD
LoQ
LoD
9
6
3
0
0
7
14
21
28
$\log_{10}$ copies / mL
Day since enrollment
Channel
Pf
Pan
Collection Location
Home
Clinic

Pan only infection−cleared;adult female

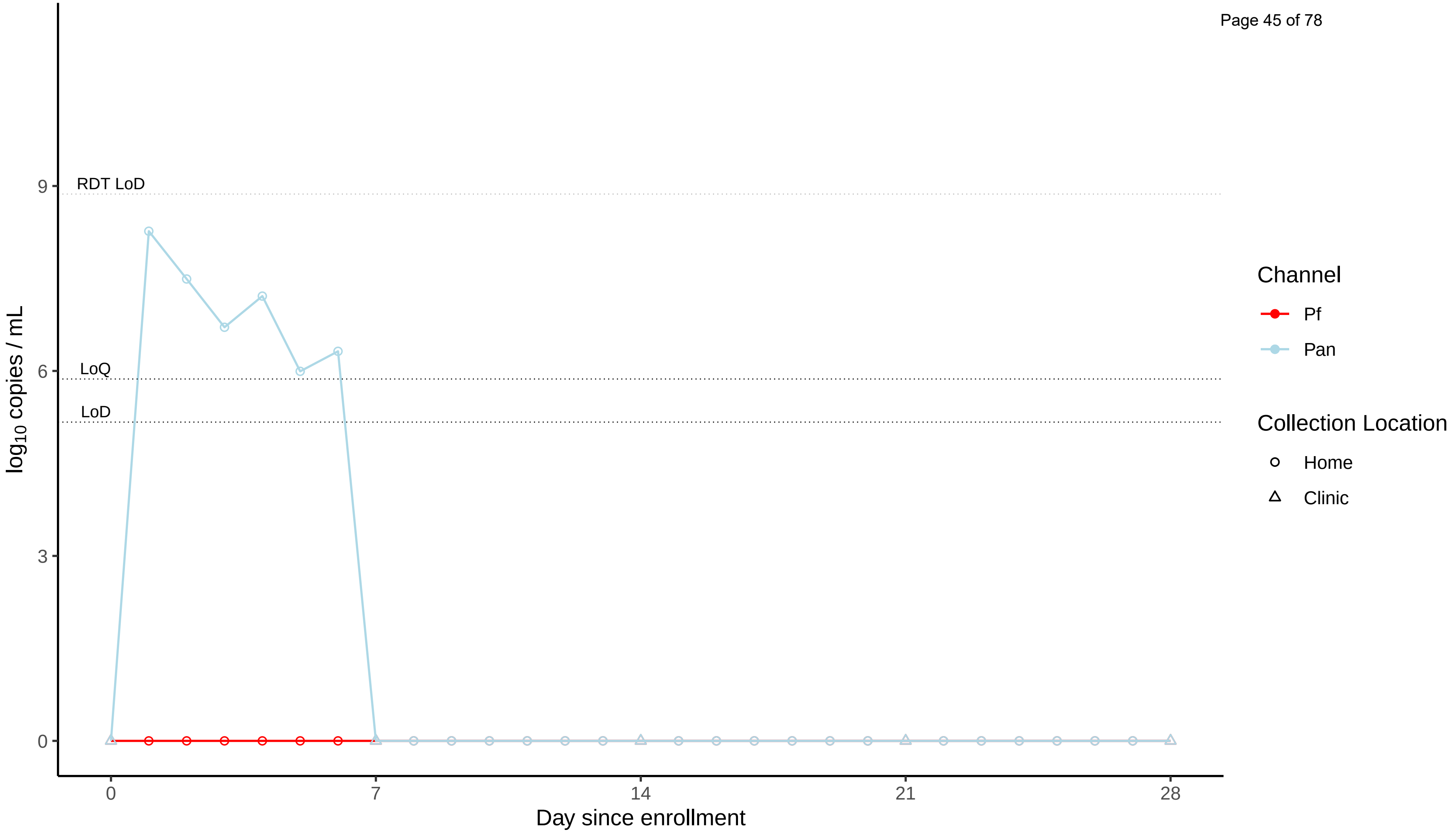

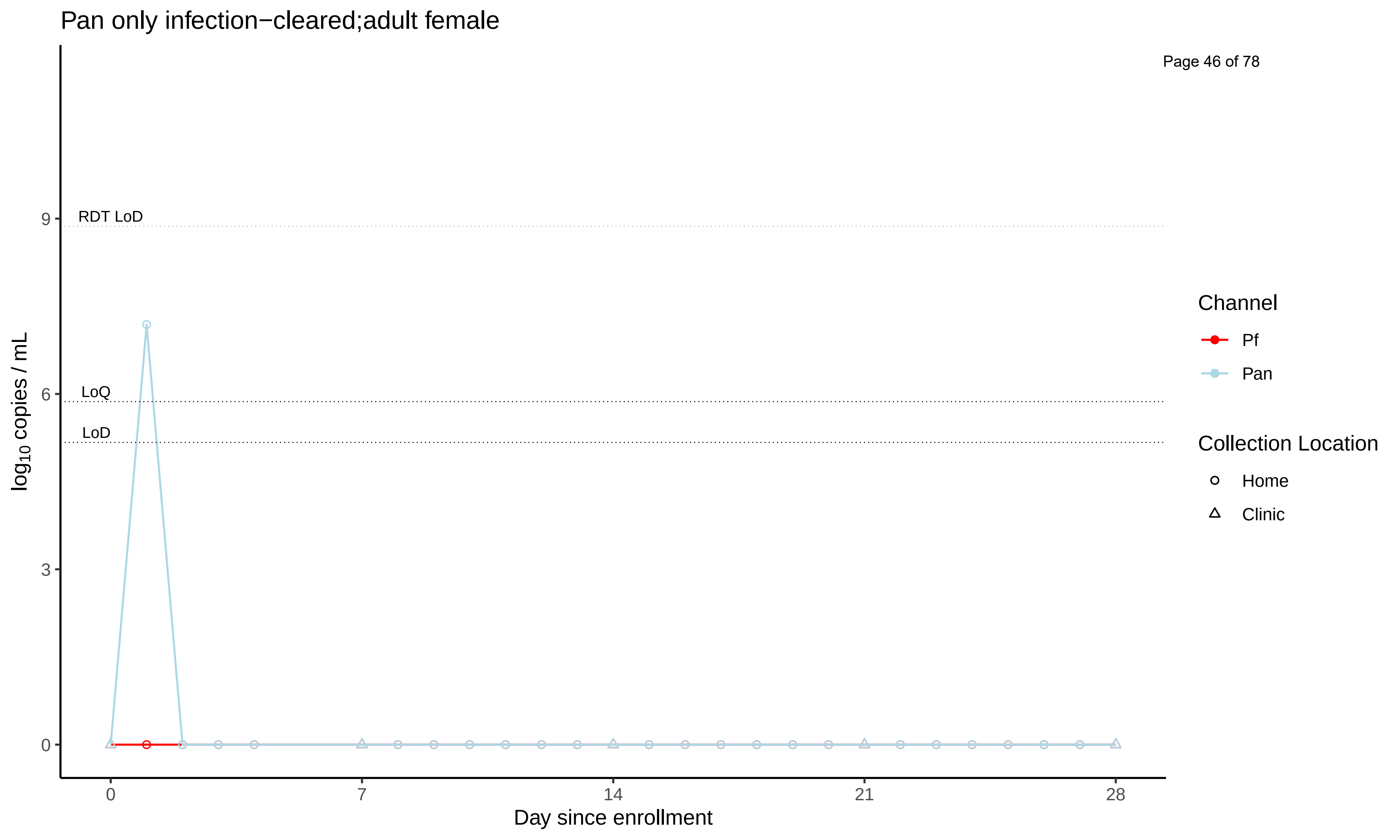
Pan only infection−cleared;adult female
Page 46 of 78
RDT LoD
LoQ
LoD
9
6
3
0
log_{10} copies / mL
0
7
14
21
28
Day since enrollment
Channel
Pf
Pan
Collection Location
Home
Clinic

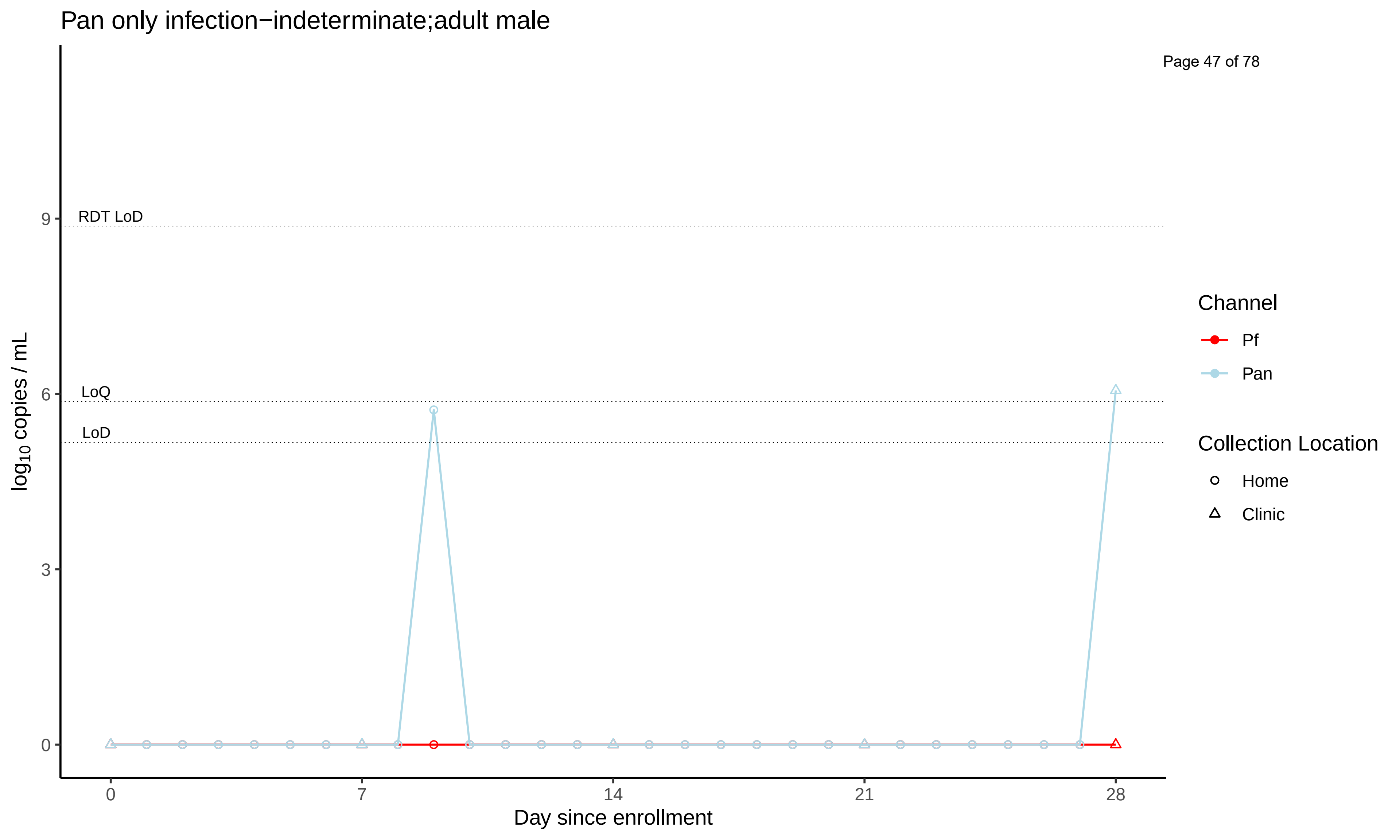
Pan only infection−indeterminate;adult male
RDT LoD
LoQ
LoD
9
6
3
0
$\log_{10}$ copies / mL
0
7
14
21
28
Day since enrollment
Channel
Pf
Pan
Collection Location
Home
Clinic

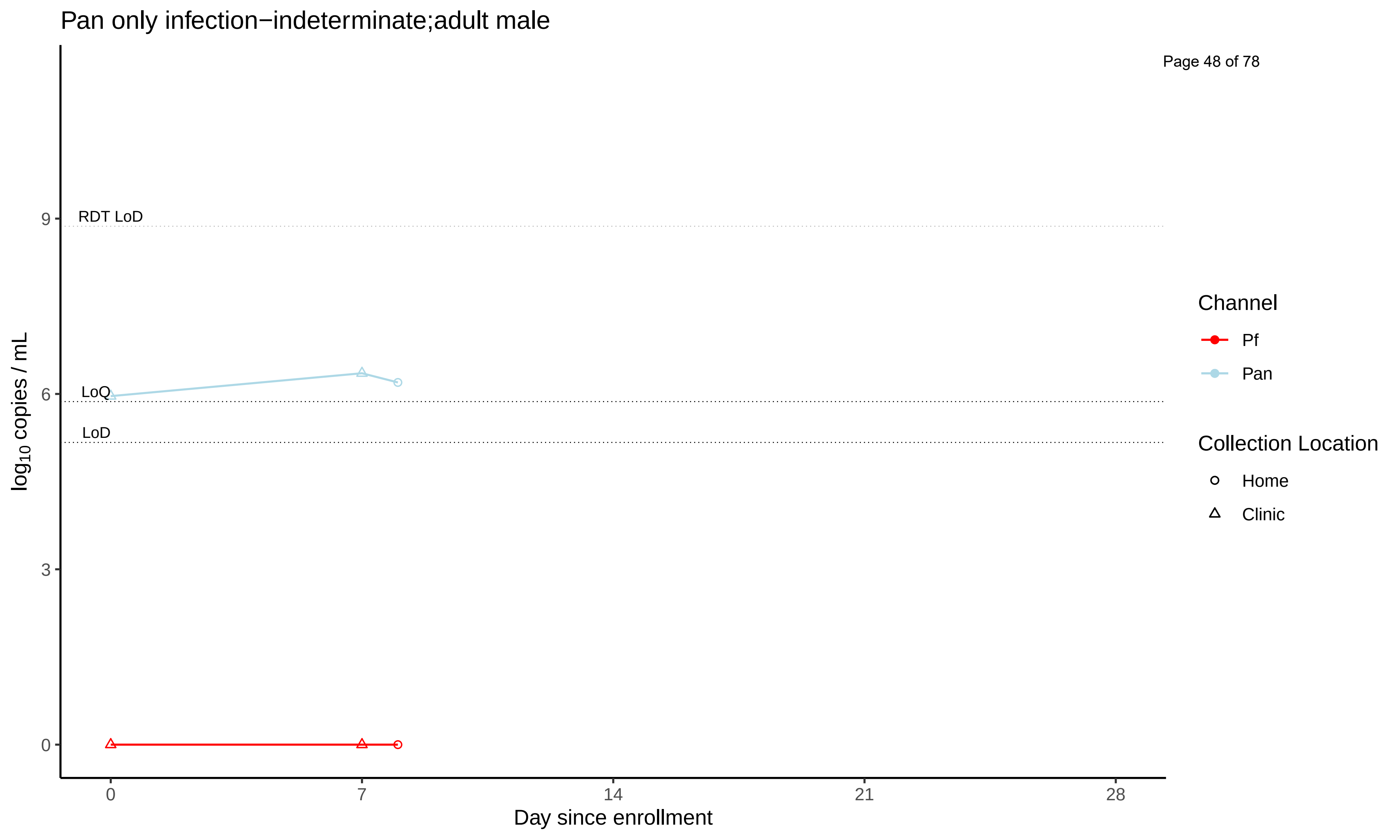
Pan only infection−indeterminate;adult male
RDT LoD
LoQ
LoD
9
6
3
0
0
7
14
21
28
Day since enrollment
log10 copies / mL
Channel
Pf
Pan
Collection Location
Home
Clinic

Pan only infection−indeterminate;adult male

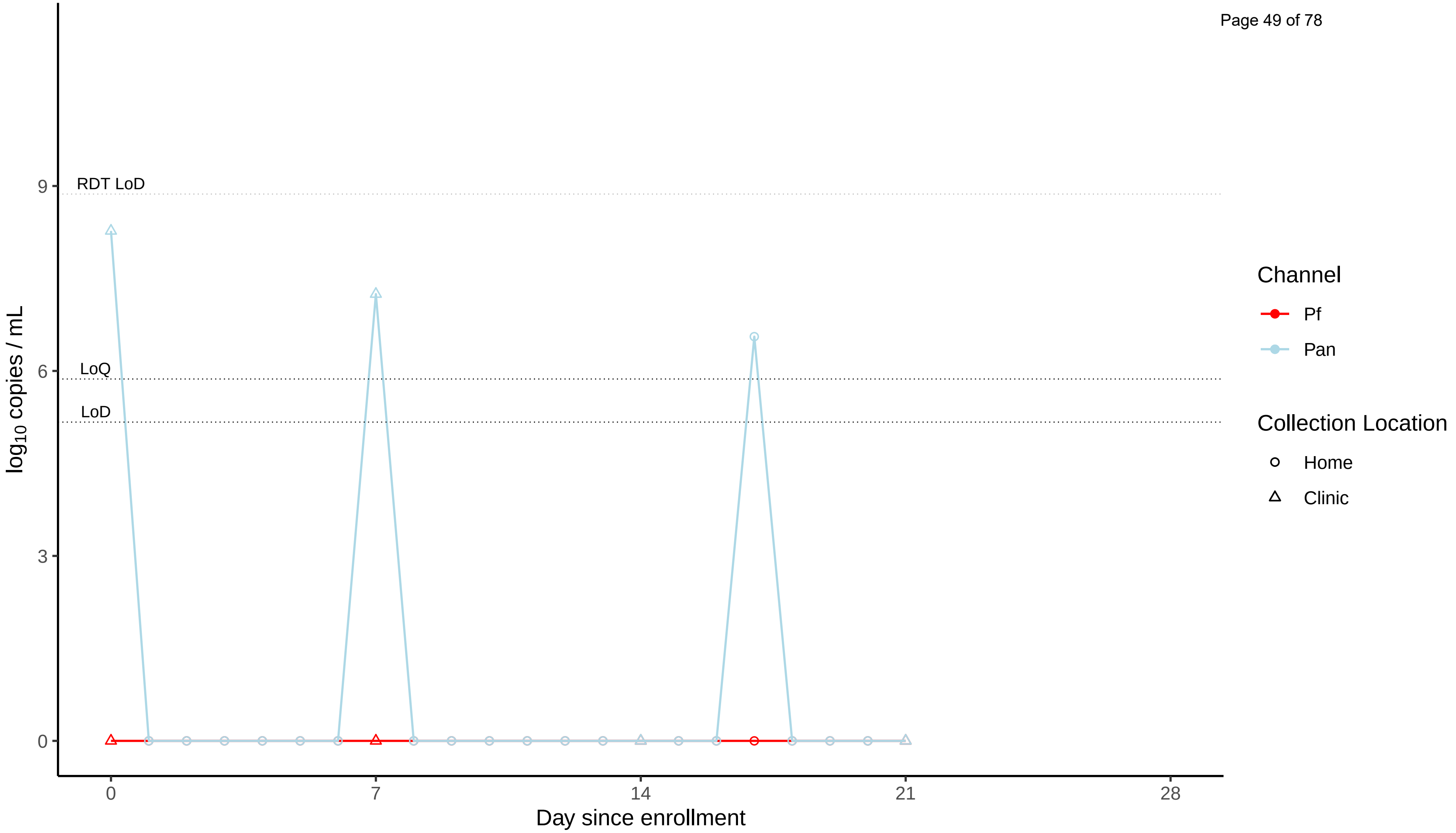

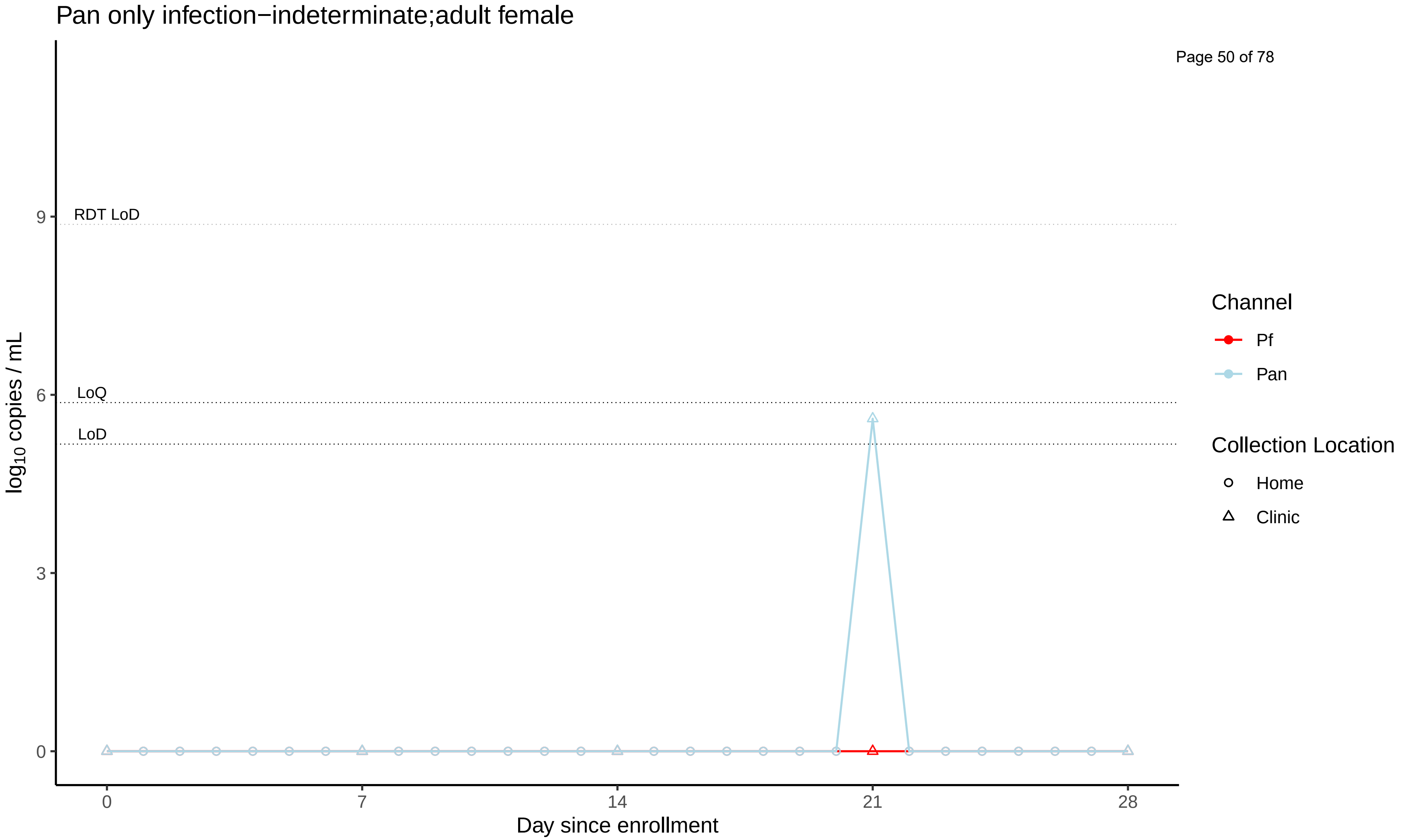
Pan only infection−indeterminate;adult female
RDT LoD
LoQ
LoD
9
6
3
0
$\log_{10}$ copies / mL
0
7
14
21
28
Day since enrollment
Channel
Pf
Pan
Collection Location
Home
Clinic

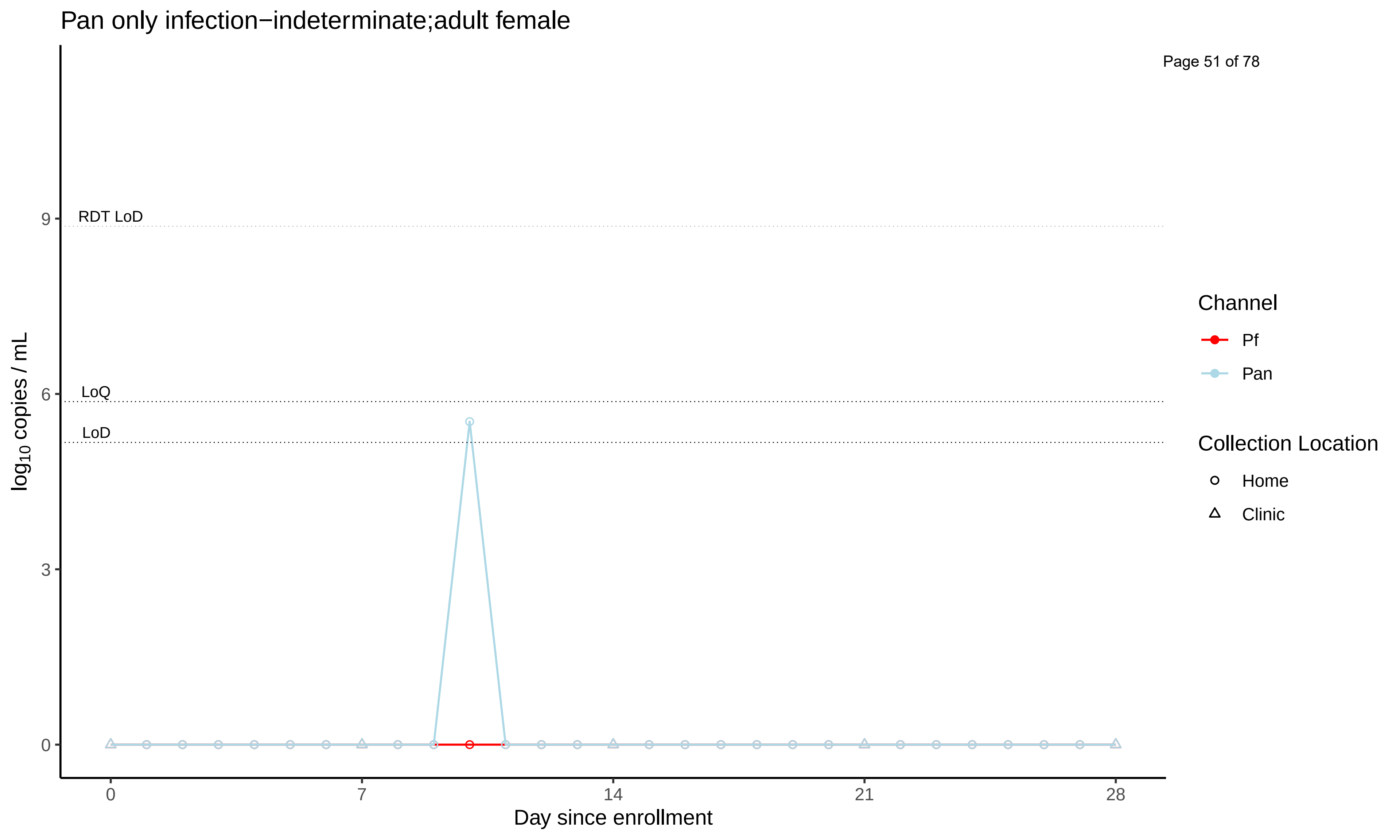
Pan only infection−indeterminate;adult female
RDT LoD
LoQ
LoD
9
6
3
0
log10 copies / mL
0
7
14
21
28
Day since enrollment
Channel
Pf
Pan
Collection Location
Home
Clinic

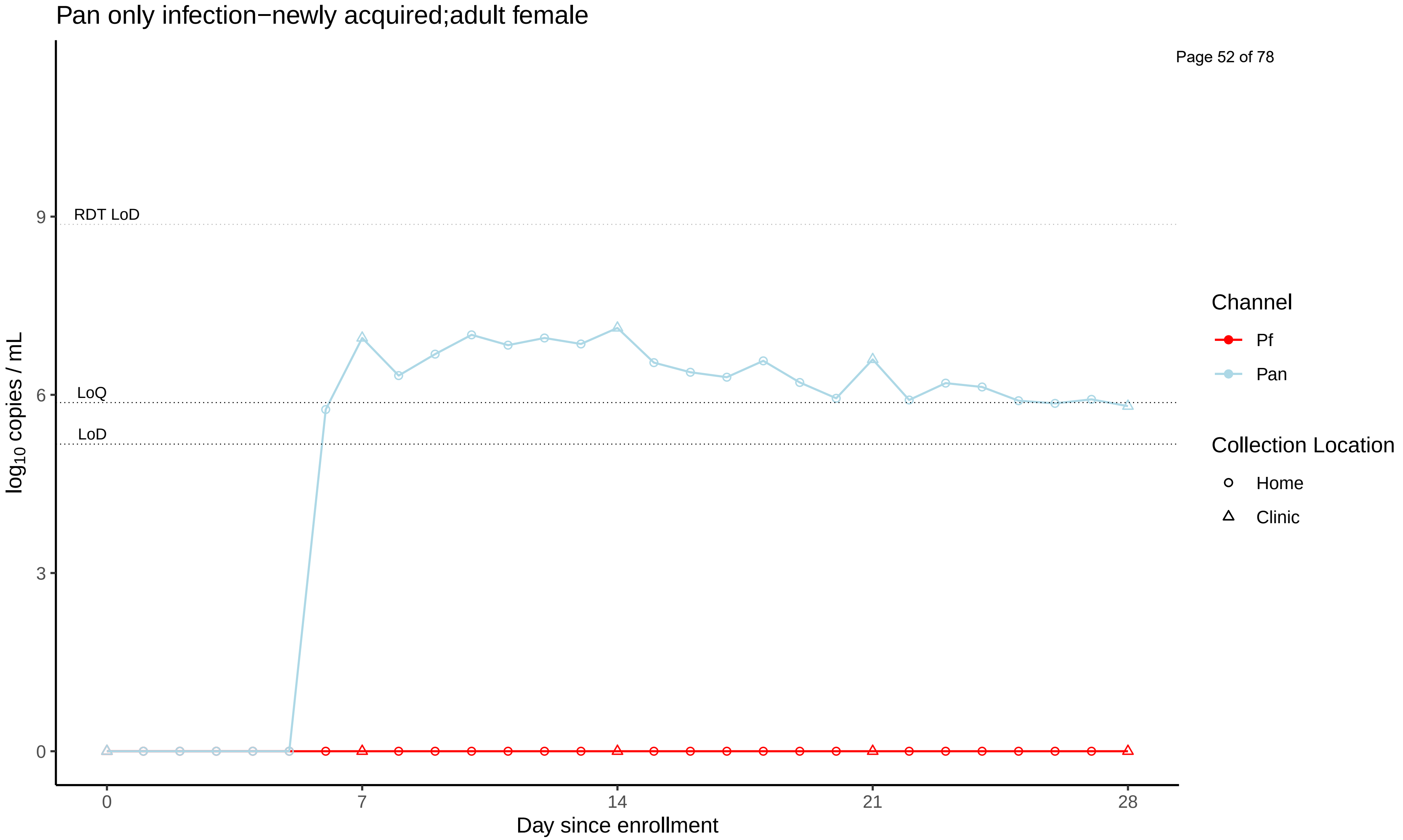
Pan only infection−newly acquired;adult female
RDT LoD
LoQ
LoD
9
6
3
0
0
7
14
21
28
Day since enrollment
log10 copies / mL
Channel
Pf
Pan
Collection Location
Home
Clinic

Pan only infection−newly acquired;adult male

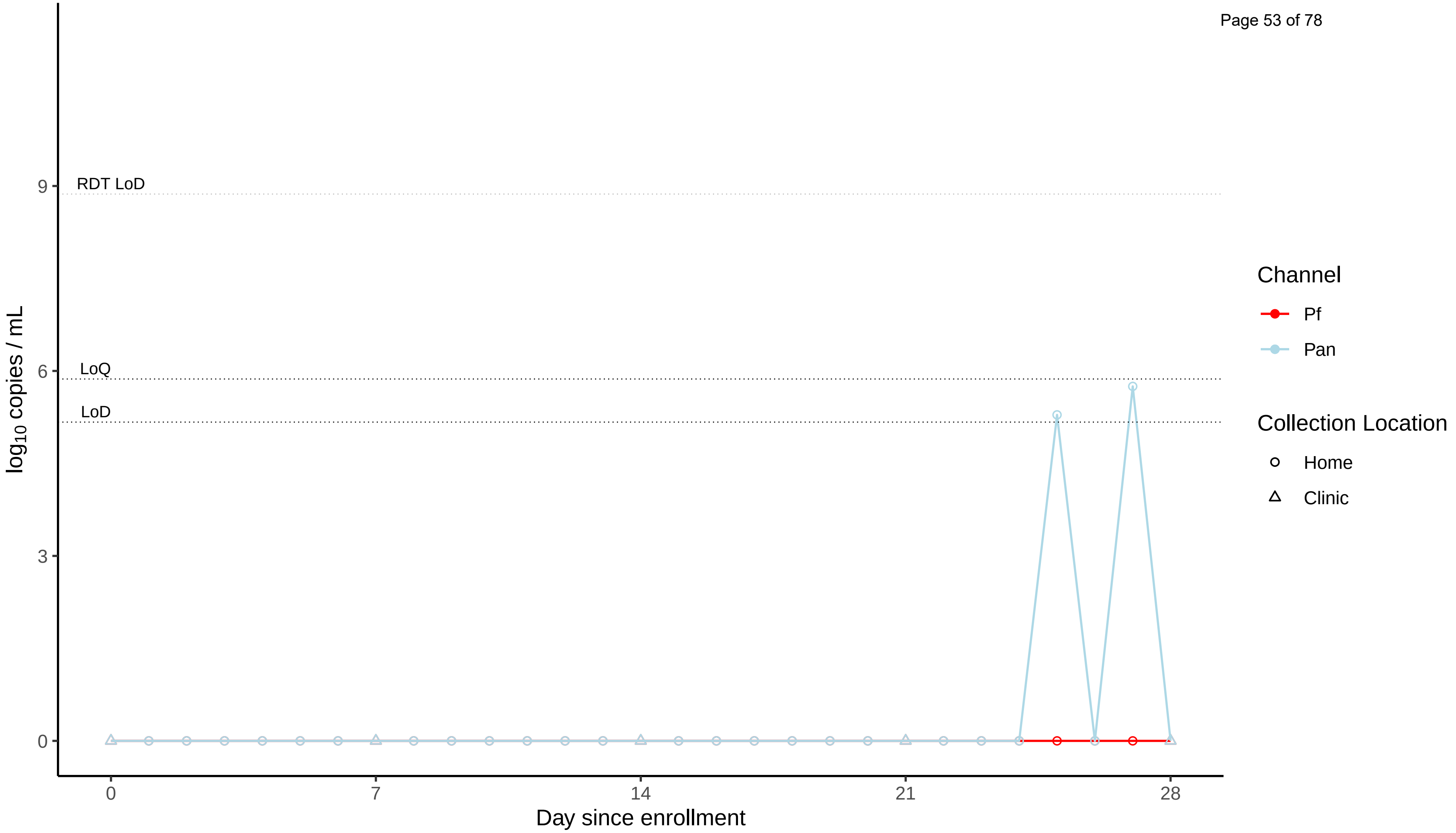

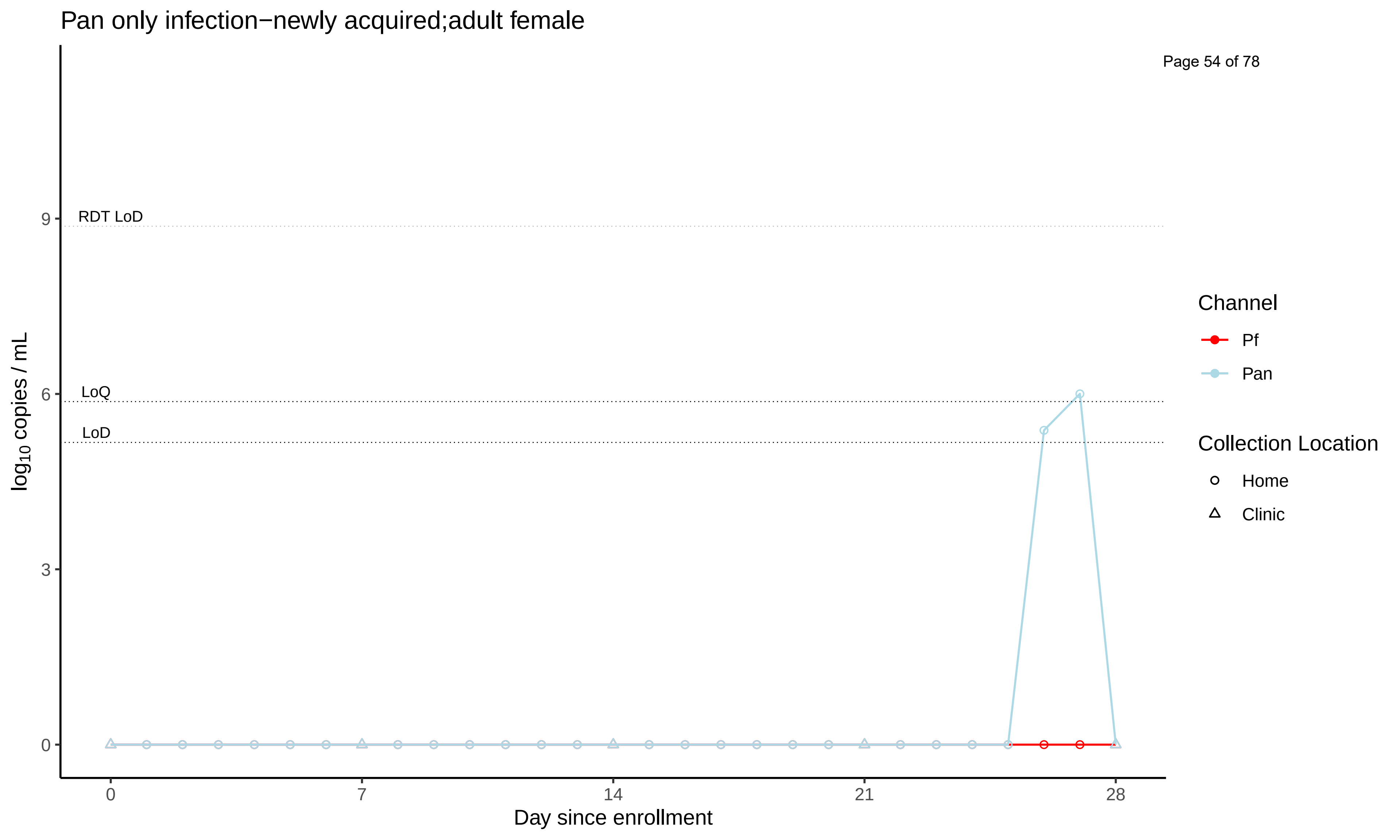

Pan only infection−newly acquired;adult female
Page 54 of 78
RDT LoD
LoQ
LoD
log10 copies / mL
9
6
3
0
0
7
14
21
28
Day since enrollment
Channel
Pf
Pan
Collection Location
Home
Clinic

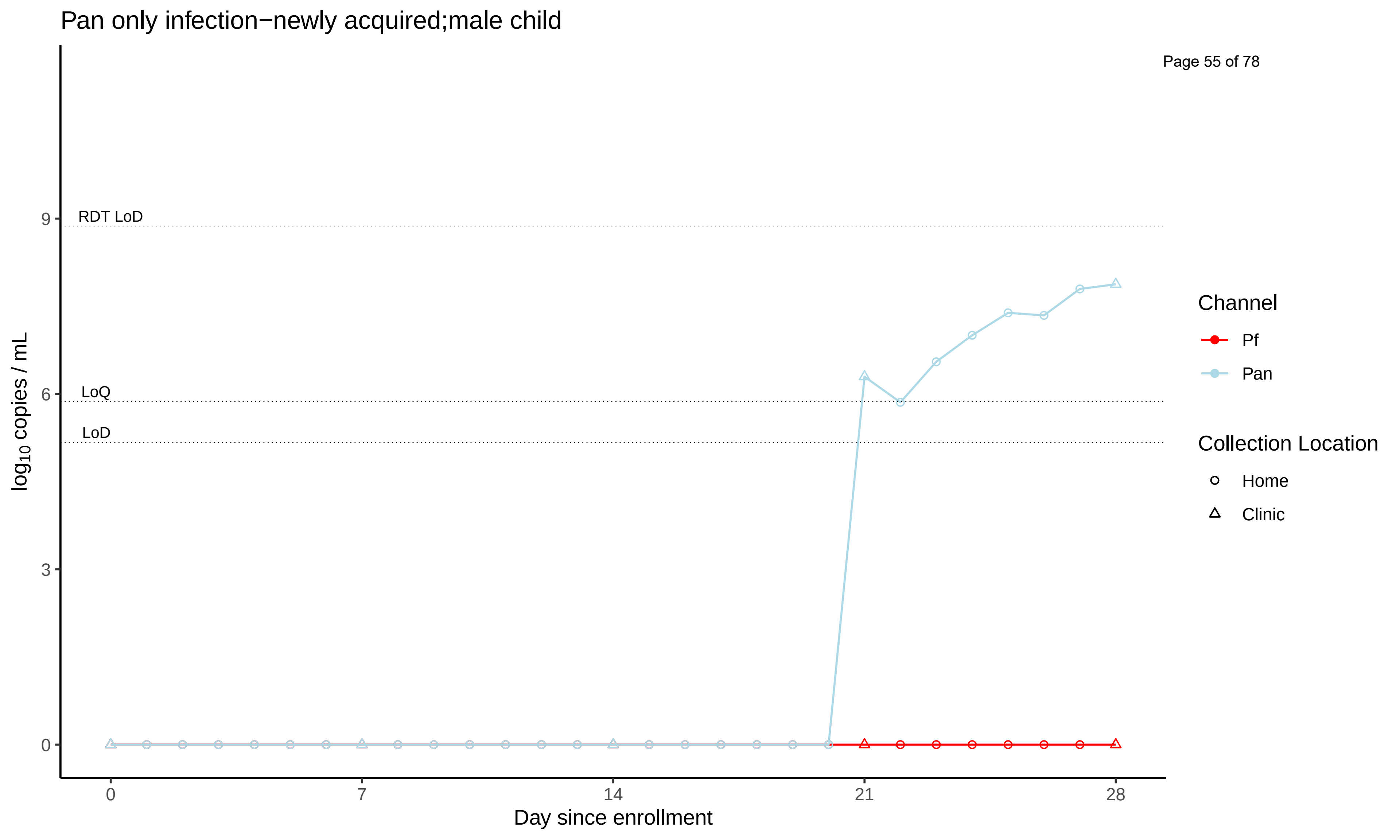
Pan only infection−newly acquired;male child
RDT LoD
LoQ
LoD
9
6
3
0
log10 copies / mL
0
7
14
21
28
Day since enrollment
Channel
Pf
Pan
Collection Location
Home
Clinic

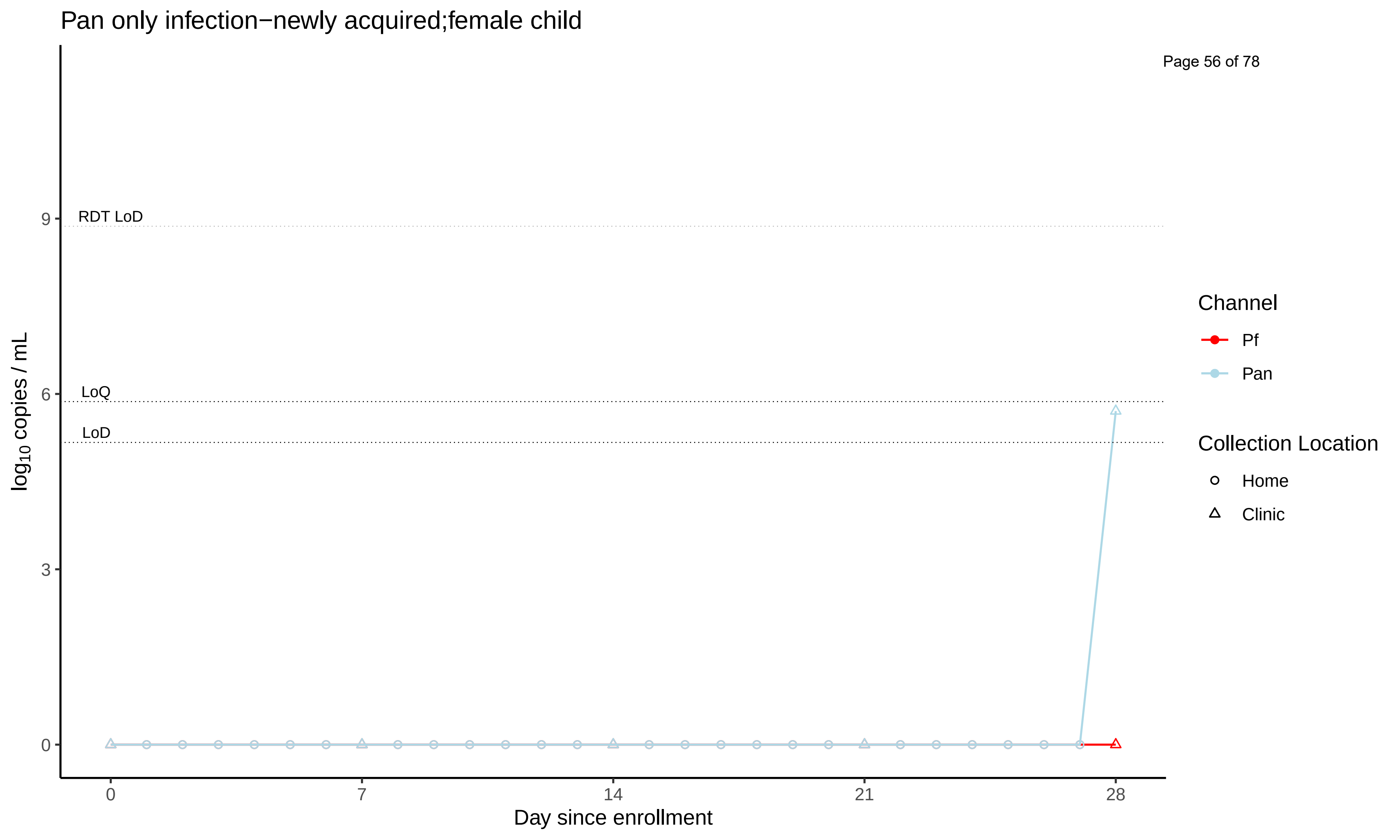
Pan only infection−newly acquired;female child
RDT LoD
LoQ
LoD
9
6
3
0
0
7
14
21
28
log10 copies / mL
Day since enrollment
Channel
Pf
Pan
Collection Location
Home
Clinic

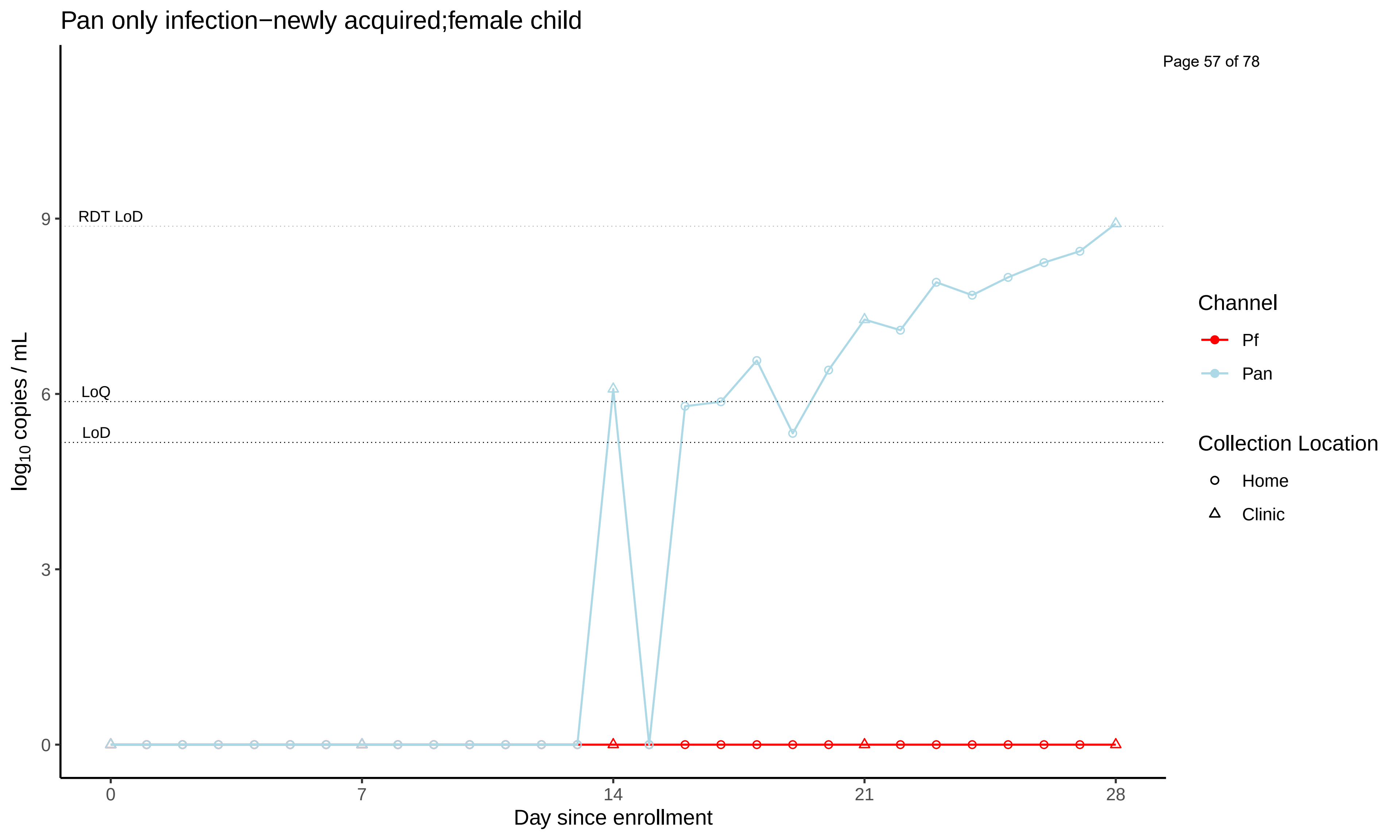
Pan only infection−newly acquired;female child
Page 57 of 78
RDT LoD
LoQ
LoD
9
6
3
0
$\log_{10}$ copies / mL
0
7
14
21
28
Day since enrollment
Channel
Pf
Pan
Collection Location
Home
Clinic

Pan only infection−newly acquired;male child

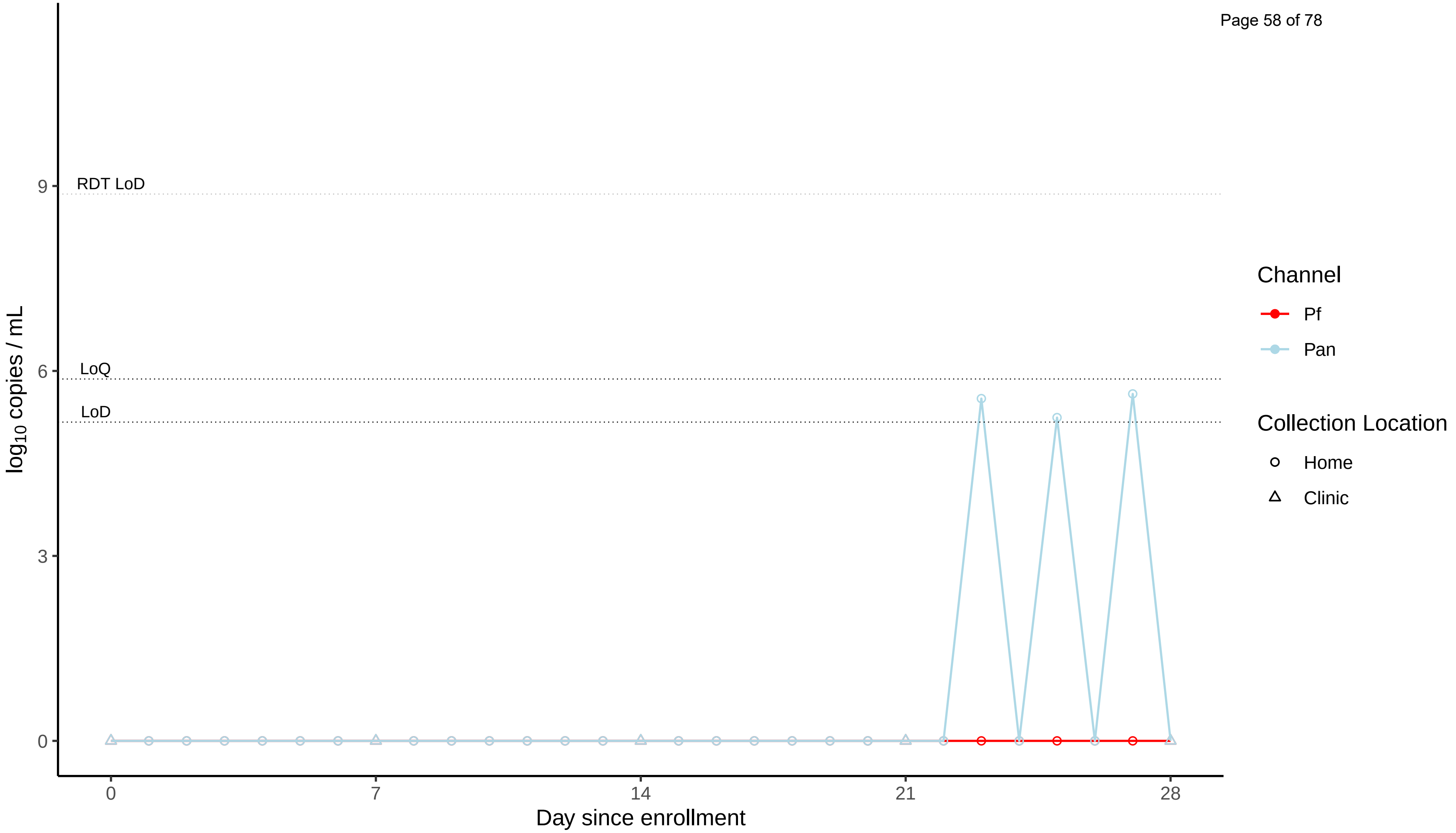

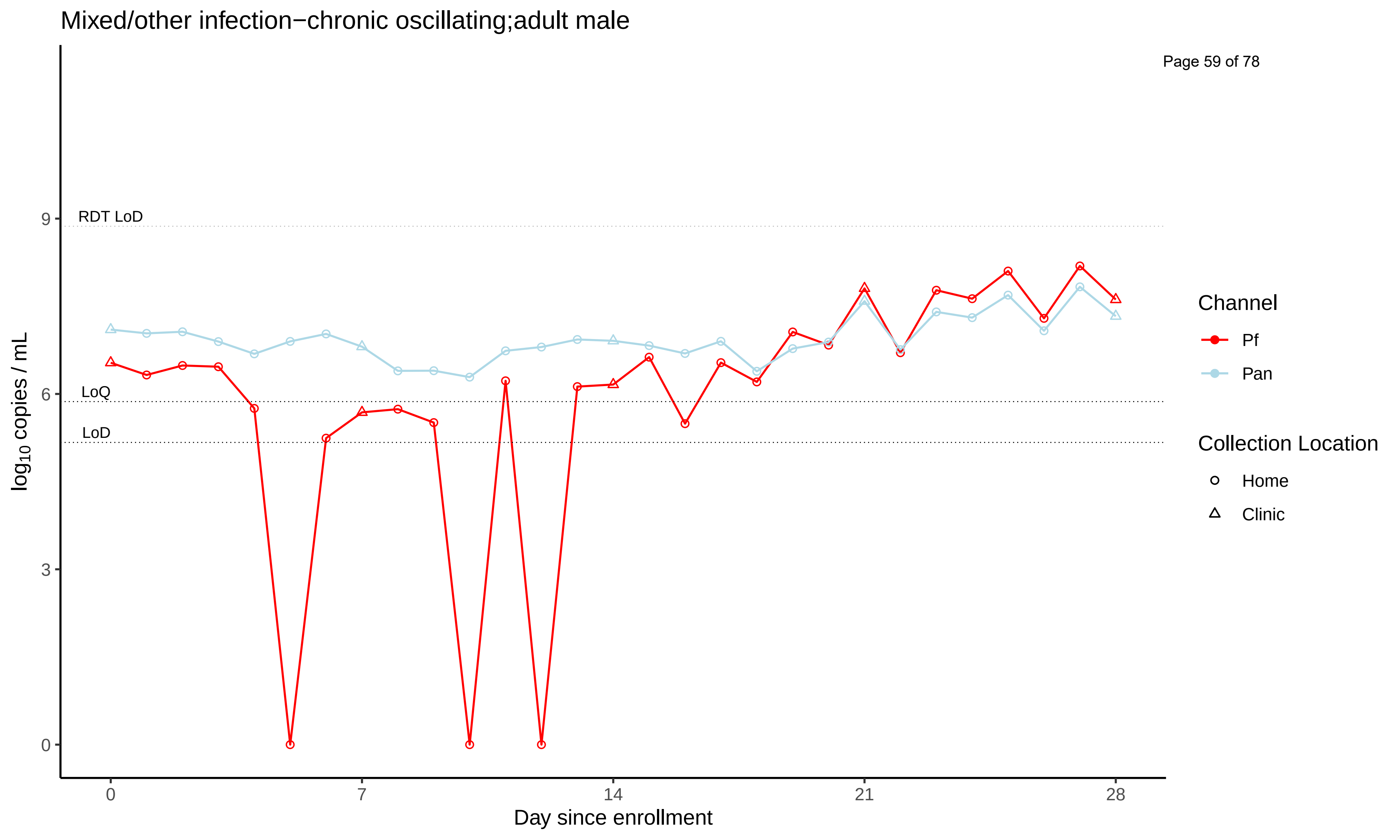
Mixed/other infection−chronic oscillating;adult male
RDT LoD
LoQ
LoD
9
6
3
0
0
7
14
21
28
log_{10} copies / mL
Day since enrollment
Channel
Pf
Pan
Collection Location
Home
Clinic

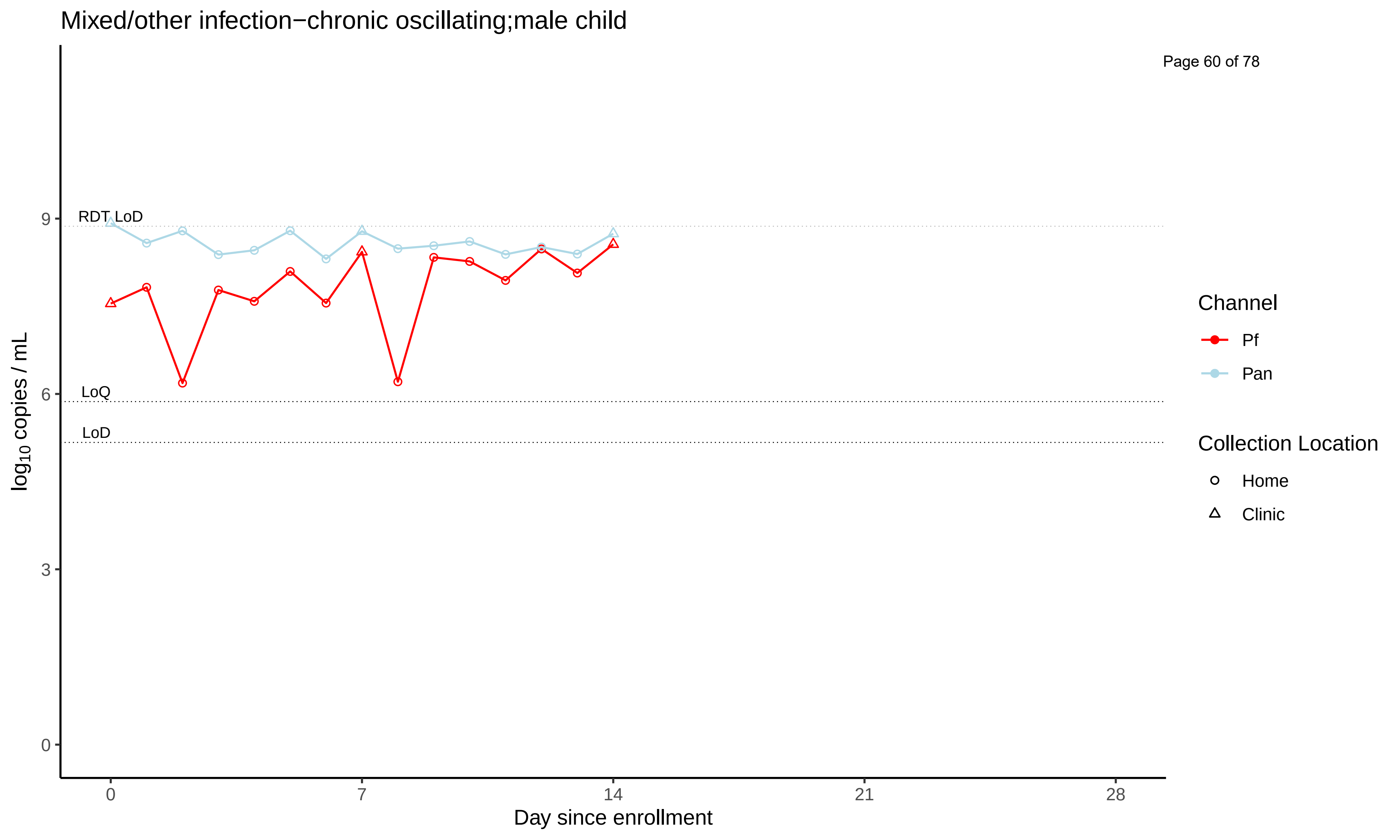
Mixed/other infection−chronic oscillating;male child
RDT LoD
LoQ
LoD
9
6
3
0
$\log_{10}$ copies / mL
0
7
14
21
28
Day since enrollment
Channel
Pf
Pan
Collection Location
Home
Clinic

Mixed/other infection−chronic oscillating;adult male

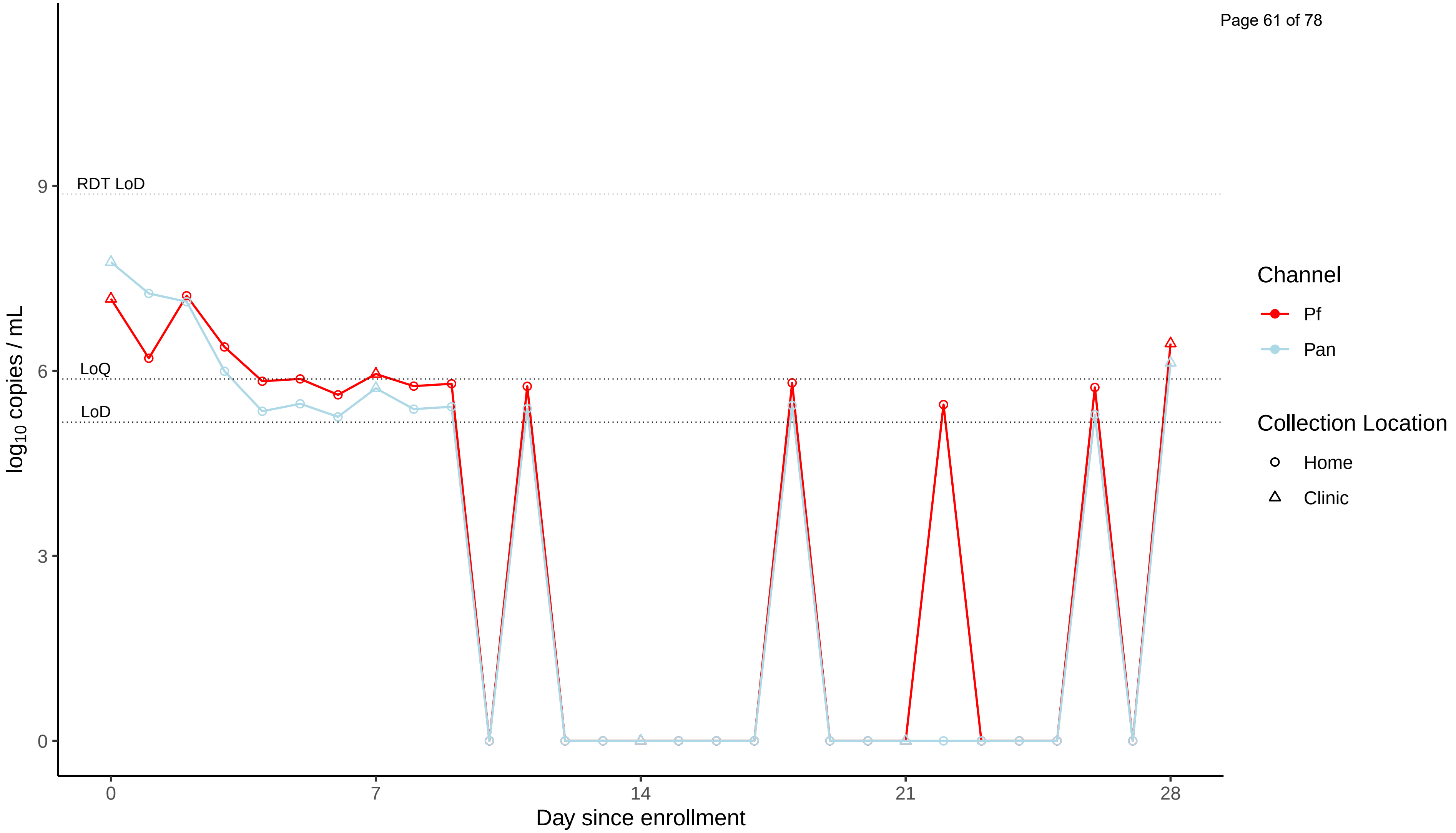

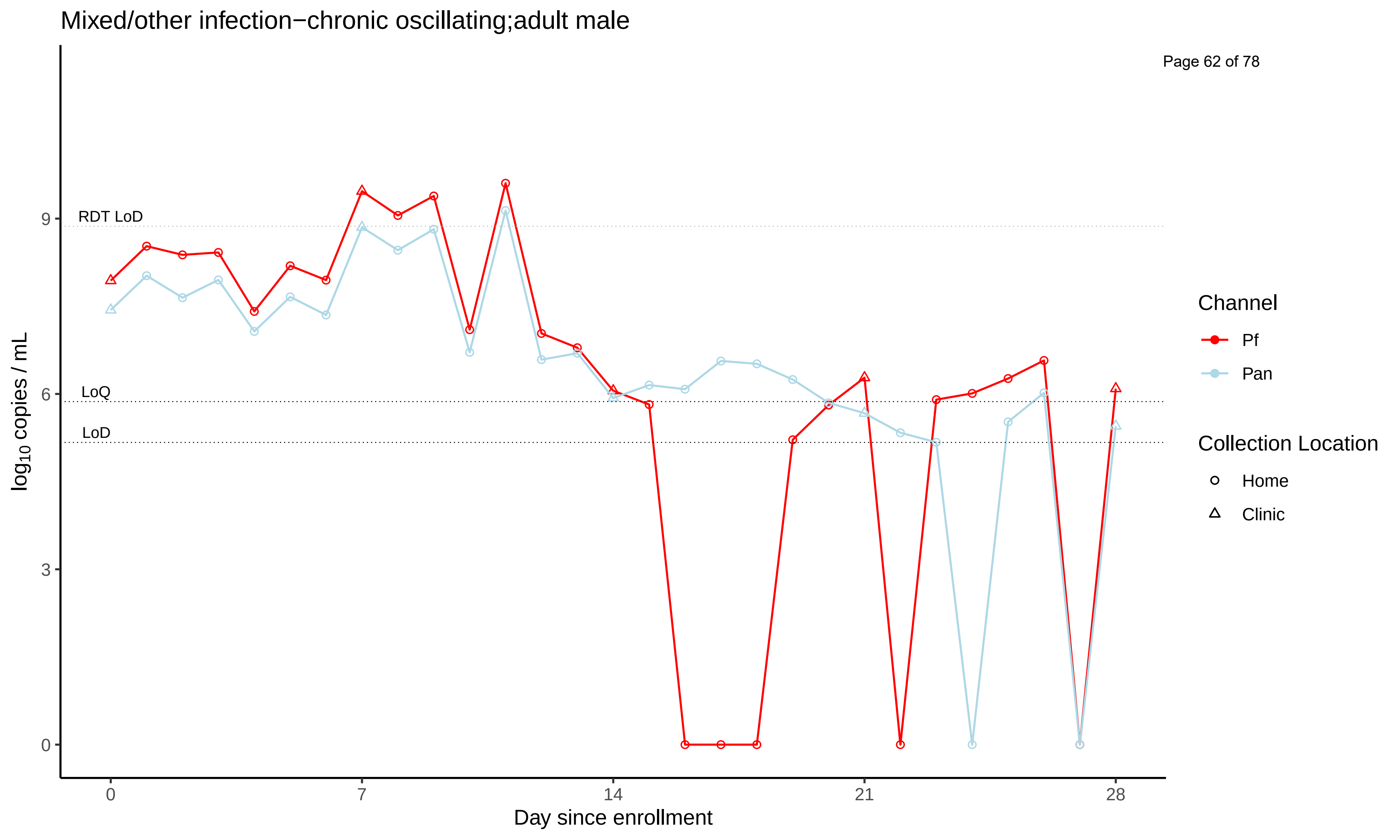
Mixed/other infection−chronic oscillating;adult male
RDT LoD
LoQ
LoD
9
6
3
0
log10 copies / mL
0
7
14
21
28
Day since enrollment
Channel
Pf
Pan
Collection Location
Home
Clinic

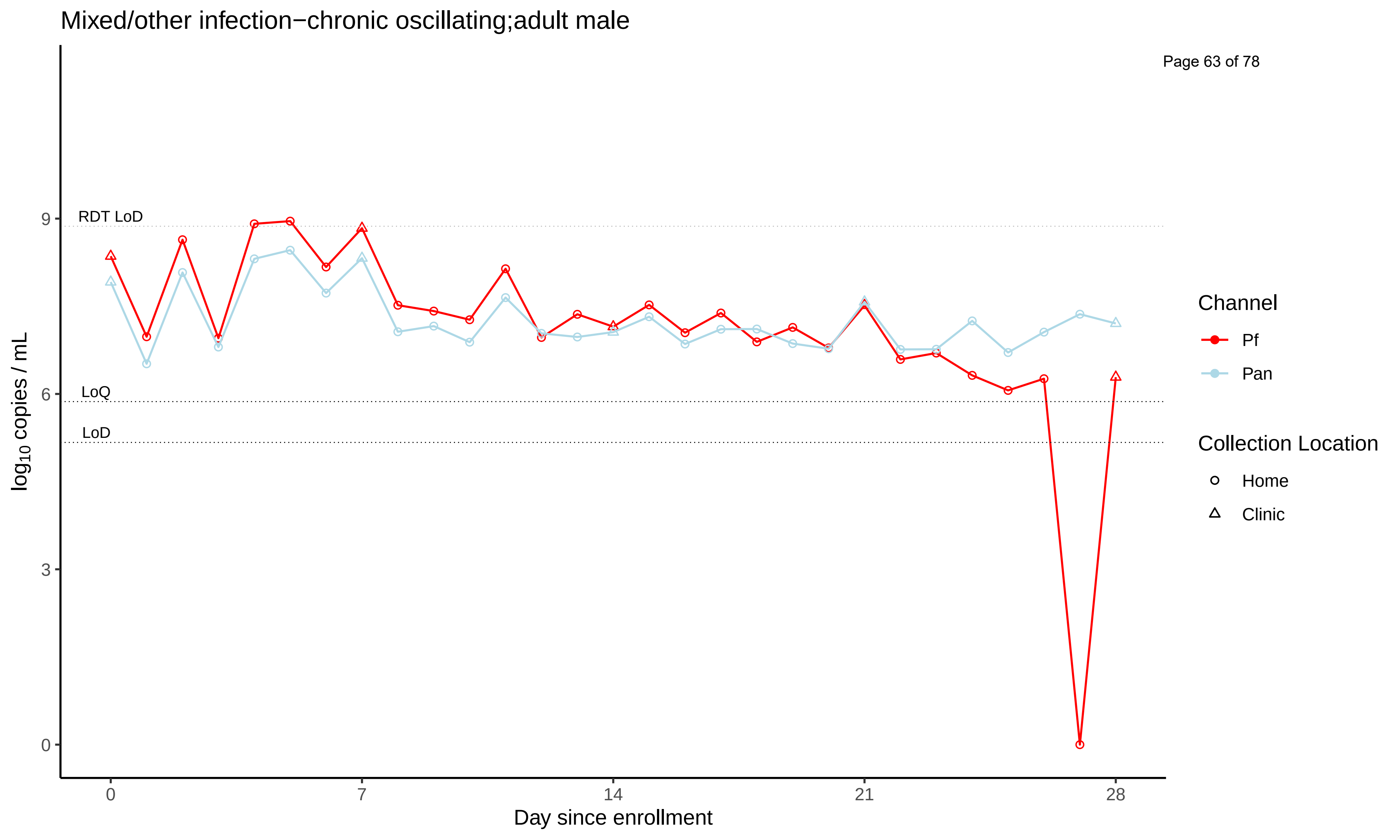
Mixed/other infection−chronic oscillating;adult male
RDT LoD
LoQ
LoD
9
6
3
0
$\log_{10}$ copies / mL
0
7
14
21
28
Day since enrollment
Channel
Pf
Pan
Collection Location
Home
Clinic

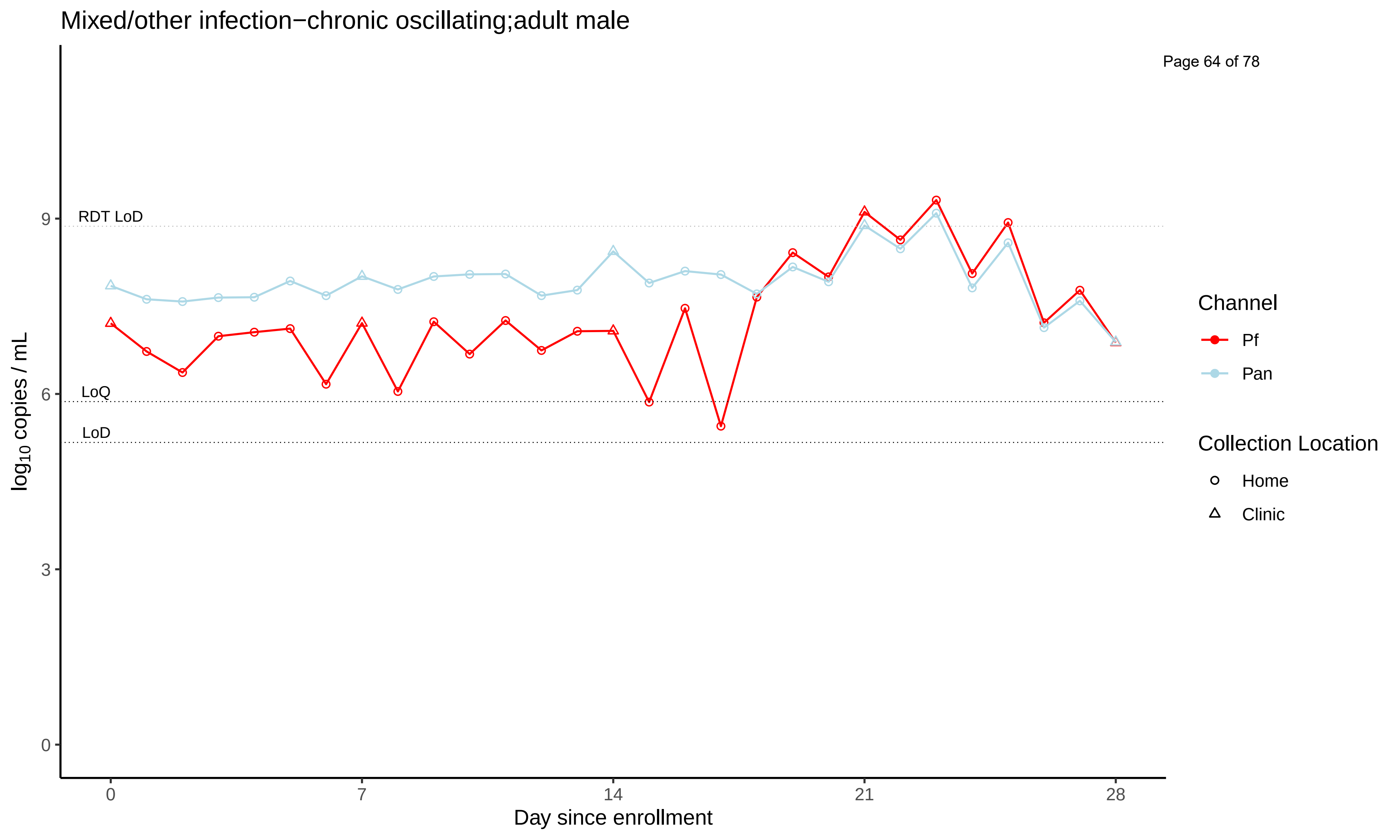
Mixed/other infection−chronic oscillating;adult male
RDT LoD
LoQ
LoD
9
6
3
0
0
7
14
21
28
Day since enrollment
log10 copies / mL
Channel
Pf
Pan
Collection Location
Home
Clinic

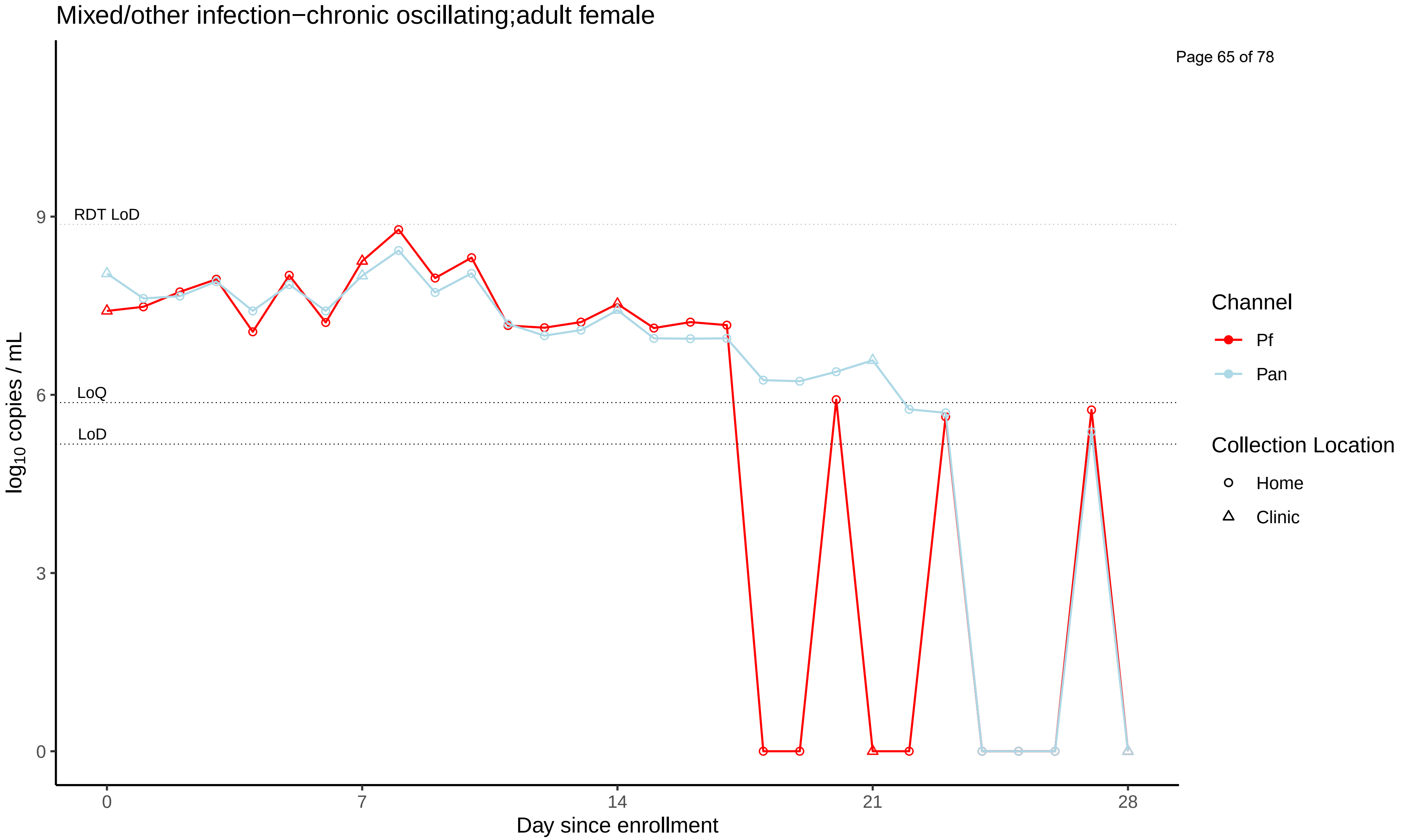
Mixed/other infection−chronic oscillating;adult female
log10 copies / mL
9
6
3
0
RDT LoD
LoQ
LoD
0
7
14
21
28
Day since enrollment
Channel
Pf
Pan
Collection Location
Home
Clinic

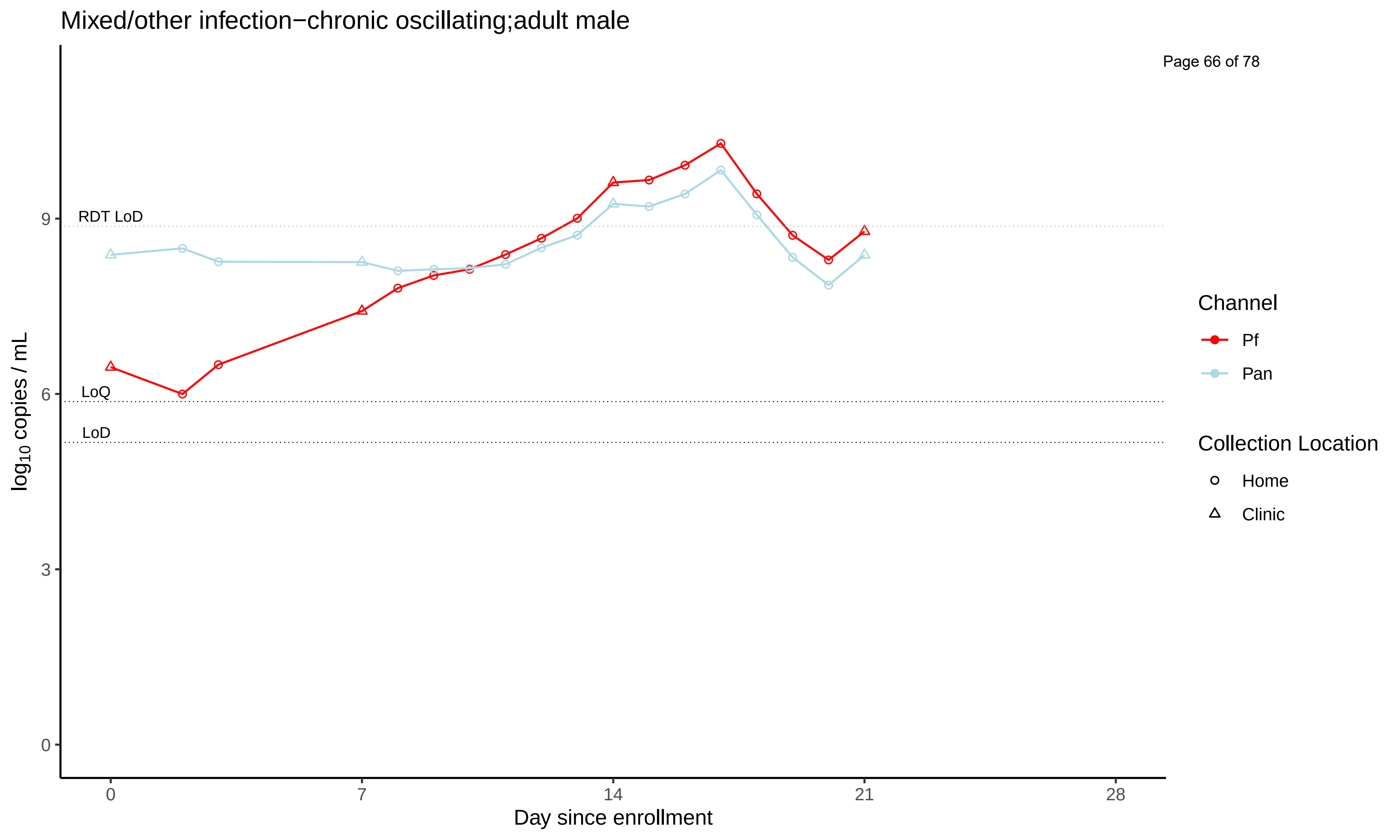
Mixed/other infection−chronic oscillating;adult male
RDT LoD
LoQ
LoD
log_{10} copies / mL
9
6
3
0
0
7
14
21
28
Day since enrollment
Channel
Pf
Pan
Collection Location
Home
Clinic

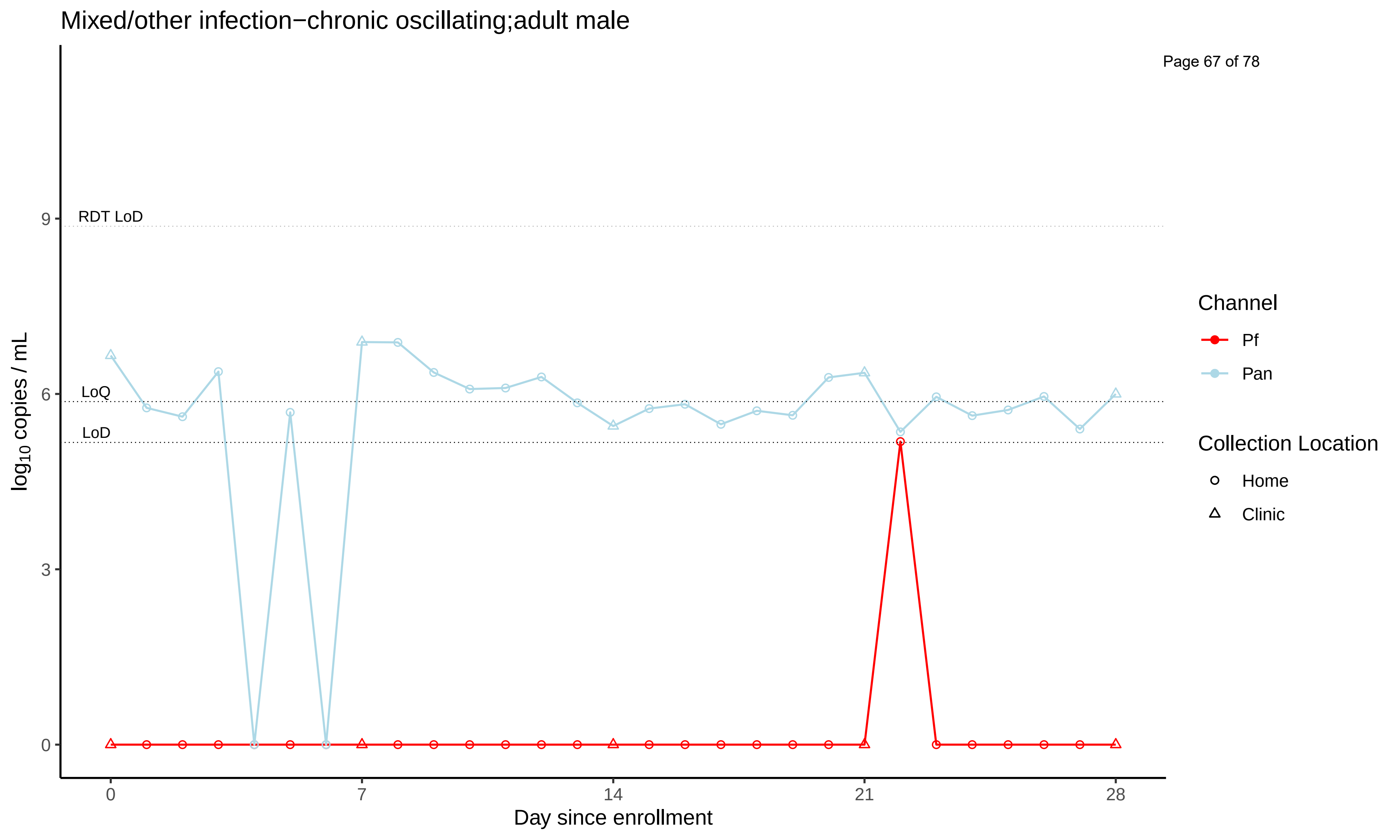
Mixed/other infection−chronic oscillating;adult male
RDT LoD
LoQ
LoD
9
6
3
0
log_{10} copies / mL
0
7
14
21
28
Day since enrollment
Channel
Pf
Pan
Collection Location
Home
Clinic

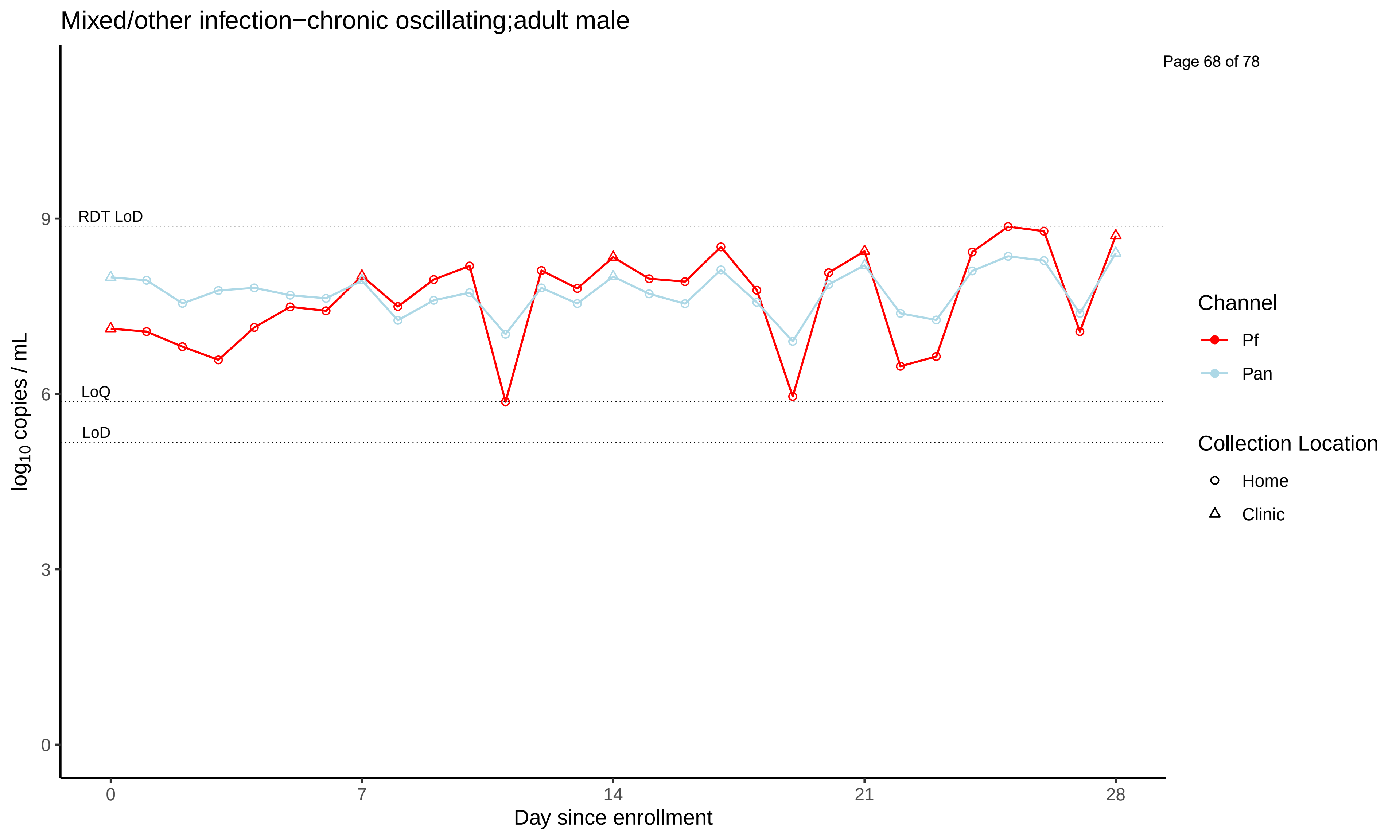
Mixed/other infection−chronic oscillating;adult male
RDT LoD
LoQ
LoD
$\log_{10}$ copies / mL
0
3
6
9
0
7
14
21
28
Day since enrollment
Channel
Pf
Pan
Collection Location
Home
Clinic

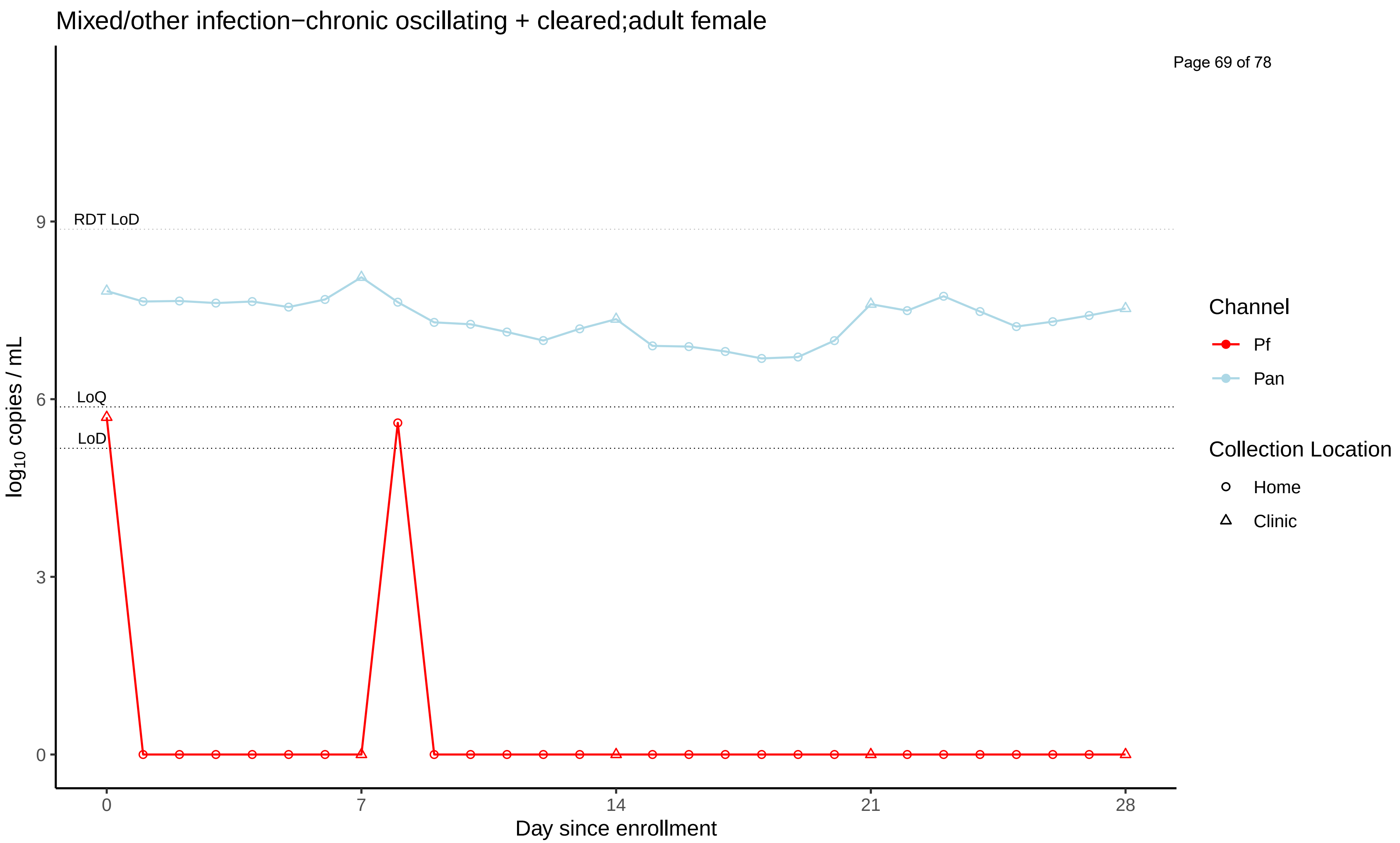
Mixed/other infection−chronic oscillating + cleared;adult female
RDT LoD
LoQ
LoD
$\log_{10}$ copies / mL
9
6
3
0
0
7
14
21
28
Day since enrollment
Channel
Pf
Pan
Collection Location
Home
Clinic

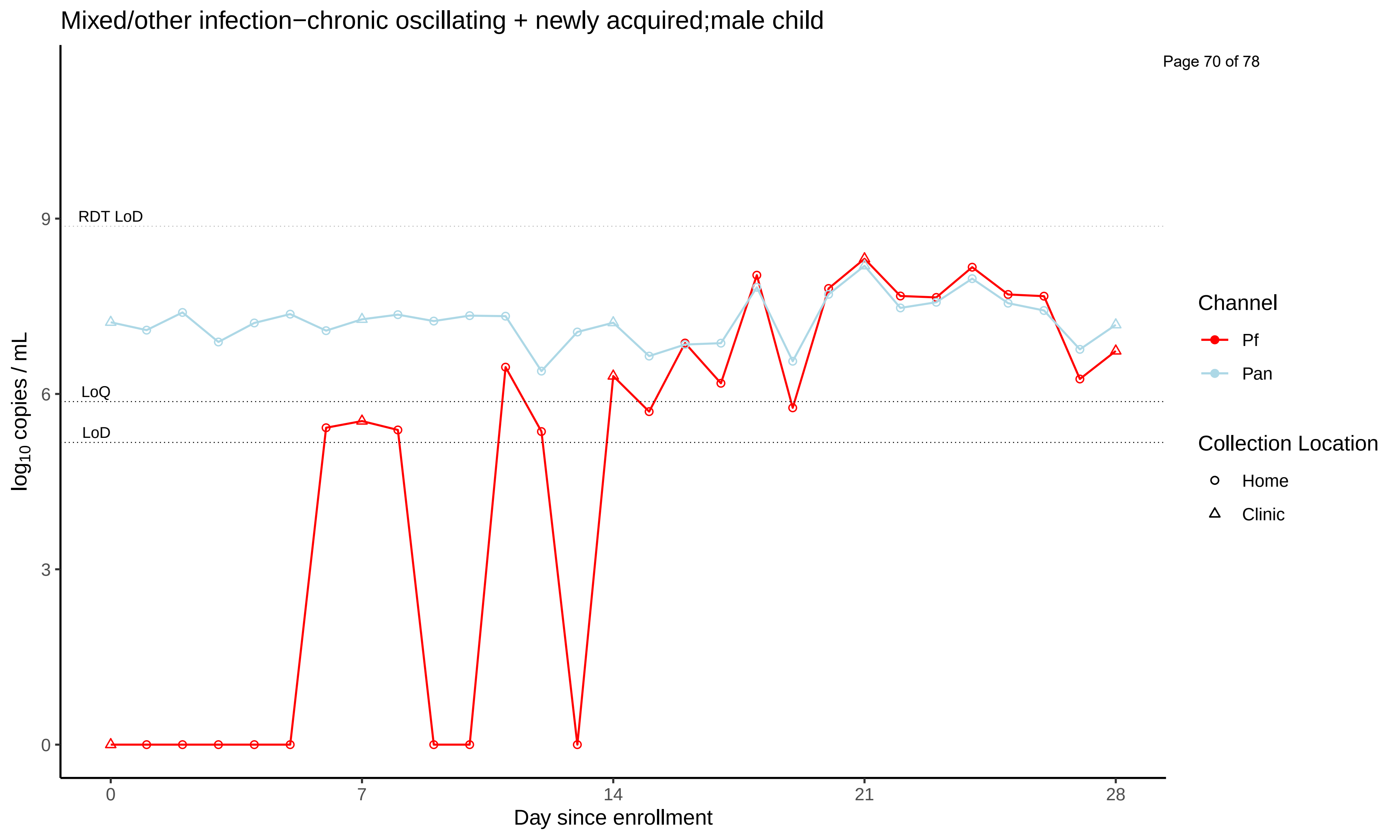
Mixed/other infection−chronic oscillating + newly acquired;male child
RDT LoD
LoQ
LoD
log10 copies / mL
0
3
6
9
0
7
14
21
28
Day since enrollment
Channel
Pf
Pan
Collection Location
Home
Clinic

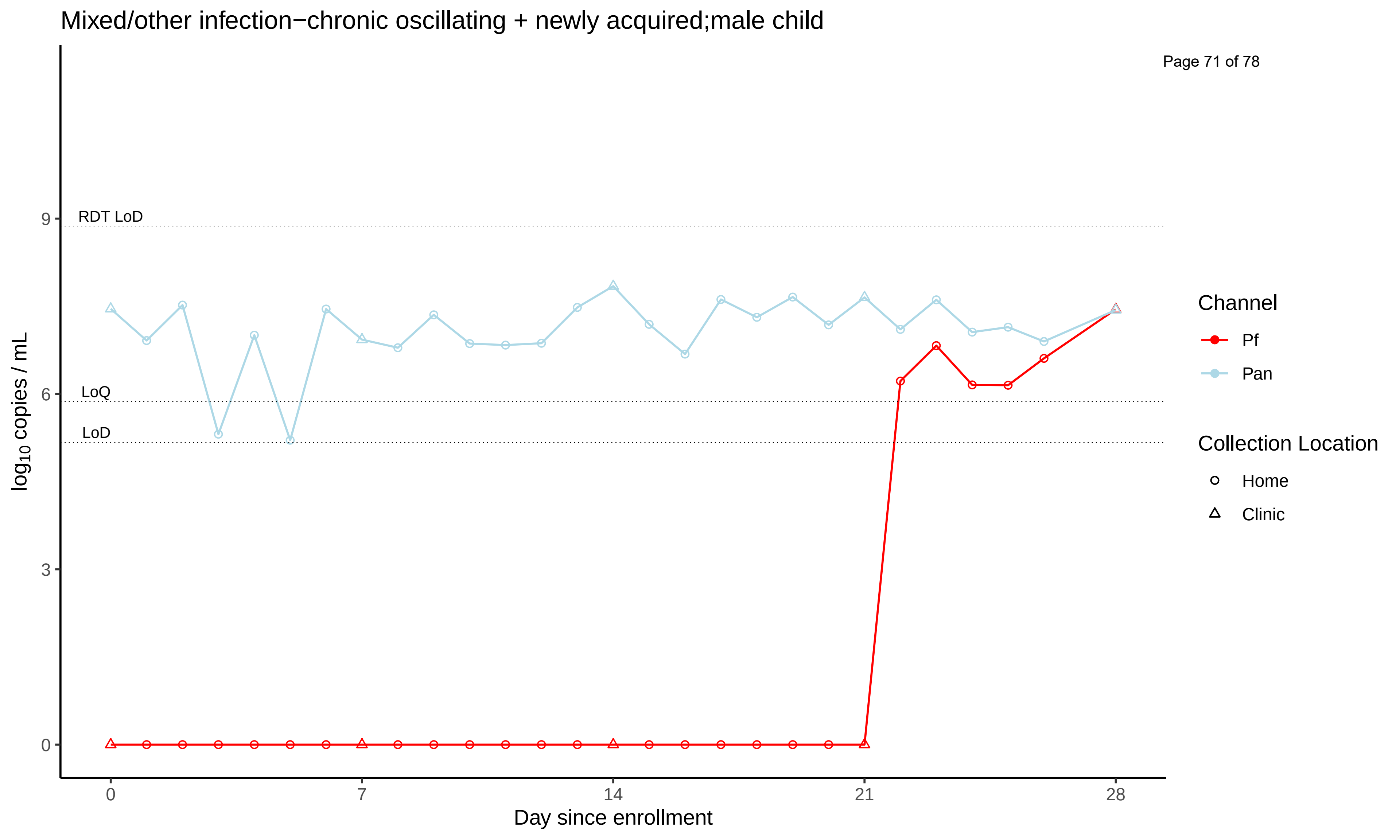
Mixed/other infection−chronic oscillating + newly acquired;male child
RDT LoD
LoQ
LoD
9
6
3
0
log_{10} copies / mL
0
7
14
21
28
Day since enrollment
Channel
Pf
Pan
Collection Location
Home
Clinic

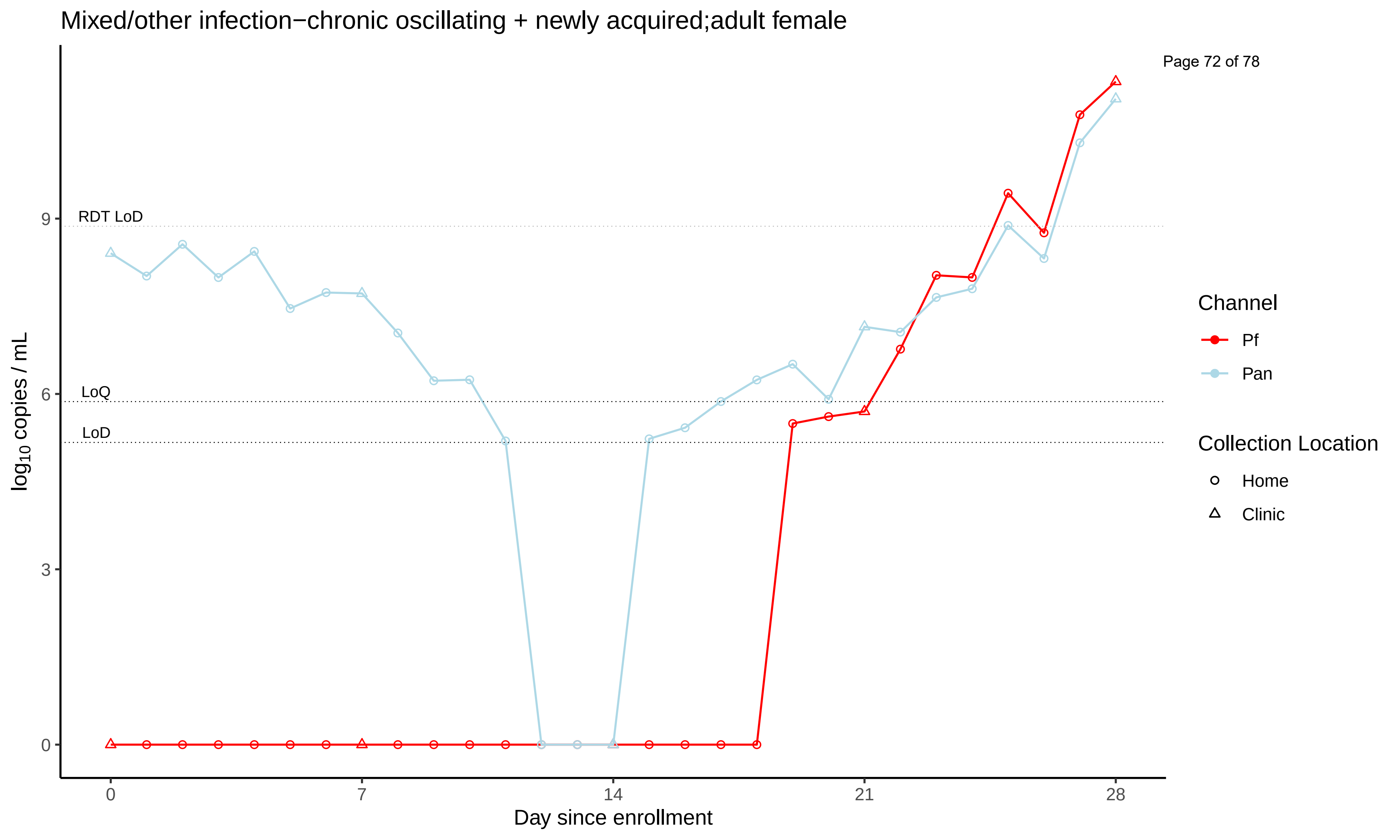
Mixed/other infection−chronic oscillating + newly acquired;adult female
RDT LoD
LoQ
LoD
9
6
3
0
log10 copies / mL
0
7
14
21
28
Day since enrollment
Channel
Pf
Pan
Collection Location
Home
Clinic

Mixed/other infection−chronic oscillating non−Pf + newly acquired Pf;adult male

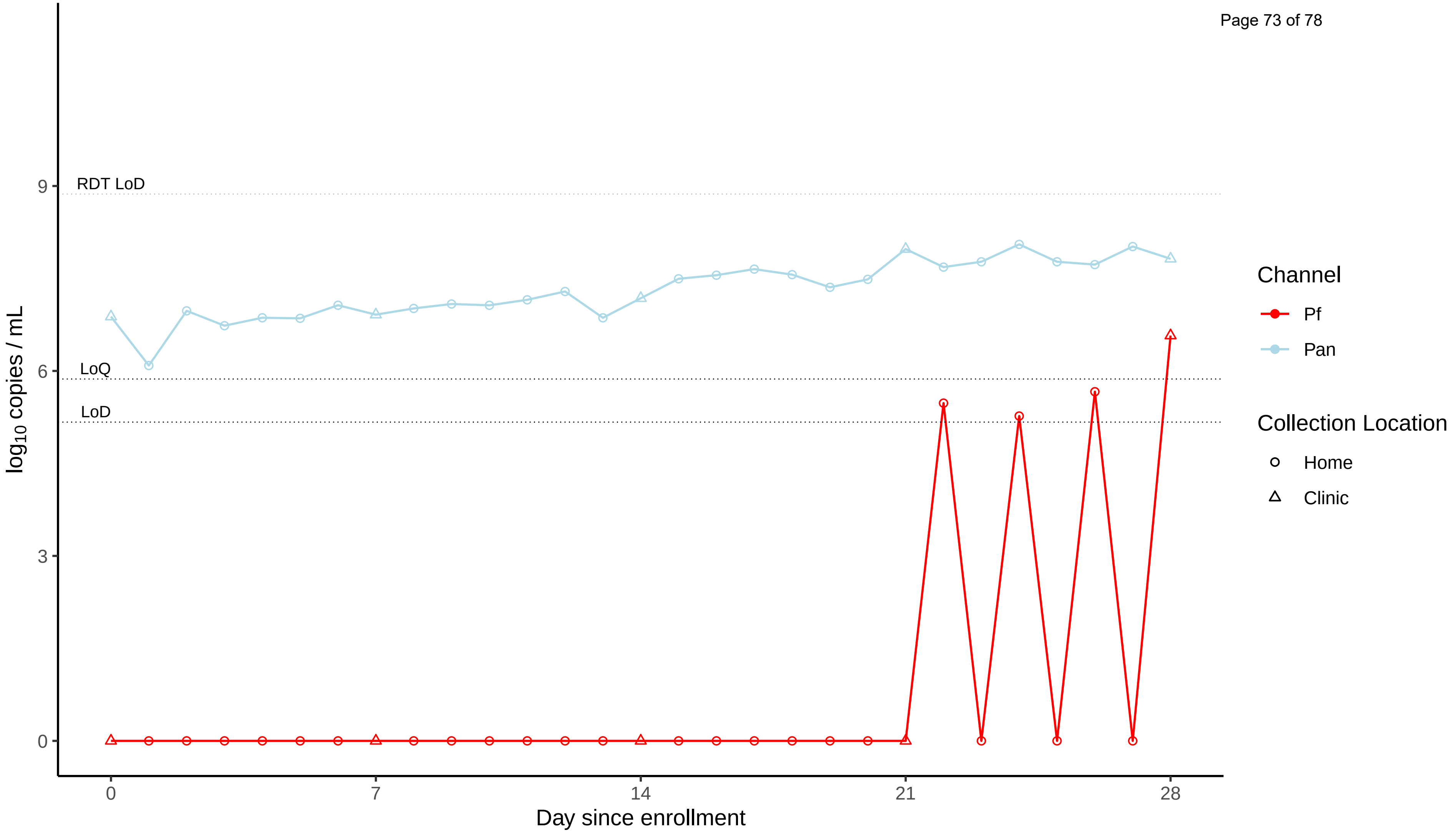

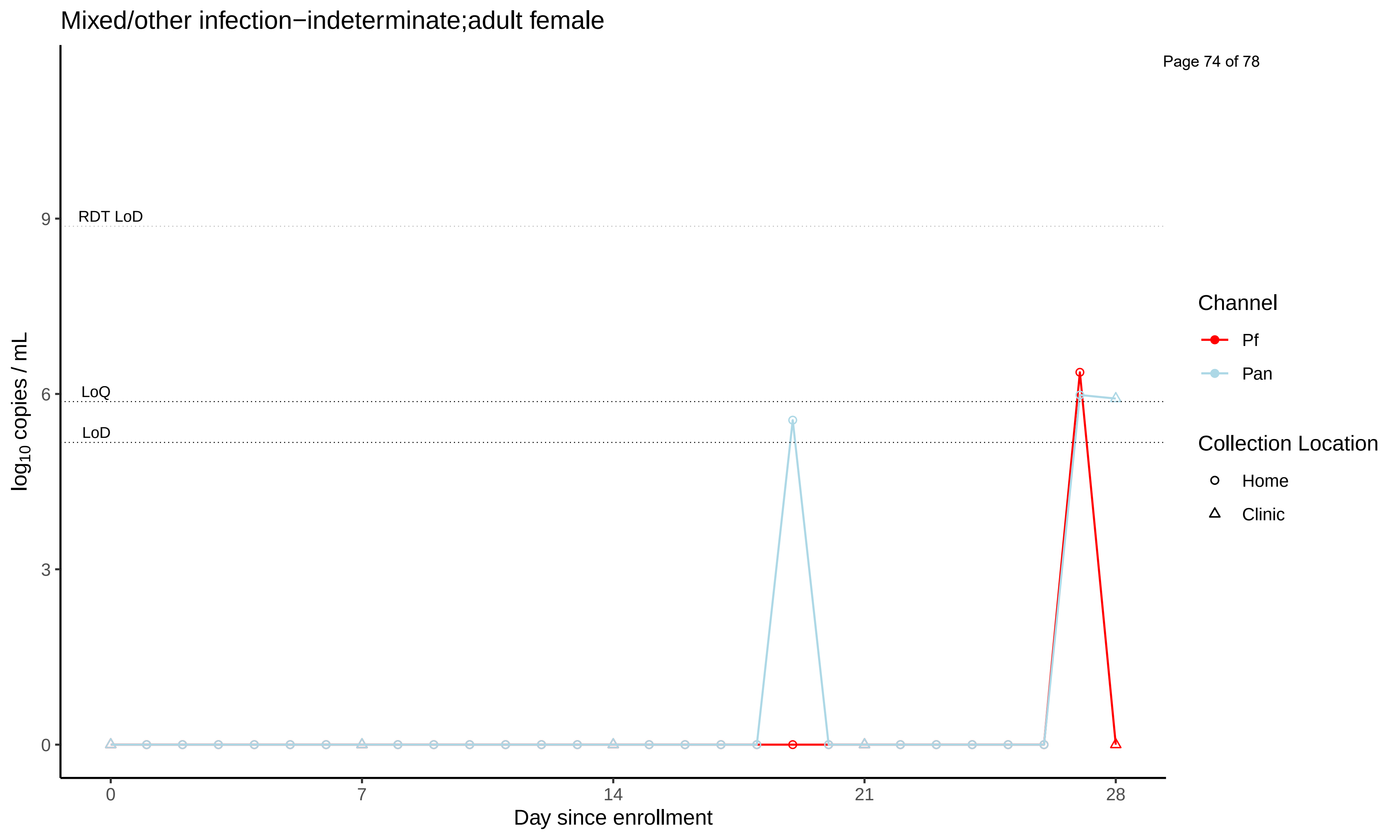

Mixed/other infection−indeterminate;adult female
RDT LoD
LoQ
LoD
log10 copies / mL
0
3
6
9
0
7
14
21
28
Day since enrollment
Channel
Pf
Pan
Collection Location
Home
Clinic

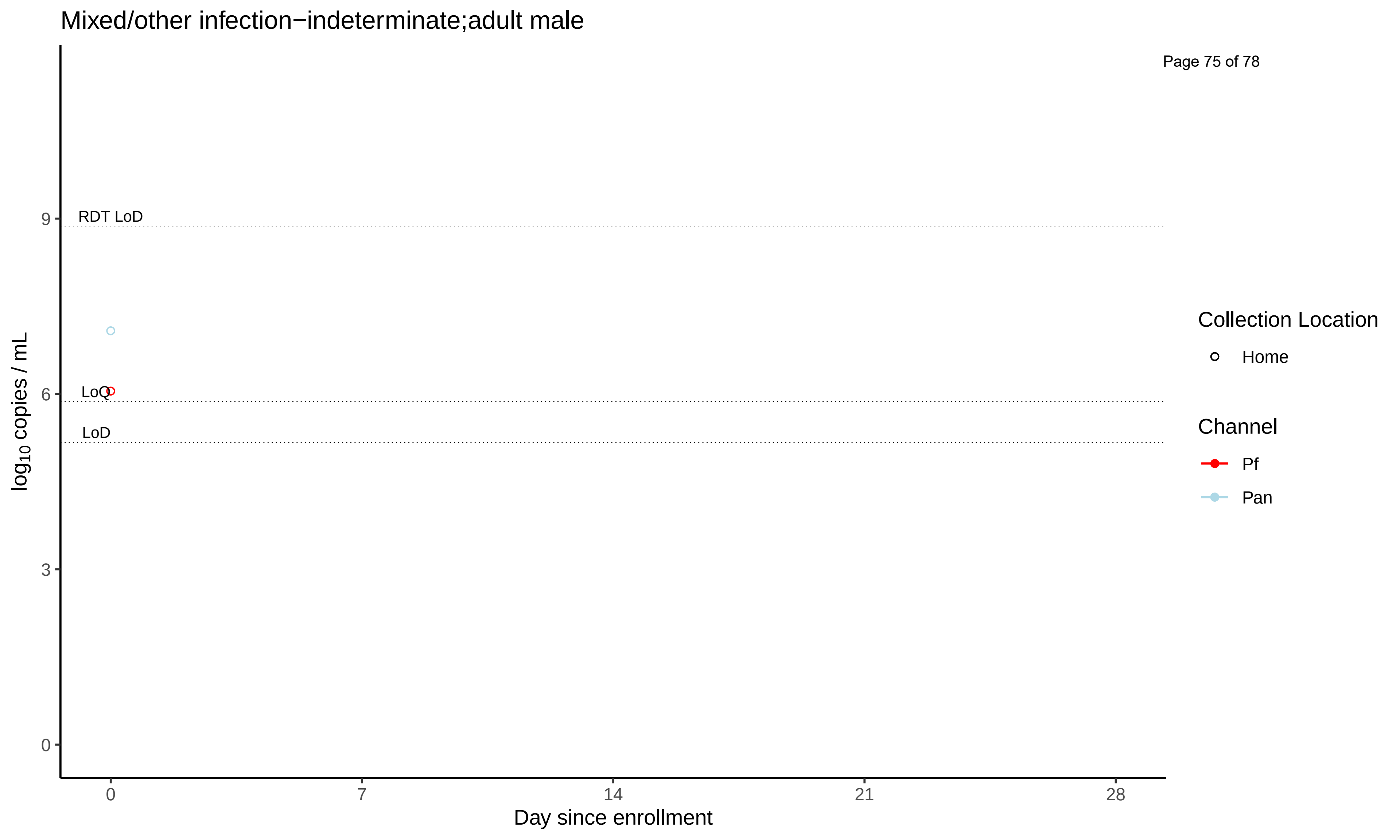
Mixed/other infection−indeterminate;adult male
RDT LoD
LoQ
LoD
9
6
3
0
0
7
14
21
28
Day since enrollment
log10 copies / mL
Collection Location
Home
Channel
Pf
Pan

Mixed/other infection−indeterminate;adult female

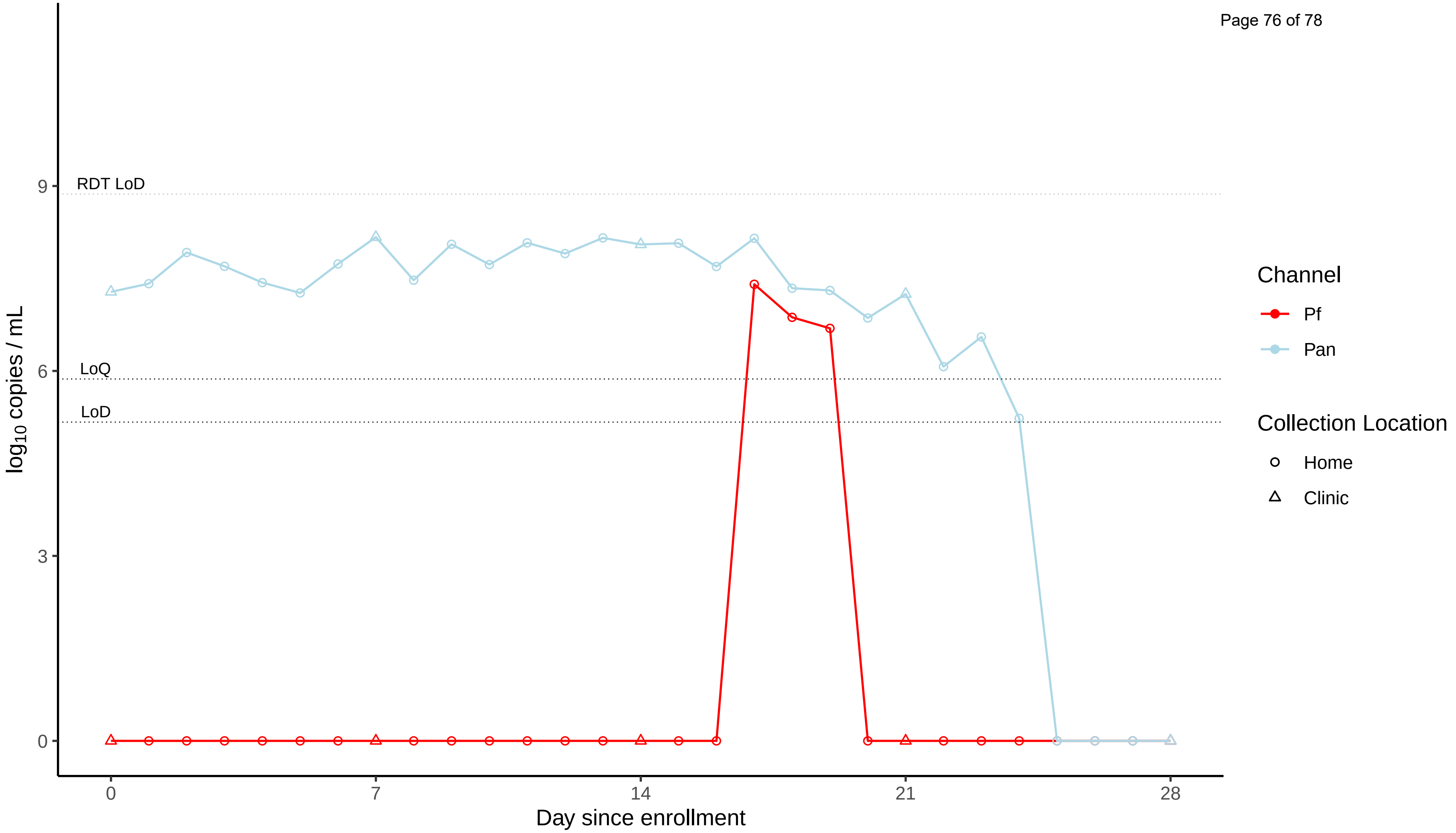

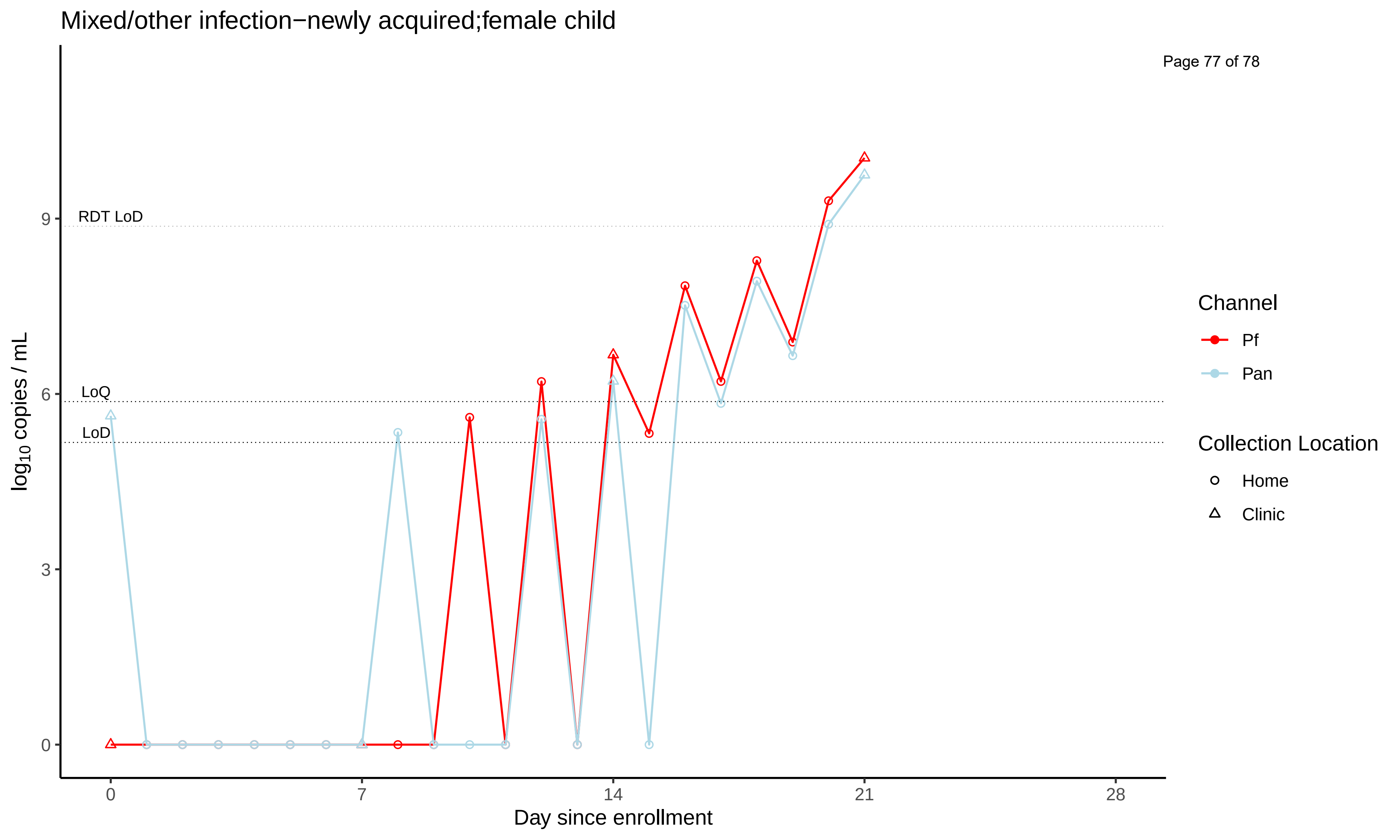
Mixed/other infection−newly acquired;female child
Page 77 of 78
RDT LoD
LoQ
LoD
log10 copies / mL
0
3
6
9
0
7
14
21
28
Day since enrollment
Channel
Pf
Pan
Collection Location
Home
Clinic

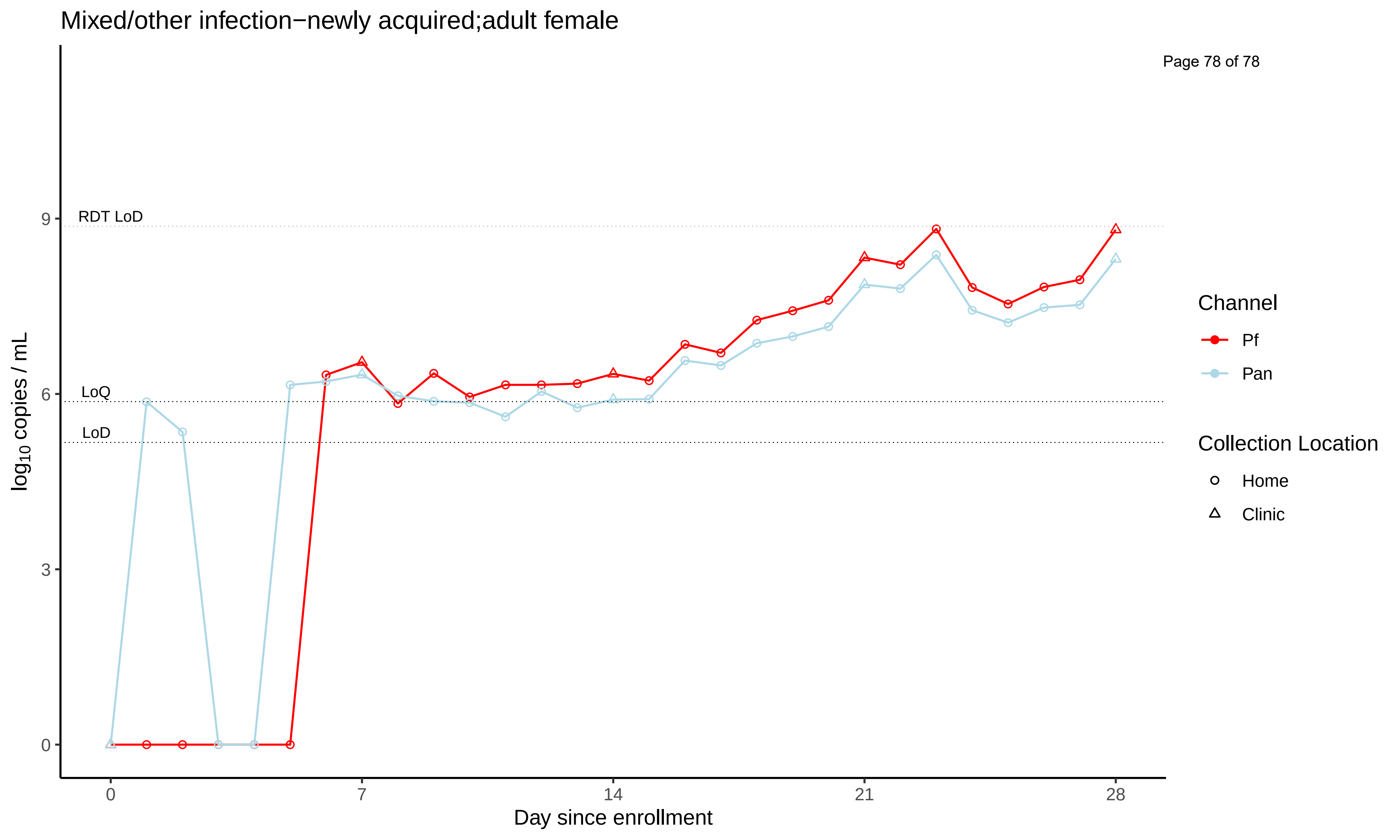
Mixed/other infection−newly acquired;adult female
RDT LoD
LoQ
LoD
9
6
3
0
log10 copies / mL
0
7
14
21
28
Day since enrollment
Channel
Pf
Pan
Collection Location
Home
Clinic
